# Cavity‐Based Discovery of New Fatty Acid Photodecarboxylases

**DOI:** 10.1002/cbic.202400631

**Published:** 2024-11-09

**Authors:** Stefan Simić, Marco Cespugli, Michael C. Hetmann, Ursula Kahler, Valentina Jurkaš, Marikagiusy Di Giacomo, Maria E. Russo, Antonio Marzocchella, Christian C. Gruber, Bettina M. Nestl, Christoph K. Winkler, Wolfgang Kroutil

**Affiliations:** ^1^ Institute of Chemistry University of Graz Heinrichstraße 28 8010 Graz, Austria; ^2^ Innophore GmbH Am Eisernen Tor 3 8010 Graz Austria; ^3^ Dipartimento di Ingegneria Chimica dei Materiali e della Produzione Industriale Università degli Studi di Napoli Federico II P.le V. Tecchio 80 80125 Napoli Italy; ^4^ CNR-Istituto di Scienze Tecnologie per l'Energia e la Mobilità Sostenibili P.le V. Tecchio 80 80125 Napoli Italy; ^5^ Field of Excellence BioHealth – University of Graz 8010 Graz Austria; ^6^ BioTechMed Graz 8010 Graz Austria

**Keywords:** Photocatalysis, Biocatalysis, Photodecarboxylase, Enzyme discovery

## Abstract

Light‐dependent fatty acid photodecarboxylases (FAPs) hold significant potential for biotechnology, due to their capability to produce alka(e)nes directly from the corresponding (un)saturated natural fatty acids requiring light as the only reagent. This study expands the family of FAPs through cavity‐based enzyme discovery methods. Thirty enzyme candidates with potential photodecarboxylation activity were identified by matching the cavities of four related template structures against the Protein Data Bank's flavoproteins, a library of proteins identified *via* the Foldseek Search Server, and homology models of sequences resulting from BLAST. Subsequent docking experiments narrowed this library to ten promising enzymes, which were expressed and assessed *in vitro*, identifying four photodecarboxylases. Out of these enzymes, the GMC oxidoreductase from *Coccomyxa* sp. Obi (*Co*FAP) was characterized in detail, which revealed high activity in the decarboxylation reactions of palmitic acid and octanoic acid and a broad pH tolerance (pH 6.5–9.5).

## Graphical abstract

Fatty acid photodecarboxylases (FAPs) show biotechnological promise by producing alka(e)nes from natural fatty acids. Using a cavity based search, 30 distant or unrelated enzymes with potential FAP‐activity were identified either from the PDB‘s flavoproteins of from homology models. Docking experiments and in vitro characterization led to the discovery of four photodecarboxylases.

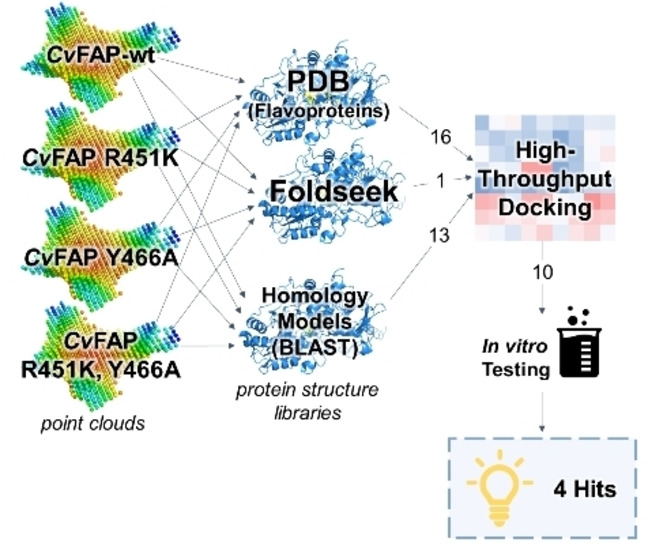

## Introduction

**Figure 1:**
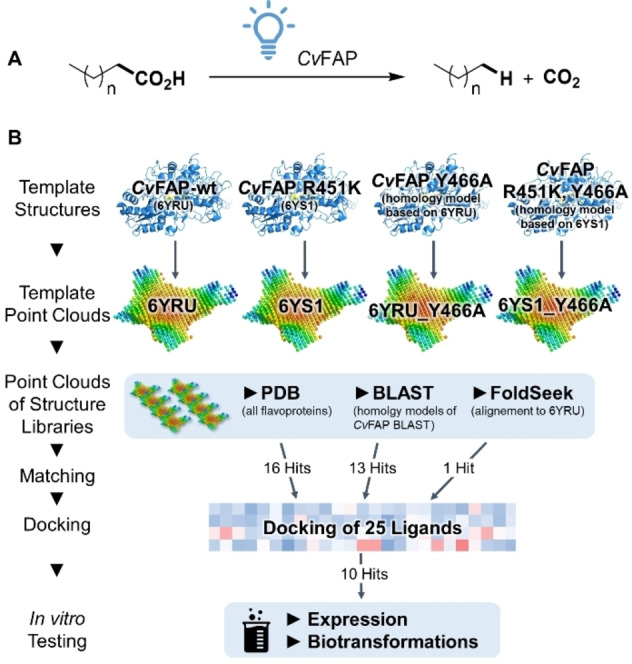
A: Photodecarboxylation or fatty acids catalyzed by *Cv*FAP. B: Workflow for the cavity‐based discovery of new photodecarboxylases consisting of the procreation of four template point‐clouds (exemplary point clouds are shown), matching experiments with three sources of protein structures (the FoldSeek structures overlap with the PDB structures), high throughput docking experiments and *in vitro* testing.

Fatty acid photodecarboxylases (FAPs)^[1]^ are FAD‐dependent photoenzymes[^2^, ^3^, ^4^, ^5^] which catalyze the light‐dependent decarboxylation of carboxylic acids (Figure 1A).[^1^, ^6^] They belong to the class of glucose–methanol–choline (GMC) oxidoreductases, which share a similar structure, but catalyze a diverse range of redox reactions, including dehydrogenase and oxidase activities.^[7]^ The most well‐characterized FAP originates from the unicellular photosynthetic microalgae *Chlorella variabilis* NC64 A (*Cv*FAP)[^1^, ^8^, ^9^] and has received significant attention due to its potential to produce alkanes and alkenes from natural fatty acids as direct substitutes for fossil fuels (drop‐in biofuels).[^4^, ^10^, ^11^] Several variants have been developed to extend the substrate scope of *Cv*FAP to various structurally diverse carboxylic acids, including short‐chain functionalized aliphatic, and aromatic carboxylic acids.[^12^, ^13^, ^14^, ^15^, ^16^, ^17^, ^18^, ^19^]

Despite advances in reaction and process engineering for enhancing the productivity of the photodecarboxylation,[^15^, ^20^, ^21^, ^22^, ^23^, ^24^, ^25^, ^26^, ^27^] the intrinsic photolability of *Cv*FAP poses a challenge for its preparative application.[^28^, ^29^] Consequently, the photodecarboxylation activities of other GMC oxidoreductases and their ancestral forms were explored in several studies.[^30^, ^31^, ^32^, ^33^]

To identify novel FAPs decarboxylating a broad range of fatty acids, herein a computational approach based on the Catalophore^TM^ platform was employed to complement the previously used sequence‐based methods.[^34^, ^35^] The method computes physico‐chemical properties at virtually introduced points in the empty space within the enzyme's active site, forming a point cloud (catalophore) that describes the geometry and physico‐chemical characteristics of the active site. The point cloud of the template cavity is matched against the point clouds of target libraries of crystal structures or homology models. Beyond enzyme discovery,[^34^, ^35^] these point clouds have been applied for studying structural features of enzymes,^[36]^ investigating protein surfaces^[37]^ and for swiftly identifying inhibitors for specific enzymes.^[38]^ As this technique is not limited by the protein sequence or overall protein fold as a similarity criterium, it facilitates the identification of activity analogous to the template in enzymes that sequence‐based searches would overlook.

Herein the following workflow was applied for the discovery of novel enzymes with photodecarboxylation activity (Figure 1B): To increase the probability of identifying suitable matches, the structure of *Cv*FAP‐wt and three variants were used to generate four structurally and electronically different template point clouds. These templates were matched against the cavity‐libraries of three sources of protein structures, the first being all flavoproteins in the Protein Data Bank (PDB).^[39]^ As a second more focused structure library the Foldseek Search Server^[40]^ was used to identify proteins with structures close to the structure of *Cv*FAP (6YRU).^[9]^ Finally, to include enzymes without a published structure, but close to *Cv*FAP in sequence space, homology models of the most promising results of a BLAST (Basic Local Alignment Search Tool) search^[41]^ were created based on the *Cv*FAP wild‐type sequence.^[9]^ The best 30 matches were first evaluated *in silico via* extended docking studies and finally the ten most promising new FAPs were characterized *in vitro*.

## Results and Discussion

### Template Creation

Four protein structures were used to generate the search‐template point clouds, the first being the crystal structure of wild‐type *Cv*FAP (PDB ID: 6YRU^[9]^). As the wild‐type has a quite narrow binding tunnel, the structure of its Y466A variant, which is reported to accept bulkier substrates,[^12^, ^14^] was generated as homology model from 6YRU and used as second template structure. The arginine at 451 is conserved in the known fatty acid photodecarboxylases (see Figure S3), but was reported not to be crucial for the reactivity.^[9]^ As the crystal structure of the *Cv*FAP R451K variant is available (PDB ID: 6YS1^[9]^) it was included as third template to consider variation at this position. Finally, also the Y466A−R451K double variant was prepared as homology model of 6YS1 as template structure.

**Figure 2:**
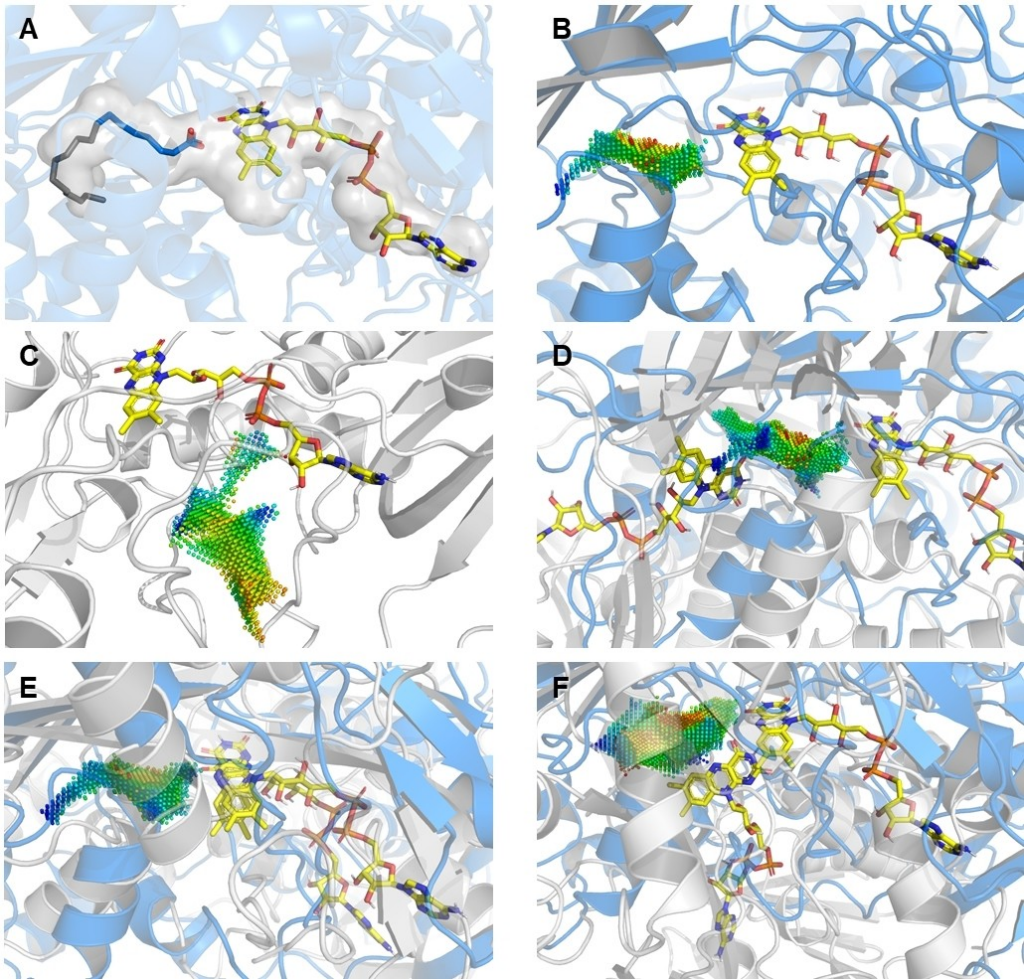
A: Active site of *Cv*FAP (PDB ID: 6YRU) with bound stearic acid (dark grey) and the truncated substrate that was used for cavity procreation (blue); B: Point cloud in the active site of *Cv*FAP (PDB ID: 6YRU); C: False‐positive match: this point cloud is not aligned with the FAD (PDB ID: 2GVC); D: False‐positive match: the point clouds of *Cv*FAP (PDB ID: 6YRU) and the target structure (PDB ID: 5FNO) are aligned, but the flavins point into different directions (*i. e*., the point clouds match, but the cofactor is on the opposite side of the point cloud). E: Alignment of *Cv*FAP (PDB ID: 6YS1) and *CO*FAP (BLAST candidate homology model). F: Alignment of *Cv*FAP (PDB: 6YRU) and the Foldseek hit (PDB ID: 4HA6). The structure of *Cv*FAP (blue) has a similar orientation in all figures. Any aligned structure is depicted in light grey.

As stearic acid is the ligand in all template structures and the template cavity is calculated around the protein‐bound ligand, the resulting cavity would mostly have the features of a binding site for long aliphatic molecules. To avoid over‐representing the apolar part of the structure's cavities in the template point‐clouds, the ligand was truncated computationally to octanoic acid using YASARA and energy minimization was performed for all structures (Figure 2A and B). This resulted in smaller template point‐clouds that put more emphasis on the carboxylate binding region of the active site, avoiding high matching scores with enzymes that bind long aliphatic chains. The four template point clouds were procreated based on these four structures for the cavity‐based search (see Figure S4 for a comparison of the point clouds).

### Cavity Matching with the PDB Database (PDB Candidates)

The four template point clouds were matched against the cavities of all flavoproteins in the PDB. The matching results were filtered by total score (<0.05), with overlaps above 70 % for cavity 1 (*Cv*FAP template) to cavity 2 (target enzyme) and above 10 % (or 30 % for larger candidate pools) for cavity 2 to cavity 1, to also consider large cavities with parts that are well overlapped with the *Cv*FAP template cavity. The matching scenes were visually inspected in PyMOL to eliminate cavities not aligned with the isoalloxazine ring and to ensure the electrostatic point cloud adjacent to the cofactor is stabilizing towards negative charge (Figure 2C). This also allowed to exclude cavities with a high total score, where the flavin cofactors are facing into opposite directions in the matching scene (Figure 2D). Overall, sixteen candidates were selected based on visual inspection (Table S1, Entries 15–30). The properties of the electrostatic point‐cloud (stabilization of negative charge close to the flavin core) and the presence of hydrogen‐bond donating amino acids in the vicinity of the flavin core were the principal parameters taken into consideration during the visual inspection. Out of the sixteen candidate cavities, seven belong to the *Homo sapiens* D‐amino‐acid oxidase, one to a D‐amino‐acid oxidase from *Sus scrofa*, two to monoamine oxidase B from *Homo sapiens* (wild type and Y435F variant) and the remainders are flavoproteins from various bacterial strains.

### Cavity Matching with the Foldseek Candidates

As a second strategy, the accelerated protein structure alignment tool Foldseek Search Server was used to identify further candidates with high similarity in the tertiary structure (fold).^[40]^ *Cv*FAP (6YRU^[9]^) was used as a query structure and the search was run against the PDB, generating 278 alignments (eight were structures corresponding to *Cv*FAP). Cavity calculation and comparison to the four query cavities was done as before. Since no candidates were found based on previously described identifiers (electrostatic point cloud, hydrogen bond donating residues, flavin orientation), the top 20 candidates generated in the Foldseek output file were inspected visually, regardless of the total matching score with the query cavities (Table S2). The foldseek match with the PDB ID 4HA6^[42]^ (Table S1, entry 14) was chosen as a plausible candidate for docking studies due to the area of positive charge in the vicinity of FAD, created by adjacent histidine residues (H460, H461) acting as hydrogen bond donors (Figure 2F).

### Cavity Matching with the BLAST‐Search Candidates

To expand the search for putative photodecarboxylases among proteins with undetermined structures, a sequence similarity search was performed for phylogenetically related enzymes. This was followed by generating homology models for subsequent cavity procreation and matching experiments. Initially, a BLAST search on the NCBI's non‐redundant (nr) protein database was performed,^[41]^ using the sequence from PDB ID: 6YRU^[9]^ as query. The results were filtered by applying cut‐off values of 30 % sequence identity and 80 % bidirectional coverage (query) and the search was limited to 100,000 hits, with a noticeable increase in sequences not meeting the sequence identity requirement after approximately 80,000 hits.

To reduce the size of the obtained dataset, MMSEQS2 clustering^[43]^ was applied using a sequence identity cut‐off of 70 % (*i. e*. sequences within a cluster should have at least 70 % sequence identity). As R451 in *Cv*FAP is critical for substrate binding and conserved in all known FAPs,[^1^, ^9^, ^32^, ^44^] the sequences of the 9800 cluster representatives were then aligned and filtered based on the presence of an equivalent arginine residue. This resulted in a final list of 234 representative sequences (Figures S1 and S2).

Homology models were created for this set of sequences using the structure of the *Cv*FAP wild‐type (PDB ID: 6YRU^[9]^) as the template. Cavity calculation and comparison were performed as for the PDB candidates. After visual inspection of matching scenes, 12 candidates were chosen, all with sequence identities between 40 % and 62 % to *Cv*FAP (Table S1, Entries 1–12 and Figures S1 and S2).

Multiple sequence alignment of candidate sequences additionally revealed, that in addition to residue R451 (criterion used for filtering the BLAST search results), C432 was present in all sequences, and Y466 was conserved in all green algae enzymes, whereas red algae enzymes have lysine in that position (Figure S3).^[45]^ These three residues (R451, C432, Y466) are reportedly conserved among the class of fatty‐acid photodecarboxylases.^[9]^

Further filtering was performed, using the “protein existence” entry in the UniProt database^[46]^ as criterion, *i. e*., only reviewed proteins with confirmed expression and/or characterization were in the library. The BLAST search was repeated on this filtered database with the same criteria as previously described resulting in an output of 60 sequences. Homology models were created using *Cv*FAP (PDB ID: 6YRU^[9]^) as the template and cavity procreation and matching were performed as described previously. For the analysis of the matching experiments, the total score of below 0.05 and overlap of cavity 2 with cavity 1 above 10 % were used as a filtering criterion. Upon visual inspection, candidates were chosen based on relative positioning of cofactors in the matching scene and the presence of an arginine residue in the vicinity of FAD (for a positive example see Figure 2E). Specifically, the matching of the C8‐truncated *Cv*FAP (6YRU^[9]^) cavity with patulin synthase from *Aspergillus clavatus* (UniProt ID: A1CFL2) yielded a total score of 0.047 (Table S1, Entry 13). Moreover, it was observed that FAD molecules of the two enzymes were well aligned and that residue R433 of patulin synthase occupies a position in the active site analogous to R451 in *Cv*FAP (Figure S5).

### Molecular Docking and Evaluation of Cavities

A total of 30 cavity candidates were selected from previously described cavity matching experiments (Table S1). To further investigate their potential for photodecarboxylation, molecular docking was performed using a set of 25 structurally diverse ligands, including aliphatic, aromatic, and functionalized carboxylic acids (Figure S6). The energy and the distance between the carbonyl carbon of the substrate and the N5 nitrogen of the flavin cofactor were tracked for each cluster. Clusters with minimal distance and lower (negative) binding energy were taken into consideration when evaluating the docking results. The absolute value of the ratio of binding energy and distance was taken as a combined score, as it favors low (negative) binding energies as well as low distances.

**Figure 3:**
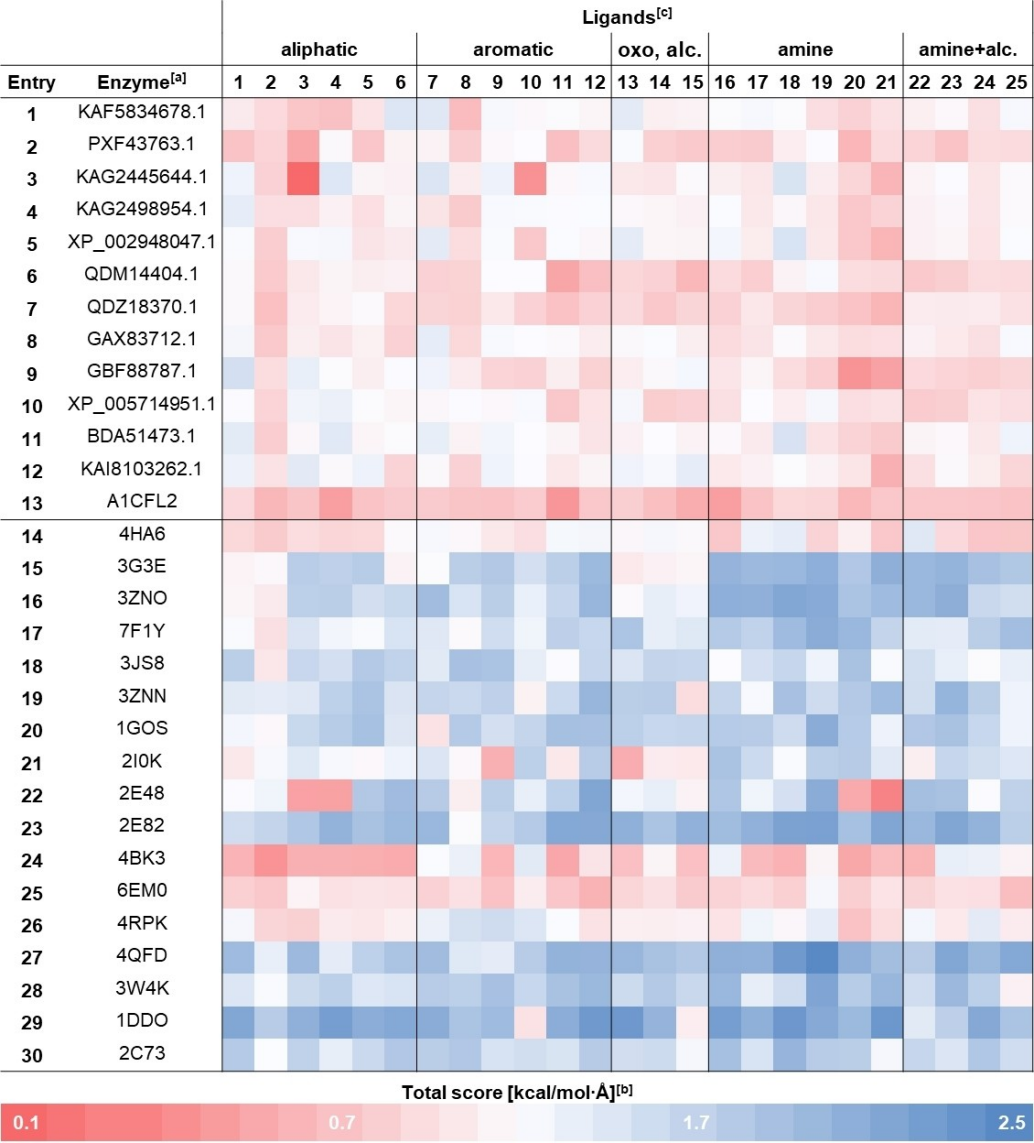
The combined scores (|E/d|) in the docking between candidate cavities and set of carboxylic acid ligands shown as heat map. For a list of the sum of the combined scores, see Table S3 [a] Entries 1–12 show GenBank Protein IDs, entry 13 shows a UniProt ID and entries 14–30 show PDB IDs. [b] The combined scores were calculated as an absolute value of the ratio of energy (kcal⋅mol^−1^) and distance (COOH−N5, Å). For each ligand, the cluster with minimal distance and the binding energy corresponding to that cluster were taken. [c] oxo, alc: ketones or alcohols; amine+alc.: 1,2‐hydoxyamines. KAF5834678.1: *Ds*FAP; KAG2498954.1: *Ed*FAP; XP_002948047.1: *Vc*FAP; BDA51473.1: *CO*FAP; KAI8103262.1: *Pi*FAP.

Figure 3 shows a grouping of high combined scores among PDB search candidates (entries 15–30), whereas the BLAST search candidates (entries 1–13) and the Foldseek candidate (entry 14) showed predominantly low combined scores in comparison.

It is noteworthy, that seven PDB candidates referred to the same enzyme, *Homo sapiens* D‐amino acid oxidase (Figure 3, entries 15, 16, 19, 22, 23, 27, 28), which yielded high combined scores for the majority of tested substrates. The sum of combined scores over the entire set of examined substrates was calculated for each enzyme. Overall, the PDB candidates showed higher total combined scores than the BLAST candidates (see Table S3), which might be because to the narrow substrate binding tunnel of fatty acid photodecarboxylases prohibits substrates of different shape or aromatic substrates from efficient binding. Interestingly, almost all BLAST candidates showed good binding of palmitic acid (**1**). From the BLAST candidates, five enzymes with highest sum of combined scores (Figure 3, entries 1, 4, 5, 11 and 12; see Table 1, entries 1–5) were picked along with five enzymes with highest combined scores among the PDB candidates (Figure 3, entries 17, 18, 21, 23 and 29; see Table 1, entries 6–10).

**Table 1:** The list of enzyme candidates extracted from cavity‐search experiments.

Entry	Enzyme identifier (abbreviation)^[a]^	Enzyme name	Source organism
1	KAF5834678.1 (*Ds*FAP)	GMC oxidoreductase‐domain‐containing protein	*Dunaliella salina*
2	KAG2498954.1 (*Ed*FAP)	GMC oxidoreductase‐N‐domain‐containing protein	*Edaphochlamys debaryana*
3	XP_002948047.1^[32]^ (*Vc*FAP)	GMC oxidoreductase‐N‐domain‐containing protein	*Volvox carteri f. nagariensis*
4	BDA51473.1 (*CO*FAP)	Oxygen‐dependent choline dehydrogenase	*Coccomyxa* sp. *Obi*
5	KAI8103262.1 (*Pi*FAP)	Hypothetical protein M9435_004601	*Picochlorum sp. BPE23*
6	7F1Y[^47^, ^48^]	L‐lactate oxidase	*Aerococcus viridans*
7	3JS8^[49]^	Solvent‐stable cholesterol oxidase	*Chromobacterium* sp*. DS‐1*
8	2I0 K^[50]^	Cholesterol oxidase (H121 A variant)	*Brevibacterium sterolicum*
9	2E82[^51^, ^52^]	D‐amino‐acid oxidase	*Homo sapiens*
10	1DDO[^53^, ^54^]	D‐amino‐acid oxidase from pig kidney	*Sus scrofa*

^[a]^ For entries 1–5, identifiers refer to GenBank Protein IDs;^[55]^ for entries 6–10 identifiers refer to PDB IDs.^[39]^

### Expression and Activity of the Identified Candidates

The genes of this final list of candidates (Table 1), were ordered in pET28a(+) vectors (see supporting information) and expressed in *E. coli* BL21(DE3). SDS‐PAGE gel analysis of the disrupted cells (Figure S7) showed soluble expression for the cholesterol oxidases (PDB ID: 2I0 K, 3JS8)[^49^, ^50^] and the protein samples *CO*FAP (BDA51473.1) and *Vc*FAP (XP_002948047.1). Reproducing the expression procedures from literature for the remaining PDB candidates (1DDO, 2E82, 7F1Y) did not lead to significant improvement (Figure S8).[^48^, ^52^, ^54^] However, the presence and activity of these enzymes was confirmed in assays with their respective natural substrates (Table S4).

**Scheme 1:**
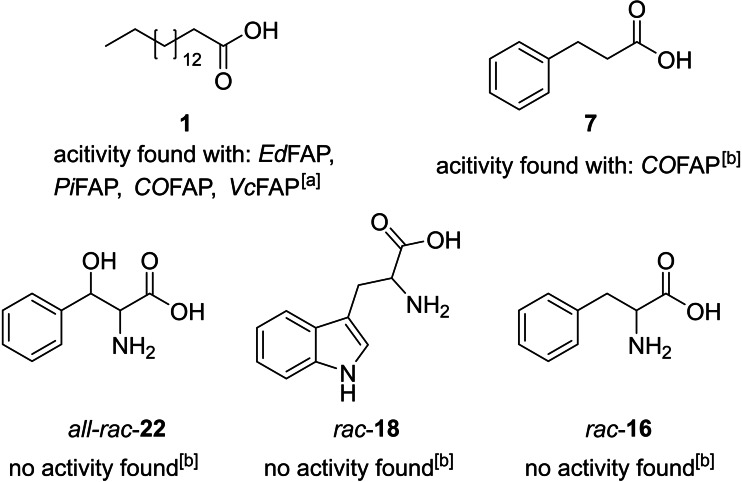
Substrates investigated for photodecarboxylation activity of enzyme candidates. Enzyme candidates showing activity are enlisted below the substrate structures. In contrast to the dockings which were done with the individual enantiomers of the ligands (see Figure S6) the substrates were used as racemic mixtures, as depicted in the biotransformations. Substrate *all‐rac*‐**22** corresponds to the ligands **22–25**; substrate *rac*‐**18** corresponds to ligands **18** and **19**; and substrate *rac*‐**16** corresponds to ligands **16** and **17**. [a] Reaction conditions: Substrate (10 mM), DMSO (10 % v/v), KPi buffer (100 mM, pH 8, 900 μL total volume) and lyophilized cell lysate (10 mg mL^−1^, for individual enzyme amounts, see Table S5) were illuminated at 455 nm (36 μmol L^−1^ s^−1^), 25 °C, 500 rpm, for 20 h. [b] Reaction conditions: Substrate (10 mM), KPi buffer (100 mM, pH 8, 1 mL total volume) and lyophilized cell lysate (10 mg mL−1) were illuminated under identical conditions as shown above. The buffer was purged with N_2_ for 1 h and the reactions were performed under N_2_ atmosphere. All samples were prepared in duplicates.

To ensure covering a broad range of different substrate structures, the enzyme candidates (Table 1) were tested for photodecarboxylation of five structurally diverse substrates from the docking experiments (Scheme 1). Established photodecarboxylation conditions were applied for this screening, including illumination close of the maximum of oxidized flavin (455 nm) at a photon flux of 36 μmol L^−1^ s^−1^ at 25 °C and in phosphate buffer at pH 8.^[56]^

Note, that transformations with α‐amino acids (*all‐rac‐* **22**, *rac‐* **16** and *rac*‐**18**) were prepared under an inert atmosphere (N_2_) to prevent the natural oxygen‐dependent amino‐acid oxidase activity of the tested amino acid oxidases. No decarboxylation products of the tested amino acids were observable by HPLC‐MS (or GC‐MS after derivatization) under the examined conditions. A low‐intensity peak corresponding to ethylbenzene, the decarboxylation product of 3‐phenylpropanoic acid **7**, was detected (Figure S41) in the samples containing GMC oxidoreductase from *Coccomyxa* sp. Obi (*CO*FAP; Scheme 1, substrate **7**).

The GC‐MS analysis of reaction mixtures containing palmitic acid (ligand **1**) revealed low decarboxylation activity for four BLAST search candidates (Table 1, entries 1–3 and 5; Figure S9).

The highest product formation was observed using the oxygen‐dependent choline oxidase from *Coccomyxa* sp. Obi (*CO*FAP), followed by protein M9435_004601 from *Picochlorum sp. BPE23* (*Pi*FAP) and the GMC oxidoreductase from *Volvox carteri f. nagariensis* (*Vo*FAP). Product formation was also observable in the reaction mixture containing GMC oxidoreductase from *Edaphochlamys debaryana* (*Ed*FAP). Although GMC oxidoreductase from *Volvox carteri f. nagariensis* (*Vc*FAP) was listed as a hypothetical protein on the GenBank database, its photodecarboxylation activity had been previously reported in the literature.^[32]^ *Ed*FAP, *Pi*FAP and *Vc*FAP showed higher activity on the longer stearic acid (Figure S10).

**Figure 4:**
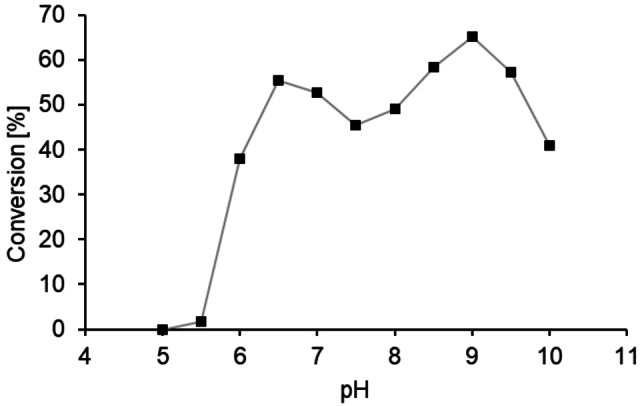
pH profile of fatty‐acid photodecarboxylase from *Coccomyxa* sp. Obi (*CO*FAP) displayed as conversion in the photodecarboxylation of palmitic acid. Reaction conditions: palmitic acid (10 mM), DMSO (10 % v/v), buffer (900 μL), *CO*FAP (10 mg mL^−1^ lyophilized cell lysate, containing 0.1 mg mL^−1^ *CO*FAP, estimated by SDS PAGE densitometry), total volume 1 mL. Illumination was performed at 455 nm (36 μmol L^−1^ s^−1^), 25 °C, 500 rpm, for 20 h. All data points represent mean values of duplicate determinations.

As none of the tested PDB‐candidates (Table 1, entries 6–10) showed any photo‐promiscuous activity, their reactivity under altered reaction conditions was not further investigated and the work was focused on characterizing the most promising enzyme candidate among the BLAST candidates: the oxygen‐dependent choline oxidase from *Coccomyxa* sp. Obi (*CO*FAP; Table 1, entry 4). Unlike *Cv*FAP,[^1^, ^57^] *CO*FAP displayed high activity in the photodecarboxylation of palmitic acid over a broad pH range (pH 6.5–9.5, Figure 4).

**Figure 5:**
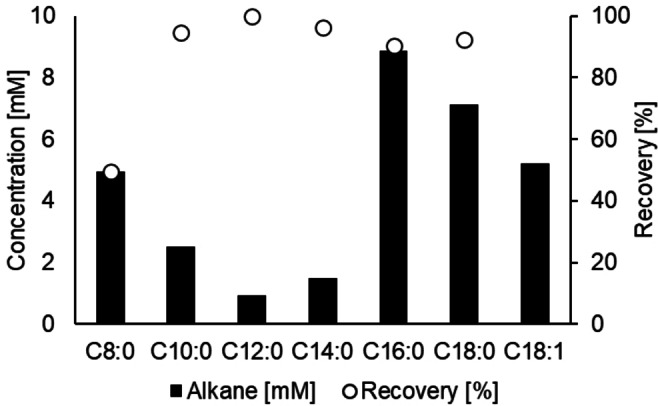
Photodecarboxylation of fatty acids catalyzed by fatty‐acid photodecarboxylase from *Coccomyxa* sp. Obi (*CO*FAP). Reaction conditions: fatty acid (10 mM), DMSO (10 % v/v), Tris⋅HCl buffer (100 mM, pH 8.5, 900 μL), *CO*FAP (10 mg mL^−1^ lyophilized cell lysate, containing 0.22 mg mL^−1^ *CO*FAP, estimated by SDS PAGE densitometry), total volume 1 mL. Illumination was performed at 455 nm (36 μmol L^−1^ s^−1^), 25 °C, 500 rpm, for 20 h. For C18 : 1 only substrate depletion was measured. All data points represent mean values of duplicate or triplicate determinations.

To explore potential differences in fatty acid preference between *Cv*FAP and *CO*FAP, a set of saturated fatty acids (C6:0 to C18:0) was decarboxylated under previously established reaction conditions (Figure 5, hexanoic acid is not shown as no product formation was found).

The results showed that in addition to palmitic acid (98 % conversion) also octadecanoic acid was accepted with high conversion (77 %). Despite low recovery (50 %), due to the volatility of *n*‐heptane, no starting material was found in the decarboxylation of octanoic acid, indicating complete conversion. According to literature, *Cv*FAP enabled a fourfold higher product formation from octanoic acid compared to palmitic acid under optimized pH values and substrate concentrations. This improved activity was shown to be due to an autocatalytic effect of the formed alkane, which can fill the empty space in the substrate binding pocket, facilitating the octanoic acid decarboxylation.^[57]^ In this context, adding short alkanes as decoy molecules to *Cv*FAP has been used to unlock photodecarboxylation reactions of even smaller carboxylic acids.^[58]^ Based on the fact that palmitic acid (**1**) showed better binding in *CO*FAP (entry 11 in Figure 3) in the docking experiments than octanoic acid (**2**), a similar effect might be the reason for the good activity *in vitro*, although, in this case identical reaction conditions are leading to complete substrate conversion for both substrates. Further substrate‐specific optimization of the reaction conditions and especially the pH might therefore allow to even increase the activity of the enzyme. The decarboxylation of hexanoic acid was attempted with *CO*FAP as well, but no product formation was detected.

## Conclusions

Three different sets of protein structures were analyzed for their cavities and matched against four related template cavities on the Catalophore™ platform. The top cavity matches were assessed based on top matching scores and visual inspection of matching scenes. The resulting 30 cavities were used for docking experiments with 25 ligands. The subsequently obtained combined scores considering binding energy and the distance of the ligand's carboxylate group to FAD, led to the selection of the ten final candidates. Nine out of these enzymes were expressed in soluble form. Five substrates were chosen for photodecarboxylation activity testing of the expressed enzymes. Among the five BLAST candidates (Table 1, entries 1–5), decarboxylation of palmitic acid to pentadecane was observed for four. Note, that screening further reaction conditions, substrates or illumination at different wave lengths might still lead to photo‐promiscuous behaviour of the other tested enzymes.[^27^, ^59^] The GMC oxidoreductase from *Coccomyxa* sp. Obi (*CO*FAP) produces the highest amount of the decarboxylation product. Notably, COFAP displayed high activity over a broad pH range (pH 6.5–9.5) and was shown to decarboxylate octanoic acid with complete substrate conversion, while also exhibiting a high affinity for long‐chain fatty acids. These features may prove beneficial for the application of photodecarboxylases in cascade reactions converting mixtures of fatty acids of different lengths, as the accepted pH‐range allows tailoring the reaction conditions according to the other catalysts requirements. In future, the photostability of COFAP needs to be systematically investigated under different illumination conditions[^27^, ^56^, ^59^] and in the presence and absence of stabilizing additives.[^20^, ^21^, ^29^]

Overall, all enzymes that showed the anticipated decarboxylation activity shared a high sequence identity with *Cv*FAP and were among the results of a sequence‐based BLAST search (Table 1, entries 2–5), while more distant candidates (PDB candidates, Table 1, entries 6–10) were not active. However, the applied strategy using multiple sources of enzyme structures allowed the identification of new enzymes with fatty acid photodecarboxylation activity that might not have been discovered using other bioinformatic methods.

## Supporting Information Summary

The authors have cited additional references within the Supporting Information.[^9^, ^38^, ^39^, ^46^, ^48^, ^52^, ^54^, ^55^, ^56^, ^60^, ^61^, ^62^, ^63^, ^64^, ^65^, ^66^, ^67^, ^68^, ^69^, ^70^]

## Conflict of Interests

The authors declare no conflict of interest.

1

## Supporting information

As a service to our authors and readers, this journal provides supporting information supplied by the authors. Such materials are peer reviewed and may be re‐organized for online delivery, but are not copy‐edited or typeset. Technical support issues arising from supporting information (other than missing files) should be addressed to the authors.

Supporting Information

## Data Availability

The data that support the findings of this study are available in the supplementary material of this article.
