# Supplementary material for: Cavity‐Based Discovery of New Fatty Acid Photodecarboxylases
Source: Chembiochem. 2024 Nov 9;25(24):e202400631. doi: 10.1002/cbic.202400631 (PMC11664916; doi:10.1002/cbic.202400631)
Supplement: Supplementary file 1 — Supporting Information [file CBIC-25-e202400631-s001.pdf]

ChemBioChem

Supporting Information

Cavity-Based Discovery of New Fatty Acid Photodecarboxylases

Stefan Simić, Marco Cespugli, Michael C. Hetmann, Ursula Kahler, Valentina Jurkaš, Marikagiusy Di Giacomo, Maria E. Russo, Antonio Marzocchella, Christian C. Gruber, Bettina M. Nestl, Christoph K. Winkler,* and Wolfgang Kroutil*

Supporting Information:

Cavity-Based Discovery of New Fatty Acid Photodecarboxylases

Stefan Simić, Marco Cespugli, Michael C. Hetmann, Ursula Kahler, Valentina Jurkaš, Marikagiusy Di Giacomo, Maria E. Russo, Antonio Marzocchella, Christian C. Gruber, Bettina M. Nestl, Christoph K. Winkler,* Wolfgang Kroutil*

E-mail: christoph.winkler@uni-graz.at and wolfgang.kroutil@uni-graz.at

Table of Contents

Table of Contents 1

1 Experimental Section 2

2 Gene constructs 10

3 Supplementary tables 15

4 Supplementary figures 20

5 Calibration of fatty acids and alkanes 57

6 References 59

1 Experimental Section

1.1 General

Chemicals and reagents. Unless stated otherwise, solvents, reagents and all media components (yeast extract, tryptone, glycerol) were obtained from commercial sources and used without further purification.

Media preparation. The terrific broth (TB) medium for cell cultivation was prepared by dissolving yeast extract (24 g·L^{-1}), tryptone (12 g·L^{-1}), and glycerol (4 mL·L^{-1}) in deionized water (900 mL·L^{-1} of final medium) in one flask (TB base), and KH_2PO_4 (23.1 g·L^{-1} of buffer) and K_2HPO_4 (125.4 g·L^{-1} of buffer) in deionized water (10% v/v of final medium volume) in another (10×KPi solution). The two medium components were autoclaved separately and combined prior to inoculation with the overnight culture. The Lysogeny broth (LB) medium was prepared by dissolving yeast extract (5 g·L^{-1}), tryptone (10 g·L^{-1}) and sodium chloride (5 g·L^{-1}) in deionized water and autoclaving overnight. The LB-agar medium had the same composition as the LB medium, with the addition of agar (15 g·L^{-1}). After autoclaving the medium was allowed to cool down until it can be handled and kanamycin (50 μg·mL^{-1} final concentration) was added before casting the gel into Petri dishes.

Instrumentation. All photodecarboxylation reactions were run in a custom-built photoreactor, fitted with commercial LEDs, unless stated otherwise. For GC-FID analysis, an Agilent Technologies 7890A GC(FID) system, equipped with an HP-5 (30 m × 0.25 mm × 0.25 μm) column was used. For GC-FID analysis of decarboxylation mixtures starting from dodecanoic, tetradecanoic, palmitic and octadecanoic acid were analyzed using the ACHIRAL method (total runtime 20.5 min; 100 °C for 0.5 min, then 10 °C/min to 300 °C; injection volume 5 μL). GC-FID analysis of mixtures starting from decanoic acid were analyzed by the ACHIRAL_lowboilers method (total runtime 17 min; 40 °C for 2 min; 10 °C/min to 180 °C; 1 min hold at 180 °C; injection volume 5 μL). GC-FID analysis of mixtures starting from octanoic acid were analyzed by the OCTANOIC_1 method (total runtime 22 min; 40 °C for 2 min; 10 °C/min to 80 °C; 3 min hold at 80 °C; 10 °C/min to 200 °C; 1 min hold at 200 °C injection volume 5 μL) GC-MS measurements were carried out on a 7890A GC System (Agilent Technologies, Santa Clara, CA, USA), equipped with a5975C mass selective detector and an HP-5MS column (5% phenylmethylsiloxane, 30 m x 0.20 mm x 0.25 μm, J&W Scientific, Agilent Technologies) using He as the carrier gas. Injector temperature: 250°C; split ratio: 90:1; Injection volume: 1 μL; Flow rate: 0.7 mL/min; Temperature program: 100 °C for 0.5 min, then 10 °C/min to 300 °C (mixtures containing ethylbenzene were analyzed with an altered temperature program: 40 °C for 2 min, then 10 °C/min to 300 °C). HPLC–MS analysis was carried out on an Agilent 1260 Infinity HPLC system equipped with a Phenomenex Luna C18(2) column (dimensions: 250 mm × 4.6 mm; stationary phase: fully porous silica, C18 with TMS

end-capping; particle size: 5 µm; pore size: 100 Å) and coupled to an Agilent 6120 single-quadrupole MS detector, using water and acetonitrile, each containing 0.1% (v/v) of formic acid, as eluents. Cell lysis was performed with the Branson 450 Digital Sonifier using 2 min 30 s total pulsation time, 2 s pulses, and 4 s of pause, at 30% amplitude. OD_{600} values were measured on an Eppendorf BioPhotometer plus spectrophotometer. Lyophilization was performed on a Martin Christ Freeze Dryer ALPHA 1-4. Rough enzyme amounts in lyophilized cell lysates were estimated by SDS-PAGE densitometry, using the Syngene NuGenius Gel Documentation System and the corresponding Syngene GeneTools software.

1.2 General microbiological and biochemical methods

The SDS-PAGE analyses are performed as follows. Samples (purified enzyme, cell lysate, purification flow-through, cell pellet) are dissolved in urea (6 M in H_2O) and the protein concentration is first determined by means of a Bradford assay. The protein samples (10 µL) are mixed with the Bradford reagent (490 µL of a 1:5 v/v dilution of commercial reagent and water). The samples are diluted if necessary. The final protein solutions (10 µL) are mixed with the equal volume of a Laemmli buffer (2x concentrate). The obtained mixtures are heated at 95 °C for 5 min and spun down. The samples are loaded onto a 10% Bis-Tris gel, along with the PageRuler™ marker (5 µL). The electrophoresis was run at 140 V, using a Tris/MOPS buffer. The gels are stained using a Coomassie Blue solution. The transformation of *E. coli* BL21(DE3) competent cells was performed as follows. All plasmids were dissolved in autoclaved distilled water (50 µL, 100 ng·µL^{-1} final concentration). Competent cells were thawed on ice and autoclaved 1.5 mL Eppendorf tubes were precooled on ice as well. *E. coli* BL21(DE3) cells (100 µL) and corresponding plasmid (3 µL, 100 ng·µL^{-1}) were mixed inside the clean bench and the resulting mixtures were left on ice for 30 min. The LB-agar plates containing kanamycin (50 µg/mL) were warmed up in a 30 °C incubator. The cell/plasmid mixtures were heat shocked by placing the tubes into a benchtop shaker a 42 °C for 10 s. The mixtures were put back on ice. Commercial SOC medium (250 µL) was added to each sample inside the clean bench and the mixtures were shaken at 37 °C, 350 rpm, for 1 h. The resulting cultures (250 µL) were plated onto the agar (kanamycin) plates and incubated at 30 °C overnight. Two colonies were picked from each plate, overnight cultures were grown in LB medium and cryostocks were prepared (15% v/v glycerol).

1.3 Bioinformatic methods

Generating template structures. The Y466A variants of wild type *Cv*FAP (PDB ID: 6YRU) and *Cv*FAP R451K (PDB ID: 6YS1) were generated by homology modelling, using PDB structures (6YRU, 6YS1) as templates.[1] The corresponding template structures for the homology modelling were created in YASARA by deleting carbon atoms from the octadecanoic acid until octanoic acid was obtained and performing an energy minimization. The obtained

structures were uploaded onto the Catalophore™ platform under the “Darwin’s playground” tab.

Creation of homology models. Sequences obtained from PDB entries 6YRU and 6YS1 were changed at position 466 of *Cv*FAP to alanine and uploaded to the platform along with template structures, which were prepared as outlined previously. The applied modelling settings are outlined below.

PSI-BLAST iterations:	6
E-value (PSI-BLAST):	0.5
Templates:	1
Alignments:	5
Oligomerization:	4
Terminal loop size:	10
PDB-Redo:	No
Accuracy:	fast
Sample loops:	50
Delete residues:	None
S-based profiles:	0

The creation of remaining homology models (e.g., from BLAST search sequences) was performed under the same modelling settings. *Cv*FAP (PDB ID: 6YRU) was used as a template for homology modelling of BLAST search sequences.

Cavity procreation. The computational workflow for the cavity calculation is as follows.[2] A protein structure is required as starting point. Within this study, experimental crystal structures, and homology models served as input. The initial step of the workflow is the preparation step, where the input structures are refined for the following cavity calculation by detection and addition of missing residues (in case large portions of the protein are missing *via* homology modelling; otherwise *via* loop modelling) and hydrogens and by handling alternate conformations. In the next step, cavities in the protein are detected using the LIGSITE algorithm.[3] LIGSITE identifies potential ligand binding sites on the surface of proteins by searching for points that are inaccessible to solvent on a cartesian grid along a line on the axes. Based on a cut-off value, grid points are identified as cavity points, if they are sufficiently surrounded by protein. This results in a point cloud that represents the cavity’s size and volume. Next, a set of 19 properties is assigned to each point in the cavity point cloud using AutoGrid4.[4] This results in a set of cavity property point clouds representing the individual physico-chemical properties of the cavity. In a next step, these point clouds can be compared and aligned with cavity point clouds from other proteins based on their individual physico-

chemical properties, allowing for the identification of proteins. In this study, the cavity procreation was performed from uploaded structures (either PDB structures or homology models) at pH 7 in all cases apart from *Cv*FAP, where it was performed at pH 8.5. The minimal cavity volume was set to 50 $Å^3$ and the maximal volume to 500 $Å^3$. FMN and FAD residues were retained as part of the structure.

Cavity matching. In the cavity matching a set of two cavity point-clouds are matched (compared).[2] For this, the point clouds are first superimposed and the superposition is optimized. The differences between target and template cavity point-clouds are calculated by comparing the closest grid points. This results in a "property-score" for each pair of point clouds, which is used to represent the similarity of the two compared point-clouds. The individual property scores of the chosen properties that are being matched are combined into a "total score" by putting different weighs onto them. The "iterative-closest-point algorithm" is performed on this to optimize the score leading to the final value which is ranked against the other cavity pairs.[5] In this study the ICP (iterative closest point) iterations were set to 100 and timeout for the matching algorithm to 300 s. The influence of the different properties is defined by setting property weights. The property weights of cavity shape in the overall score were set to 0.01, desolvation point-cloud to 0.1, electrostatics point-cloud to 0.1, while the weight of remaining property scores was set to 1.

Docking experiments. VINA was used as the docking algorithm (AMBER03 force field). The docking box extension around cavity was set to 5 Å and clustering RMSD to 3 Å. Ligand structures were generated from SMILES strings and cleaned up in YASARA by setting the pH to 7.

Multiple sequence alignment. Multiple sequence alignment of BLAST search candidates was performed using Clustal Omega (https://www.ebi.ac.uk/Tools/msa/clustalo/) with default parameters. The analysis of the output file was performed in Jalview.

1.4 Expression of enzyme candidates

Starting expression conditions. Initially, all ten enzyme candidates were expressed under identical conditions, as follows. For overnight cultures, LB medium (10 mL, 50 $\mu g \cdot mL^{-1}$ kanamycin) was inoculated with single colonies of the corresponding recombinant strain and shaken overnight at 30 °C, 120 rpm. The main cultures were prepared in 2 L baffled flasks by inoculating the autoclaved LB medium (600 mL, 50 $\mu g \cdot mL^{-1}$ kanamycin) with corresponding overnight cultures (6 mL, 1% v/v) and incubating at 37 °C, 120 rpm, until the OD_{600} of 0.6-0.8. The flasks were kept at 4 °C for 1 h before induction with IPTG (600 µL, 1 M). The induction was allowed to proceed for 20 h at 20 °C and 120 rpm, in the dark (flasks were covered with aluminium foil). Cells were harvested by centrifuging (8000 rpm, 10 min) at 4 °C. The harvested cells were resuspended in Tris·HCl buffer (50 mM, pH 8, 100 mM NaCl, 30 mL per sample)

and sonicated once (30% A, 2 s ON, 4 s OFF, 2 min 30 s total ON time). The disrupted cells were centrifuged (17000 rpm, 20 min) at 4 °C. The supernatants were shock-frozen and lyophilized overnight. The entire process upon induction was carried out in absence of light.

Scale-up of *CO*FAP expression. Overnight cultures were prepared by charging three Sarstedt tubes (50 mL) with TB medium (3 × 15 mL), adding kanamycin (15 µL per tube, 50 mg·mL^{-1} stock), and inoculating with the glycerol stock containing *E. coli* expressing COFAP (5 µL per tube). The resulting cultures were shaken overnight at 30 °C, 120 rpm. The main cultures were prepared in five 2 L baffled flasks by inoculating the autoclaved LB medium (600 mL per flask, 50 µg·mL^{-1} kanamycin) with corresponding overnight cultures (6 mL per flask, 1% v/v) and incubating at 37 °C, 120 rpm, until the OD_{600} of 0.6-0.8. The procedure continued further as described previously.

1.5 Colorimetric activity assays

D-amino acid oxidases. The activity of D-amino oxidases from *Homo sapiens* (2E82) and *Sus scrofa* (1DDO) was tested using a modified assay reported previously.[6] The volumes were adapted to fit 96-well microtiter plates. The oxidation of D-alanine to pyruvate, coupled to the reaction with 2,4-dinitrophenylhydrazine (2,4-DNP) was used and the product formation was measured at 445 nm on a plate reader. The average rate after 10 min of reaction was used as an activity indicator. A standard series of pyruvic acid in sodium pyrophosphate buffer (75 mM, pH 8.5) was prepared in five concentrations (0.05, 0.075, 0.1, 0.25 and 0.5 mM). For the activity testing of the enzymes, 25 mM D-alanine in the sodium pyrophosphate buffer (75 mM, pH 8.5) was used. Additionally, 2,4-DNP (1 mM in 1 M HCl) and NaOH (0.6 M in H_2O) were prepared. Each enzyme sample (10 µL, 10 mg·mL^{-1}) dissolved in sodium pyrophosphate buffer (75 mM, pH 8.5) was mixed with D-alanine (25 mM, 50 µL) or with one of the standard series solutions. The mixture was incubated for 10 min at 25 °C. Then, 2,4-DNP (30 µL, 1 mM in 1 M HCl) and NaOH (210 µL, 0.6 M) were added and the samples were incubated for 5 min at 25 °C. Absorbance was measured at 445 nm in endpoint mode after 10 s of shaking (SpectraMax M2 plate reader). Blank measurements were performed as described above by exchanging the cell lysate solution with buffer.

L-lactate oxidase. The activity assay for the L-lactate oxidase from *Aerococcus viridans* was performed as described in a literature report.[7]

Cholesterol oxidases. An assay procedure (Assay Procedure for Cholesterol Oxidase, Merck KGaA Darmstadt, Germany) was used with a modification in the amount of horseradish peroxidase. The cholesterol solution was prepared as follows. To Triton X-100 (50 µL), cholesterol (5 mg) was added and the mixtures was heated at 95 ˚C until cholesterol dissolved. Then, distilled water (900 µL) was added to the hot solution of cholesterol in Triton X-100. The mixture was heated at 95 ˚C for another 60 s. The solution was cloudy and it was cooled under running water, at which point it cleared. Next, sodium cholate (40 mg) was added and dissolved

at room temperature. The solution was filled to 1 mL with water. Furthermore, solutions of 4-aminoantipyrine (4-AAP, 1.76 % w/v in water), phenol (6% w/v in water), horseradish peroxidase (3 mg·mL^{-1} in KPi buffer, 100 mM, pH 7) and enzyme diluent (20 mM KPi buffer, pH 7.0, containing 0.2% bovine serum albumin) were prepared. The working solution was prepared by mixing the buffer (KPi buffer, 100 mM, pH 7, 20.4 mL), the cholesterol solution (1.6 mL), the 4-AAP solution (0.4 mL) and the horseradish peroxidase solution (0.8 mL). The working solution (1.16 mL) was pipetted into a 1.5 mL microcentrifuge tube and equilibrated at 37 °C for 3 minutes in the benchtop shaker. The phenol solution (40 µL) was then added and the mixture incubated for another 2 min. Finally, the enzyme solution was added (40 µL, 25 mg·mL^{-1} lyophilized lysate for 2I0K and 2.5 mg·mL^{-1} for 3JS8), the mixture was rapidly transferred to a cuvette (d = 1 cm) and the absorbance was followed at 500 nm and 37 °C for 3 min.

1.6 Photodecarboxylation activity

The samples for the assessment of photodecarboxylation activity of hypothetical protein candidates were prepared in 1.5 mL glass vials as follows. Palmitic acid (100 µL, 100 mM) in DMSO was combined with KPi buffer (400 µL, 100 mM, pH 8) and lyophilized cell lysate (500 µL, 20 mg·mL^{-1}) in KPi buffer (100 mM, pH 8). Reaction mixtures containing all other substrates were prepared by mixing the appropriate substrate solution (500 µL, 20 mM) in KPi buffer (100 mM, pH 8) and lyophilized cell lysate (500 µL, 20 mg·mL^{-1}) in KPi buffer (100 mM, pH 8).

The decarboxylation of DL-tryptophan, DL-phenylalanine and *all-rac*-3-phenylserine were performed under inert conditions. For this purpose, KPi (100 mM, pH 8) buffer was purged with nitrogen for 1 h and the reaction mixtures were prepared inside the glove box. Cell lysates of the five hypothetical protein candidates were weighed in the dark and dissolved in the glove box. The substrate stock solutions were likewise prepared in the glove box. DL-tryptophan (20 mM) in KPi buffer (100 mM, pH 8) was sonicated until a uniform suspension was formed and used as such for the preparation of reaction mixtures. Palmitic acid and 3-phenylpropanoic acid were prepared under an aerobic atmosphere. All samples were prepared in duplicates and illuminated at 455 nm (36 µmolL^{-1}s^{-1}), 500 rpm, 25 °C, for 20 h using a custom photoreactor.[8-9] After the elapsed time, the workup of reaction mixtures was different depending on the substrate.

Palmitic acid. The entire volume (1 mL) of all reaction mixtures was transferred to 2 mL microcentrifuge tubes and after the addition of HCl (50 µL, 6 M), extracted with EtOAc (500 µL, 300 µL withdrawn) and centrifuged (14000 rpm, 1 min). Samples were dried over anhydrous Na_2SO_4, centrifuged (14000 rpm, 5 min) and derivatized as described previously (BSTFA/Pyridine), before GC-MS analysis (HP-5 column, ACHIRAL-MSD method).

3-Phenylpropanoic acid. The entire volume (1 mL) of all reaction mixtures was transferred to 2 mL microcentrifuge tubes and after the addition of NaOH (50 µL, 2 M), extracted with EtOAc

(500 µL, 300 µL withdrawn) and centrifuged (14000 rpm, 1 min). Samples were dried over anhydrous Na_2SO_4, centrifuged (14000 rpm, 5 min) and analyzed directly by GC-MS (HP-5 column, ACHIRAL-MSD lowboilers method).

DL-Tryptophan, DL-phenylalanine, *all-rac*-3-phenylserine. For HPLC-MS analysis, aliquots of all samples (200 µL) were mixed with HPLC-grade acetonitrile (200 µL), briefly sonicated in the sonication bath and centrifuged (14000 rpm, 5 min) before analysis (see instrumentation for method details). For GC-MS analysis, aliquots (500 µL) of all reaction mixtures were transferred to 2 mL microcentrifuge tubes and after the addition of NaOH (50 µL, 2 M), extracted with EtOAc (500 µL, 300 µL withdrawn) and centrifuged (14000 rpm, 1 min). Samples were dried over anhydrous Na_2SO_4, centrifuged (14000 rpm, 5 min) and derivatized as described previously (BSTFA/Pyridine), before GC-MS analysis (HP-5 column, ACHIRAL-MSD method). All-rac-3-phenylserine was not analyzed by GC-MS.

1.7 Characterization of fatty-acid photodecarboxylase from *Coccomyxa* sp. Obi (*CO*FAP)

Screening of pH values. The set of buffers (100 mM) prepared for the screening of photodecarboxylation activity of fatty-acid photodecarboxylase from *Coccomyxa* sp. Obi (*CO*FAP) at different pH values is as follows: sodium citrate (pH 5, 5.5), potassium phosphate (pH 6-8), Tris·HCl buffer (pH 8.5-9), Glycine·NaOH (pH 9.5, 10).

Reaction mixtures were prepared by mixing the appropriate buffer (400 µL), lyophilized lysate of *CO*FAP dissolved in the same buffer (500 µL, 20 mg·mL^{-1}) and DMSO (100 µL, 10% v/v final concentration). All samples were prepared in duplicates and illuminated at 455 nm (36 µmol·L^{-1}·s^{-1}, 1% dutycycle), 500 rpm, 25 °C, for 20 h using a custom photoreactor.[8-9] After the elapsed time, reaction mixtures were acidified with $HCl_{(aq)}$ (6 M, 50 µL) and the entire volume was transferred to 2 mL microcentrifuge tubes and extracted with *n*-decanol (5 mM in EtOAc, 2 × 500 µL, 300 µL withdrawn) and centrifuged (14000 rpm, 1 min). Samples were dried over anhydrous Na_2SO_4, centrifuged (14000 rpm, 5 min) and derivatized as described previously (BSTFA/Pyridine) before GC-FID analysis.

Screening of fatty acids. The fatty acids that were used for the experiment were octadecanoic (stearic), hexadecenoic (palmitic), tetradecanoic (myristic), dodecanoic (lauric), decanoic (capric), octanoic (caprylic), oleic acid. Stock solutions (100 mM) of each acid in DMSO were made and the preparation of reaction mixtures, workup and analysis were carried out as described previously. Tris·HCl buffer (100 mM, pH 8.5) was used as the buffer. No co-solvent was used in reaction mixtures containing hexanoic acid. Instead, the stock solution (20 mM) of the substrate in Tris·HCl buffer (100 mM, pH 8.5, 500 µL) was mixed with *CO*FAP (44 mg·mL^{-1} lyophilized cell lysate, 0.22 mg·mL^{-1} *CO*FAP) in the same buffer (500 µL). The workup and

analysis were likewise performed as described previously and calibration was only performed for the substrate.

2 Gene constructs

The shown sequences represent final gene constructs as provided by the supplier, the parts of the construct corresponding to protein sequences are highlighted in grey.

>KAF5834678.1 GMC oxidoreductase-domain-containing protein [*Dunaliella salina*]
ATGGGCAGCAGCCATCATCATCATCATCACAGCAGCGGCCTGGTGCCGCGCGGCAGCCATATGATGCTGCAGGGT
AAAGTGAGCCTGCGTGTTGGTCAGCATGGTCTGCGTCTGGGCAGTCGTAGCGGTATTAGTAAAGTTGCAGTTCGT
CCGCTGCCGGTTCTGCGTGCCACCGGTGAAAAATATGATGCCATTATTGTGGGCGGTGGTACCGCAGGTTGTGTT
CTGGCAGATCGCCTGAGTGCCGATGAAAATAAACGTGTGCTGGTGCTGGAAGCCGGCCCGAAAGGCGATACCTGG
GAAACCTGGGTTCCGGCCGGCATTACCCGTCTGTTCAATCATCCGGTTCTGGATTGGGGCCTGAAAACCGTGAGT
CAGAAACAGCTGACCGAACGTGAAGTGTATCTGGCCCGTGGTAAAGCCCTGGGCGGCAGCAGCTGTACCAATGCC
ACCCTGTATCATCGCGGTACCGCAGCCGATTATGATAGTTGGGGTCTGGAAGGCTGGAAAAGTGATGAACTGCTG
AAATGGTTCAATCAGGCCGAAAACTTCGAAGATGGTCCGGTTAGCCCGTATCATGGTGTTGGCGGTGCAATGCAT
GTTGAACGTCCGCGCTATGATACCCCGATGCATGAAAACTTCTTCAAAAGTTGCGCAAATGCAGGTCTGCCGGCA
AATCCGGACTTCAATGATTGGAGTCGTCCGCAGGCCGGTTATGGTGAATTCCTGGTGAGCCAGAAAAAAGGCCAG
CGTGCCGATGCCAATCGTATGTATCTGAAACCGGCAATGGGCCGCCCGAATCTGGAAGTGGTGACCGAAGCCCGC
ACCACCAAAATTATGTTCGATCGCGCCGCCGGTGTGCTGCCGAAAGCCGTTGGTGTGCAGTTCAGCCAGGGTGGC
GCAAATGAAAGTGCCCAGCTGGCCGAAGGTGGCGAAGTTCTGATGTGCAGTGGCGCAGTTCATAGCCCGCATCTG
CTGCAGCTGAGCGGTATTGGTCCGGCCGCCCAGCTGCAGCAGCTGGGTATTGAAGTTGTTAGCGATCTGCCGGGT
GTTGGTCAGAATCTGCAGGATCATCCGGCAACCCTGGTGGGTTGCCTGACCGCAGAAGAATTCGATGATCTGGCC
GTGACCAGCCAGATCTATAATAAAAAAAGTGAAGTGAAGAAGCGCCGCGTGCTGCAGTATCTGCTGACCAAAACC
GGCCCGCTGGCCACCACCGGTTGCGATCATGGCGCATTCCTGAGTACCACCGGTAGCGGTGATCCGGATCTGCAG
ATGCGCTTCGTTCCGGCCTTCAGCCTGGACCCTGATGGCGTGCAGGCATATATTAAATTCGCAGAACTGAAACGC
CTGGGTCAGAGCTGGCCGTGTGGTGTGACCATTCAGCTGCTGGCCGTTCGTCCGAAAAGTCGTGGTAGCGTTGGT
CTGCGCAGCACCGATCCGTTCGCAAATCCGGCAATTGATCTGAATTATTATAGTGATCCGGAAGGTGCAGATATT
CGTACCCTGCGCGAAGGCATTCGTCTGGCACGTAATATCTTCCGCCAGCAGCCGCTGGCAAGTTATATTAAAGAA
GAAAAATGGCCGGGTCCGCAGGTTCAGGAAGATGCAGATCTGGATGCCTTCATTCGTAGTAGTACCTGTAGCGGC
AATGCACTGGTGGGCACCTGTCGCATGGGTCTGCCGGAAGATCCGAATGCAGTTGTTAGTGCCACCGATCTGAGC
GTGCGCGGCGTTAGCGGTGTGCGCGTTGTGGATAGTAGTGTGATTCCGCGTATTCCGGGCGGCCAGACCGGTGCA
GCAACCGTTATGGTGGCAGAACGTGCCGCCGCAATGCTGACCAGTAAACAGCCGATGGTGCGTGGCAGTCCGGGT
AGTATTGGCGATAAACAGCTGGTGGCAAGCGCCTAACTCGAG

>KAG2498954.1 hypothetical protein HYH03_003144 [*Edaphochlamys debaryana*]
ATGGGCAGCAGCCATCATCATCATCATCACAGCAGCGGCCTGGTGCCGCGCGGCAGCCATATGCTGGGTCAGAAA
CATGCCGCACTGAAAGGCCAGGCCCGCCCGAGAGCAGCCGCACGTAGACCTGTGGTGAGCGTGCGTGCAGCCGCA
GAAAAATATGATTATATTATTGTGGGCGGTGGTACCGCAGGCTGCGTTCTGGCAAATCGCCTGAGCGCCGATGGT
AGCAAAAAAGTGCTGGTGCTGGAAGCAGGCCCGCCGGGTGATGCCCTGGAAGTTGCCGTGCCGGCAGGCATTGCC
CGCCTGTTCGGCCATCCGGTTCTGGATTGGGGTATGAGCAGCGTGACCCAGAAACAGCTGCTGGCACGTGAAGTG
TATCTGGCCCGCGGCCGTATGCTGGGCGGTAGTAGCGGCAGCAATGCAACCCTGTATCATCGCGGTAGCGCCGCA
GATTATGATGCATGGGGCCTGGAAGGTTGGAGCAGCAAAGATGTTCTGGATTGGTTCATTAAAGCCGAAAATTAT
GCCGAAGGCCCGAAACCGTATCATGGCCAGACCGGCCCGATGCATACCGAACAGCCGCGCTATCAGAATCCGCTG
CATGATGAATTCTTCCGCGCCGCCGCCGCAGCAGGCATTAAAGAAAATCCGGACTTCAATGATTGGAGCCATAGC
CAGGATGGCTTCGGTGAATTCCAGGTGTTCCAGAAAAAAGGTGAACGTGCAGATGCCTATCGTACCCATCTGCGC
GATGCCCTGAAACGCGGTAATCTGAAAGTGGTTACCGGCGCCCGTAGTACCAAAGTGGCCACCGAAAGTGGTGCC
GGCGGCCCTCGTGCAGTTGGTGTTGAATATGCAATGAGTCAGTTCGGTGAACGTACCAGTGCACATCTGGCCCCG
GGCGGCGAAGTTCTGCTGACCGCAGGCGCAGTGCATAGTCCGCATCTGCTGATGCTGAGCGGTGTGGGCCCGGCC
GCTACCTTAGCCGAACATGGCATTCCGCTGGTGGCCGATGTGGCCGGTGTTGGTGCCAATCTGCAGGATCATCCG
GCAGCAGTTCTGGCAGTTCGCGCCAAACCGGAATTCGAAAAACTGAGTGTTACCAGCGAAATCTATGATGATAGT
TGTAATATCAAGCTGGGCGCAGTTCTGAATTATCTGATTAATCGTCGCGGTCCGCTGGCAACCACCGGTTGCGAT
CATGGTGCATTCATTCGCACCAGCGCAGCCCATGCACAGCCGGATCTGCAGCTGCGCTTCGTGGCCGGCTGTGCA
CTGGACCCTGATGGTGTTCAGAGTTATAAAGTGTTCGGCGATCTGAAAAAACAGGGCAAACGTTGGCCGGGTGGT

ATTACCATGCAGCTGCTGGCGATTCGTGCAAAAAGTAAAGGTCATGTGGGTCTGAAAGGCAGTGATCCGTTCGCC
AGTCCGGCCATTGATATTAATTACTTCCAGGACCCTGCAGATCTGGCCACCCTGAAAGCCGCACTGAAGATTGCA
CGCGATATTGCCAATCAGGAACCGCTGAAAAAATATCTGGAAGAAGAAACCTTCCCGGGTAGCCGCGCCAGTAGT
GATGAAGCAATTGAAGAATATGTGCGCCGTACCGTGCATAGCGGTAATGCACTGGTTGGCACCTGCGCAATGGGT
GCCGTGGTTGCCGCAGATAGCCTGAAAGTGCATGGTGTTGAAGCACTGCGCGTTATTGATAGCAGCGTGCTGCCG
AGTATTCCGGGTGGTCAGACCGGTGCCCCGACCGTTATGGTGGCCGAACGCGCAGCCCAGGCCCTGTTAGGCGGT
AAACCGCTGACCCCGCGCCGCGAACTGGTTGCTGCCTAACTCGAG

>XP_002948047.1 hypothetical protein VOLCADRAFT_103673 [*Volvox carteri f. nagariensis*]
ATGGGCAGCAGCCATCATCATCATCATCACAGCAGCGGCCTGGTGCCGCGCGGCAGCCATATGCTGCTGGGTCAG
CGCCCGTTCGGCGCACCTGCTAAAGGCGCAATGCCGTGCTGGAAAGCAGCCCGTCATGGCGGCGTTGCAGGTGTT
GCACGCCGCCCTGTTGCAGTTAAAGCCGCAGCCAGCGTGGGCAGCGAAAAATTCGATTATATTCTGGTTGGTGGC
GGCACCGCCGGTTGCGTGCTGGCAAATAAACTGAGCGCCAATGGCAGTAAAAAAGTGCTGGTTCTGGAAGCAGGC
CCGACCGGCGATGCCATGGAAGTGGCAGTGCCGGCAGGCATTGCCCGTCTGTTCGCACATCCGGTGTTCGATTGG
GGTATGAGCAGCCTGACCCAGCAGCAGCTGGTTGCACGTGAAATCTATCTGGCACGTGGTCGCCTGCTGGGCGGC
TCAAGCGGTACCAATGCAACCCTGTATCATCGTGGTACCCCGGCAGATTATGATAGTTGGGGTCTGGAAGGTTGG
ACCAGCAAAGATCTGCTGGATTGGTTCGTGAAAGCCGAATGCTATGGCGATGGTCCGCGTGCATTCCATGGCCAG
AGCGGTAGCATGAATGTGGAACAGCCGCGTTATCAGAATGTGCTGCATGATGAATTCTTCCGCGCAGCAGCCGCA
GCCGGCCTGCCTGCAAATGAAGACTTCAATGATTGGAGTCGTCCGCAGGAAGGCTATGGTGAATTCCAGGTTGCA
CAGAAAAATGGTGAACGCGCAGATACCTATCGTACCTATCTGAAACCGGCCATGGGTCGCGATAATCTGAAAGTT
ATGACCGGTGCCCGTACCACCAAAGTTCATATTGAAAAAGCAGTACCGGTCCGCGTGCCCGCGGTGTGGAATAT
GCAACCCAGCAGTTCGGCGAACGTTATACCGCCGAACTGACCCCGGGTGGTGAAGTGCTGATGTGTACCGGTGCA
GTGCATACCCCGCATCTGCTGATGCTGAGTGGCATTGGTCCGGCACCGACCCTGCTGGAACATGGCCTGGATGTT
ATTAGTAGTCTGCCGGGTGTGGGTGCAAATCTGCAGGATCATCCGGCAGCCGTTCTGGCCGTTCGTGCAAAACCG
GAATTCGAAGGTCTGAGCGTGACCAGTGAAATCTATGATAGCAAATGTAATATCCGCCTGGGCGCCGTGATGAAA
TATCTGTTCGGTCGTCGCGGCCCGCTGGCAACCACCGGTTGTGATCATGGCGCATTCGTGCGTACCAGCGCCAGC
CATAGCCAGCCGGATCTGCAGATGCGCTTCGTGCCGGGCTGCGCACTGGACCCTGATGGCGTGAAAAGTTATATT
GTGTTCGGCGAACTGAAAAAACAGGGTCGTGCATGGCCGGGTGGCATTACCCTGCAGCTGCTGGGCATTCGCGCC
AAAAGCCGTGGCAGCATTGGTCTGAAAGCCGCCGATCCGTTCATTAATCCGGCAATTAATATTAATTACTTCAGC
GATCCGGAAGATCTGGCCACCCTGAAAAATGGTGTTCGTATTGCACGCGAAATTGTGGCCCAGGAACCGCTGCGT
AAATATCTGCTGGAAGAAACCTTCCCGGGTGAACGTGCAAATACCGATAAAGATATTGAAGAATACGTGCGTCGC
ACCGTTCATAGTGGTAATGCACTGGTGGGTACCTGCGCAATGGGTACCACCCCGGCCAGTGGCGCCGTTGTGAGT
AGCGCAGATCTGAAAGTGTTCGGCGTTGATGGCCTGCGTGTTGTGGATGCAAGTGTGCTGCCGCGTATTCCGGGC
GGCCAGACCGGTGCTGCAACCGTTATGGTGGCCGAACGCGCAGCCGCAATGCTGCTGGGTCAAGCCACCATTACC
AGCCGTCGTGAACCGGCAGCAGTTTAACTCGAG

>BDA51473.1 Oxygen-dependent choline dehydrogenase [*Coccomyxa* sp. Obi]
ATGGGCAGCAGCCATCATCATCATCATCACAGCAGCGGCCTGGTGCCGCGCGGCAGCCATATGGCTAGCCAGAGC
GTGTTCCTGGGTACCCGTCCGGCCACCCGTGCACCTGTTCTGCCGATTGGTCGCGGTGCACATGGTAGCGCAGGC
CGTCGTGCCCTGCGCGTTCGTGCAACCCTGAAAAGTGAAAATCCGGCCGCCGAAAAATATGATTATATTCTGGTT
GGCGGTGGCACCGCCGGCTGTGTGCTGGCAAATCGCCTGACCGCAGATGGCACCAAAAAAGTTCTGATGCTGGAA
GCCGGCGGCGTGAATGAAGCAAAAGAAGTTCGTACCCCGGCAGGCCTGCCGCGCTTATTCAAAAGTGGCCTGGAT
TGGAATCTGTATAGCACCCTGCAGCATGCCGCAAATGAACGTAGCATCTATCTGGCCCGTGGCAAACTGCTGGGC
GGTAGTAGTGCAACCAATGCAACCCTGTATCATCGTGGTACCGCCGCAGATTATGATGCATGGGGCGTTCCGGGC
TGGACCAGCCAGGATGCACTGAAATGGTTCATTCAGGCCGAAAATAATTGTCGTGGCATTGAAGATGGCGTTCAT
GGTACCGGTGGCCTGATGCGTGTTGAAAATCCGCGCTATAATAATCCGCTGCATGATGTGTTCTTCCAGGCAGCA
AAACAGGCCGGCATTCCGGAAAATGATAACTTCAATGATTGGCGTCGTAGTCAGGCAGGCTTCGGTGAATTCCAG
GTGACCCATAGTAAAGGTGAACGTGCAGATTGCTTCCGCATGTATCTGGAACCGATTCTGGGTCGCAGCAATCTG
ACCGTTCTGACCGGTGCAAAAACCTTAAAAATTGAAACCGAAAAGGCCGGCGGTGCCGCCGTTATTAGCCGTGGT
GTTACCTTCCAGATGAATAGCCAGGATGGTAGCAAACATAGTGCAGAACTGGCACCGGGCGGTGAAGTTGTTCTG
TGCGCCGGCAGTATTCATAGCCCGCAGATTCTGCAGCTGAGCGGCATTGGTCCGCAGGCACAGCTGCGCGAAAAA
GGCATTCCGGTTGTGGCCGATCTGCCGGGTGTGGGTCAGAATATGCAGGATCATCCGGCCTGCCTGAGCGCATTC
TATCTGAAAGAAAGTGCCGGTGCCATTAGTGTGACCGATGAACTGCTGCATACCAATGGCCGTATTCGCACCCGT

GCCATTCTGAAATATCTGCTGTTCAAAAAAGGTCCGCTGGCCACCACCGGTTGTGATCATGGTGCATTCGTGTGC
ACCGCCGGCCAGAGTGAACCGGATCTGCAGATTCGCTTCGTTCCGGGTCTGGCCCTGGACCCTGATGGCATTGGT
AGTTATACCGCCTTCGGCAAAATGAAAGATCAGAAATGGCCGAGTGGTATTACCTTCCAGCTGCTGGGTGTGCGC
CCGAAAAGTCGTGGCACCGTGAGCCTGCGCAGCGATGATCCGTGGGATGCCCCGAAACTGGATATTGGTTATCTG
ACCGATAAAGAAGGCGCAGATCTGGCCACCCTGCGCAGTGGCATTAAACTGAGTCGCGAAATTGCAGCCCAGCCG
GCCTTCGAACCGTATGTTGCCGATGAACTGCATCCGGGTGCAAGCGCAAGCAGCGATGCCGCAATTGATGCATTC
ATTCGTGATACCGTTCATAGCGGCAATGCAAATGTGGGTACCTGCAGCATGGCAGTTGCCGCAGGTGGCAATGCC
GTGGTGGACCCTAGCCTGCGCGTGTTCGGCGTTCGCGGTCTGCGTGTTGCCGATGCCAGTGTGATTCCGGTTATT
CCGGGCGGTCAGACCGGTGCCGCAACCGTTATGGTTGCAGAACGCGCCGCACAGATTCTGCTGGGTGCCGCCGCA
CAGCAGCCGGCAGCAGCAGTTCCGGCAGCACAGCCGGCCCTGGCTTAACTCGAG

>KAI8103262.1 hypothetical protein M9435_004601 [*Picochlorum* sp. BPE23]

ATGGGCAGCAGCCATCATCATCATCATCACAGCAGCGGCCTGGTGCCGCGCGGCAGCCATATGGGTGGTGGTATT
CTGAAACAGGTGAATCAGAATCAGAGCAAAAGCAGCATTACCCGCAGCCGCCTGCGCAAAACACCTATTAGTACC
GCCGTGCCGCGTGGCGTGAATAGTCGTCATATTGTTAGTGCAGCAGCCAGTCAGATTAATGCAAATCCGACCGAA
GGTAAAAAATTCGATTATATTATCGTGGGCGGTGGTCTGGCAGGCTGTGTTCTGGCCAATCGTCTGAGCGCAGAT
CCGAATCTGAATATTCTGCTGGTTGAAGCCGGCCCGGATAATACCAGTCGTAATACCAAAATTCCGGCCGCCTTC
ACCCGCATCTTCCGCAGCGATCTGGATTGGAATCTGTTCACCGAACGCCAGCAGGCACTGGCCGATCGTCAGGTG
TATCTGCCGCGTGGTAAAGTGCTGGGCGGTAGCAGCAGCACCAATGCAACCCTGTATATGCGCGGTAGCAGCGAT
GATTATGATAGTTGGGGTGTGGATGGTTGGAGTAGCAAAGATGTTCTGGAATGGTTCAAACTGGCAGAAACCAAT
GCAGATATTAAAGATCCGGCAGTTCATGGTACCGATGGCCCGATGCATGTGGAAAATCCGCGCTATAATAACTTC
CTGCATCAGACCTTCTTCGAAGCCGCAAAAGAAATTGGTATTCCGGCCAATCTGGACTTCAATGATTGGCGCCAT
AAACAGGAAGGTTATGGCACCTTCCAGGTGACCCAGGATAAAGGTGTTCGTGCCGATGGTTATCGTGAATATCTG
CGTCCGATTGTTAATCGTAATAATCTGCAGATTCTGACCGGCACCAGTGTGACCAAAGTTCTGTTCGATCAGAAA
AAAGCCATTGGCGTGCAGTTCACCACCGAAGAAGTTAGTACCATGCGCGAACGCATGACCGCAGAACTGAAAGCC
GCCGGTGAAGTTATTATGAGCAGCGGCAGTATTCATACCCCGCATATTCTGCAGCTGAGTGGTGTGGGCGATGAT
GCAGCCCTGCAGGAACTGGGTATTCGCCCGACCGCCAATATTCCGGGCGTTGGCAAAAATCTGCAGGATCAGCCG
GCAGTTCTGGTGGCCAGCCCGCTGAAACGCGAACTGGATGGCAAAAGCCTGAGCGATCATATCTATAATGCCAGC
GGCGGTCTGCGCAAACGTGCCATTCTGGCATATCTGCTGTTCGGTAAAGGTCCGCTGACCAGTACCGGTTGCGAT
CGCGGTGCCATGCTGGCCACCGATGCAGCCGAAGGCGGTGCACCGGATCTGCAGATTCGCTTCGTTCCGGGCATG
GCACTGGACCCTGATGGCGTTAGTACCTATACCCGCTTCGGTAAATTCCAGAAAGAAGGTAAAAAGTGGCCGAGC
GGTGTTACCTTCCAGCTGGTGGCAGCCCGCGCCCATGGTCAGGGTAGCGTGTGTATTAAAAGCGATGATCCGTTC
GAAAGTCCGCTGATTAATAGTGGTTATCTGAATGATCAGGGTGGCAAAGATCTGGCCACCCTGCGCAATGGTATT
CATATTGCCCGCAAAATTGCAAGCAGCAAAGCCATGAGTAGTGTGCTGGATGGTGAACTGTTCCCGGGTGAAGAT
ATTAGCACCGATAATGCCATTGAAGAATATATTCGCAAAAGCATTCACAGTAGTAATGCCCTGGTTGGCACCTGT
CGTATGGGCGCAAATCCGGAAAGCGGTGATGTTGTTGATAAAGATCTGCGCATCTTCGGTCTGACCGGCATTCGT
GTTGTTGATGCCAGTGTGATTCCGAAAATTCCGGGTGGCCAGACCGGCGCCGCAACCGTGATGATTGCCGAAAAA
GCAGCAGCCATGCTGGTTGGTAAACGTGAAAGTTAACTCGAG

>7F1Y_1|Chains A, B|Lactate oxidase|*Aerococcus viridans* (1377)

ATGGGCAGCAGCCATCATCATCATCATCACAGCAGCGGCCTGGTGCCGCGCGGCAGCCATATGGAATACAACGCC
CCGAGCGAAATTAAATATATTGATGTGGTGAACACCTACGATCTGGAAGAAGAAGCAAGCAAAGTTGTGCCGCAT
GGTGGCTTCAATTATATTGCAGGTGCAAGTGGCGATGAATGGACCAAACGTGCAAATGATCGTGCCTGGAAACAT
AAACTGCTGTATCCGCGTCTGGCACAGGATGTGGAAGCACCGGATACCAGCACCGAAATTCTGGGCCATAAAATT
AAAGCACCGTTCATTATGGCACCGATTGCCGCACATGGTCTGGCACATACCACCAAAGAAGCCGGTACCGCACGT
GCCGTTAGCGAATTCGGTACCATTATGAGTATTAGTGCCTATAGCGGTGCAACCTTCGAAGAAATTAGTGAAGGT
CTGAATGGTGGTCCGCGTTGGTTCCAGATCTATATGGCAAAAGATGATCAGCAGAATCGCGATATTCTGGATGAA
GCAAAAAGTGATGGTGCCACCGCAATTATTCTGACCGCCGATAGTACCGTTAGTGGTAATCGCGATCGCGATGTT
AAAAATAAATTCGTGTATCCGTTCGGTATGCCGATTGTTCAGCGTTATCTGCGCGGTACCGCCGAAGGCATGAGT
CTGAATAATATCTATGGCGCCAGTAAACAGAAAATTAGTCCGCGCGATATTGAAGAAATTGCAGGCCATAGCGGT
CTGCCGGTGTTCGTTAAAGGTATTCAGCATCCGGAAGATGCAGATATGGCCATTAAACGTGGCGCAAGTGGTATC
TGGGTGAGTAATCATGGCGCACGCCAGCTGTATGAAGCCCCGGGTAGCTTCGATACCCTGCCGGCCATTGCAGAA
CGCGTGAATAAACGCGTTCCGATTGTGTTCGATAGCGGTGTGCGCCGTGGTGAACATGTGGCCAAAGCCCTGGCC
AGTGGCGCCGATGTTGTTGCCCTGGGTCGTCCGGTGCTGTTCGGCCTGGCCCTGGGTGGTTGGCAGGGTGCATAT

AGTGTGCTGGATTACTTCCAGAAAGATCTGACCCGCGTTATGCAGCTGACCGGCAGTCAGAATGTGGAAGATCTG
AAAGGCCTGGATCTGTTCGATAATCCGTATGGCTATGAATATTAACTCGAG

>3JS8_1|Chain A|Cholesterol oxidase|*Chromobacterium* sp. DS-1 (507619)
ATGGGCAGCAGCCATCATCATCATCATCACAGCAGCGGCCTGGTGCCGCGCGGCAGCCATATGGCTAGCATGACT
GGTGGACAGCAAATGGGTCGCGGATCCACCTGCAGTCAGCCGAATAACTTCCCGGCCGAAATTCCGCTGTATAAA
CAGAGCTTCAAAAATTGGGCCGGTGATATTAAAGTTGATGATGTGTGGACCTGCGCACCGCGCAGTGCCGATGAA
GTGGTTAAAGTTGCCAATTGGGCCAAAGATAATGGTTATAAAGTTCGCGCACGCGGTATGATGCATAATTGGAGT
CCGCTGACCCTGGCCGCCGGCGTTAGTTGCCCGGCAGTGGTTCTGCTGGATACCACCCGTTATCTGACCGCCATG
AGTATTGATGCCAGTGGCCCGGTGGCAAAAGTTACCGCCCAGGCAGGTATTACCATGGAAGCACTGCTGACCGGC
CTGGAAAAAGCCGGCCTGGGTGTTACCGCCGCCCCGGCACCTGGTGATCTGACCCTGGGTGGTGTGCTGGCCATT
AATGGTCATGGTACCGCCATTCCGGCAAAAGGCGAACGCCGCCTGGCCGGTGCAAGCTATGGCAGTATTAGCAAT
CTGGTGCTGAGCCTGACCGCCGTTGTGTATGATAAAGCCAGTGGCGCCTATGCCCTGCGCAAATTCGCCCGCAAT
GATCCGCAGATTGCACCGCTGCTGGCCCATGTGGGTCGCAGTCTGATTGTTGAAGCCACCCTGCAGGCCGCACCG
AATCAGCGCCTGCGCTGCCAGAGTTGGTTCAATATTCCGTATGGCGAAATGTTCGCCGCCGCCGGTAGTGGCGGT
CGTACCTTCGCCAGCTATCTGGATAGCGCAGGTCGCGTTGAAGCAATCTGGTTCCCGTTCACCAGTAATCCGTGG
CTGAAAGTGTGGACCGTTACCCCGAATAAACCGCTGTTCAGCCGCCAGACCGATAAACCGTTCAATTATCCGTTC
AGTGATAATCTGCCGGATGAAGTTACCGATCTGGCAAATAAAATTCTGAGCCTGGGCGATGGTAAACTGACCCCG
GCCTTCGGCAAAGCCCAGTTCGCAGCCGCAAGTGCCGGCCTGGTTGCCACCGCTAGTTGGGATCTGTGGGGCTGG
AGTAAAAATCTGCTGCTGTATGTTAAACCGACCACCCTGCGTGTGACCGCCAATGGTTATGCCGTGCTGACCCGT
CGTGAAAATGTGCAGCGCGTGCTGAATGAATTCGTTACCTTCTATCAGGCACGTGTTCAGGCATATCAGCAGCAG
GGCCGTTATCCGATGAATGGTCCGGTTGAAATTCGCGTTACCGGTCTGGATGATCCGAGTGAAGCAGCACTGAGC
GGCGGTGTGGCCCCGGCATTAAGCGCCATTCGTCCGCGTCCGGATCATCCGGAATGGAATGTTGCCGTGTGGCTG
GATATTCTGACCCTGCCGGGCACCCCGTATGCAAATCAGTTCTATCGCGAAATTGAACAGTGGATTGAAGCCAAC
TTCAATGGTAGTTATGCCGCCGTGCGCCCGGAATGGAGCAAAGGTTGGGGTTATACCGATCAGGCAGCCTGGGCC
GATAGTGCAATGCTGCAGACCACCATTCCGAATGCATTCCGTGCCGGTCAGCCGGCAGCAGCCAATTGGGATGCC
GCAAAAGCCGCCCTGGCAGCATACGATCCGTATCGTCTGTTCAGCAGTCCGCTGCTGGATAGTCTGGGTCTGTAA
CTCGAG

>2I0K_1|Chain A|Oxidoreductase|*Brevibacterium sterolicum* (1702)
ATGGGCAGCAGCCATCATCATCATCATCACAGCAGCGGCCTGGTGCCGCGCGGCAGCCATATGAGCACCGGTCCG
GTGGCACCTCTGCCGACCCCTCCTAACTTCCCGAATGATATTGCACTGTTCCAGCAGGCCTATCAGAATTGGAGC
AAAGAAATTATGCTGGATGCCACCTGGGTGTGTAGCCCGAAAACACCTCAGGATGTTGTTCGCCTGGCAAATTGG
GCCCATGAACATGATTATAAAATTCGTCCGCGTGGCGCCATGGCAGGCTGGACACCTCTGACCGTGGAAAAAGGC
GCAAATGTGGAAAAAGTTATTCTGGCAGATACCATGACCCATCTGAATGGCATTACCGTTAATACCGGTGGCCCG
GTGGCCACCGTTACCGCTGGTGCTGGTGCAAGTATTGAAGCCATTGTTACCGAACTGCAGAAACATGATCTGGGT
TGGGCCAATCTGCCGGCCCCGGGTGTTCTGAGCATTGGTGGTGCCCTGGCAGTGAATGCCCATGGCGCAGCACTG
CCGGCAGTTGGCCAGACCACCCTGCCGGGTCATACCTATGGCAGTCTGAGTAATCTGGTGACCGAACTGACCGCC
GTTGTGTGGAATGGTACCACCTATGCCCTGGAAACCTATCAGCGTAATGATCCGCGCATTACCCCGCTGCTGACC
AATCTGGGTCGTTGCTTCCTGACCAGTGTTACCATGCAGGCCGGCCCGAACTTCCGCCAGCGCTGTCAGAGCTAT
ACCGATATTCCGTGGCGTGAACTGTTCGCACCGAAAGGTGCAGATGGCCGTACCTTCGAAAAATTCGTGGCAGAA
AGTGGTGGTGCCGAAGCCATCTGGTATCCGTTCACCGAAAAACCGTGGATGAAAGTGTGGACCGTGAGTCCGACC
AAACCGGATAGCAGTAATGAAGTTGGTAGTCTGGGCAGCGCAGGTAGCCTGGTGGGCAAACCGCCGCAGGCCCGT
GAAGTGAGCGGTCCTTATAATTATATCTTCAGCGATAATCTGCCGGAACCGATTACCGATATGATTGGTGCCATT
AATGCAGGTAATCCGGGTATTGCACCGCTGTTCGGCCCGGCCATGTATGAAATTACCAAACTGGGCCTGGCAGCA
ACCAATGCAAATGATATCTGGGGTTGGAGCAAAGATGTTCAGTTCTATATTAAAGCAACCACCCTGCGCCTGACC
GAAGGCGGCGGTGCTGTTGTGACCAGTCGCGCCAATATTGCAACCGTGATTAATGACTTCACCGAATGGTTCCAT
GAACGCATTGAATTCTATCGTGCCAAAGGTGAATTCCCGCTGAATGGTCCGGTGGAAATTCGCTGCTGTGGCCTG
GATCAGGCAGCAGATGTGAAAGTTCCGAGCGTGGGTCCGCCGACCATTAGTGCCACCCGCCCGCGTCCGGATCAT
CCGGATTGGGATGTTGCCATCTGGCTGAATGTTCTGGGCGTTCCGGGTACCCCGGGTATGTTCGAATTCTATCGC
GAAATGGAACAGTGGATGCGCAGCCATTATAATAATGATGATGCCACCTTCCGTCCGGAATGGAGCAAAGGTTGG
GCATTCGGTCCGGACCCTTATACCGATAATGATATTGTTACCAATAAGATGCGTGCCACCTATATTGAAGGTGTG
CCGACCACCGAAAATTGGGATACCGCACGTGCACGTTATAATCAGATTGATCCGCATCGCGTGTTCACCAATGGC
TTCATGGATAAACTGCTGCCGTAACTCGAG

>2E82_1|Chains A, B, C, D|D-amino-acid oxidase|*Homo sapiens* (9606)
ATGGGCAGCAGCCATCATCATCATCATCACAGCAGCGGCCTGGTGCCGCGCGGCAGCCATATGCGTGTTGTTGTT
ATTGGCGCCGGTGTGATTGGTCTGAGCACCGCCCTGTGCATTCATGAACGCTATCATAGCGTTCTGCAGCCGCTG
GATATTAAAGTGTATGCCGATCGCTTCACCCCGCTGACCACCACCGATGTTGCAGCAGGCCTGTGGCAGCCGTAT
CTGAGCGATCCGAATAATCCGCAGGAAGCCGATTGGAGCCAGCAGACCTTCGATTATCTGCTGAGTCATGTGCAT
AGCCCGAATGCAGAAAATCTGGGTCTGTTCCTGATTAGCGGCTATAATCTGTTCCATGAAGCAATTCCGGACCCT
AGTTGGAAAGATACCGTTCTGGGCTTCCGCAAACTGACCCCGCGCGAACTGGATATGTTCCCGGATTATGGCTAT
GGCTGGTTCCATACCAGTCTGATTCTGGAAGGTAAAAATTATCTGCAGTGGCTGACCGAACGCCTGACCGAACGT
GGTGTGAAATTCTTCCAGCGCAAAGTGGAAAGCTTCGAAGAAGTTGCCCGTGAAGGCGCCGATGTTATTGTTAAT
TGTACCGGCGTGTGGGCCGGCGCACTGCAGAGAGATCCGCTGCTGCAGCCGGGTCGTGGTCAGATTATGAAAGTT
GATGCACCGTGGATGAAACACTTCATTCTGACCCATGATCCGGAACGTGGCATCTATAATAGTCCGTATATTATT
CCGGGCACCCAGACCGTTACCCTGGGCGGTATCTTCCAGCTGGGTAATTGGAGCGAACTGAATAATATTCAGGAT
CATAATACCATCTGGGAAGGCTGCTGTCGTCTGGAACCGACCCTGAAAAATGCACGCATTATTGGTGAACGCACC
GGCTTCCGCCCGGTGCGTCCTCAAATTCGTCTGGAACGTGAACAGCTGCGCACCGGCCCGAGCAATACCGAAGTT
ATTCATAATTATGGCCATGGTGGCTATGGCCTGACCATTCATTGGGGTTGTGCACTGGAAGCAGCCAAACTGTTC
GGCCGCATTCTGGAAGAAAAAAAACTGAGTCGTATGCCGCCGAGCCATCTGTAACTCGAG

>1DDO_1|Chains A, B, C, D, E, F, G, H|D-AMINO ACID OXIDASE|*Sus scrofa* (9823)
ATGGGCAGCAGCCATCATCATCATCATCACAGCAGCGGCCTGGTGCCGCGCGGCAGCCATATGCGTGTTGTGGTT
ATTGGCGCCGGTGTGATTGGCCTGAGCACCGCCCTGTGCATTCATGAACGTTATCATAGTGTGCTGCAGCCGCTG
GATGTTAAAGTGTATGCCGATCGCTTCACCCCGTTCACCACCACCGATGTTGCAGCCGGCCTGTGGCAGCCGTAT
ACCAGTGAACCGAGCAATCCGCAGGAAGCCAATTGGAATCAGCAGACCTTCAATTATCTGCTGAGCCATATTGGT
AGTCCGAATGCAGCAAATATGGGTCTGACCCCGGTGAGCGGTTATAATCTGTTCCGTGAAGCCGTGCCGGACCCT
TATTGGAAAGATATGGTGCTGGGCTTCCGTAAACTGACCCCGCGTGAACTGGATATGTTCCCGGATTATCGTTAT
GGTTGGTTCAATACCAGCCTGATTCTGGAAGGTCGCAAATATCTGCAGTGGCTGACCGAACGTCTGACCGAACGC
GGTGTTAAATTCTTCCTGCGCAAAGTTGAAAGCTTCGAAGAAGTTGCACGTGGTGGCGCAGATGTGATTATTAAT
TGTACCGGTGTGTGGGCCGGTGTGCTGCAGCCTGATCCGCTGCTGCAGCCGGGCCGTGGTCAGATTATTAAAGTT
GATGCACCGTGGCTGAAAAACTTCATTATTACCCATGATCTGGAACGTGGCATCTATAATAGCCCGTATATTATT
CCGGGCCTGCAGGCAGTTACCCTGGGTGGCACCTTCCAGGTTGGCAATTGGAATGAAATTAATAATATCCAGGAC
CATAACACCATCTGGGAAGGCTGTTGTCGCCTGGAACCGACCCTGAAAGATGCCAAAATTGTGGGCGAATATACC
GGCTTCCGTCCGGTTCGTCCGCAGGTGCGTCTGGAACGTGAACAGCTGCGCTTCGGCAGCAGTAATACCGAAGTG
ATTCATAATTATGGTCATGGCGGTTATGGTCTGACCATTCATTGGGGTTGTGCCCTGGAAGTGGCAAAACTGTTC
GGCAAAGTTCTGGAAGAACGTAATCTGCTGACCATGCCGCCGAGCCATCTGTAACTCGAG

3 Supplementary tables

Table S 1. List of candidates obtained after cavity matching and subsequently used for docking experiments.

Entry	GenBank Protein ID[10]	UniProt ID[11]	PDB ID[12]	Name	Source organism	Cavity UUID
**1**	KAF5834678.1	-	-	GMC oxidoreductase-domain-containing protein	*Dunaliella salina*	3B0ADA92-B5C7-4B93-8C9D-FD0154330093
**2**	PXF43763.1	A0A2V3INX6	-	Alcohol dehydrogenase [acceptor]	*Gracilariopsis chorda*	3FF9B123-F840-4BB1-8CE5-C922C175780A
**3**	KAG2445644.1	A0A836B2S8	-	GMC_OxRdtase_N domain-containing protein	*Chlamydomonas incerta*	6E6DFED4-6BA5-426A-A6AC-4FEBB1220BCC
**4**	KAG2498954.1	A0A836C4N3	-	GMC_OxRdtase_N domain-containing protein	*Edaphochlamys debaryana*	34D1A099-CFCF-4ED9-95CE-3196FF097F02
**5**	XP_002948047.1	D8TNQ4	-	GMC_OxRdtase_N domain-containing protein	*Volvox carteri f. nagariensis*	90B19931-7419-4817-8743-C7F3C2AF32B8
**6**	QDM14404.1	A0A515J4I8	-	Choline dehydrogenase	*Neopyropia yezoensis*	47717605-6A7B-49D4-B23D-FF6D0E978C3F
**7**	QDZ18370.1	A0A5B8MG93	-	Glucose-methanol-choline oxidoreductase	*Chloropicon primus*	A6B83A78-0285-4B7B-BA8E-FFA4BB7A45EA
**8**	GAX83712.1	A0A250XLE8	-	GMC_OxRdtase_N domain-containing protein	*Chlamydomonas eustigma*	E4FB1062-1932-4436-97A0-F2FCF8470C7A
**9**	GBF88787.1	A0A2V0NV16	-	Choline dehydrogenase	*Raphidocelis subcapitata*	E5CCC619-CF3D-4E84-8ED2-63470C3A93E1
**10**	XP_005714951.1	R7Q9C0	-	Probable dehydrogenase	*Chondrus crispus*	E9070CD3-F229-4BFB-BE5C-437D81574537
**11**	BDA51473.1	A0A8J9SI66	-	Oxygen-dependent choline dehydrogenase	*Coccomyxa sp. Obi*	EE9CDD11-82DB-4498-903C-58C95C4CC947
**12**	KAI8103262.1	-	-	Hypothetical protein M9435_004601	*Picochlorum sp. BPE23*	FA340192-6A0E-465F-AFF3-28436CBA8391
**13**	EAW11661.1	A1CFL2	-	Patulin synthase	*Aspergillus clavatus* (strain ATCC 1007)	1894430F-29D7-4D9C-BB53-0CCB2968CC26
**14**	BAD82818.1	Q5NT46	4HA6	Pyridoxine 4-oxidase	*Rhizobium loti*	D6EA73C7-5600-4B74-9A3E-10329D61613B
**15**	EAW97837.1	P14920	3G3E	D-amino-acid oxidase	*Homo sapiens*	0459A1AF-AD68-4D77-A1B8-525D4ACDC317
**16**	EAW97837.1	P14920	3ZNO	D-amino-acid oxidase	*Homo sapiens*	0675F92B-A2EB-4644-8B33-6B52031BF9FA
**17**	BAA09172.1	Q44467	7F1Y	L-lactate oxidase	*Aerococcus viridans (strain ATCC 11563)*	1CE4C19D-55B5-4E71-B0BC-46B364EFABF6
**18**	BAG70948.1	B5MGF8	3JS8	Solvent-stable cholesterol oxidase	*Chromobacterium sp. DS-1*	1DE352E4-2677-4ECD-BD20-C52565DD9D44
**19**	EAW97837.1	P14920	3ZNN	D-amino-acid oxidase	*Homo sapiens*	26A9B84F-E47A-4E1A-BDB2-423866C77829
**20**	EAW59380.1 EAW59378.1 AAA59551.1	P27338	1GOS	Monoamine oxidase B	*Homo sapiens*	3FCE8396-F62B-43BB-AF95-B70514089439
**21**	–	Q7SID9	2I0K	Cholesterol Oxidase (H121A variant)	*Brevibacterium sterolicum*	47BCC093-DCC9-4E1F-B38B-3BD3C94D1769

**22**	EAW97837.1	P14920	2E48	D-amino-acid oxidase	*Homo sapiens*	80A78B25-7586-48AE-BBD5-4ADBBE7B99C1
**23**	EAW97837.1	P14920	2E82	D-amino-acid oxidase	*Homo sapiens*	9F54E552-26C6-4C45-9FC5-D6D0D3773376
**24**	ABG93680.1	Q0SFK6	4BK3	3-hydroxybenzoate 6-hydroxylase	*Rhodococcus jostii* RHA1	AA1D5637-AF39-4721-9CFF-70A66CEE1383
**25**	AAB57640.1	O06647	6EM0	2-hydroxybiphenyl 3-monooxygenase M321A	*Pseudomonas nitroreducens*	AE9BE6FE-E864-4B04-9842-6695C5707B68
**26**	AAC46190.1; AAC69358.1; CCP46638.1	P9WIQ1	4RPK	UDP-Galactopyranose mutase	*Mycobacterium tuberculosis* H37Rv	AEF0F0CC-FF47-42B1-9CD4-820ACA445D1C
**27**	EAW97837.1	P14920	4QFD	D-amino-acid oxidase	*Homo sapiens*	C09FFAF0-EC7A-41DF-985A-88916428347E
**28**	EAW97837.1	P14920	3W4K	D-amino-acid oxidase	*Homo sapiens*	C1DA668D-897E-4ABA-9349-10A4A4E61148
**29**	AAA31025.1	P00371	1DDO	D-amino-acid oxidase from pig kidney	*Sus scrofa*	E1205F24-B393-4D7C-AF6A-104A7741B4E2
**30**	EAW59380.1 EAW59378.1 AAA59551.1	P27338	2C73	Human Monoamine Oxidase B (Y435)	*Homo sapiens*	ED84AC91-8CCF-44E4-B9F4-9D30646173C2

Table S 2. List of top 20 Foldseek candidates as outlined in the output file.

Entry	PDB ID[12]	Enzyme name [Source organism]	Foldseek score
**1**	4HA6	Pyridoxine 4-oxidase - pyridoxamine complex [*Mesorhizobium loti*]	1869
**2**	3T37	Pyridoxine 4-oxidase from *Mesorbium loti* [*Mesorhizobium loti*]	1912
**3**	3LJP	Chain B, Choline oxidase [*Arthrobacter globiformis*]	2112
**4**	3NNE	Choline oxidase S101A mutant [*Arthrobacter globiformis*]	2065
**5**	2JBV	Choline oxidase reveals insights into the catalytic mechanism [*Arthrobacter globiformis*]	2010
**6**	4MJW	Choline Oxidase in Complex with the Reaction Product Glycine Betaine [*Arthrobacter globiformis*]	2055
**7**	3FIM	Aryl-alcohol-oxidase from *Pleurotus eryingii* [*Pleurotus eryngii*]	1878
**8**	5OC1	Aryl-alcohol oxidase from *Pleurotus eryngii* in complex with p-anisic acid [*Pleurotus eryngii*]	1913
**9**	4UDP	5-hydroxymethylfurfural oxidase (HMFO) in the oxidized state [*Methylovorus* sp. MP688]	1758
**10**	6F97	V465T mutant of 5-(Hydroxymethyl)furfural Oxidase (HMFO) [*Methylovorus* sp. MP688]	1740
**11**	4UDQ	5-hydroxymethylfurfural oxidase (HMFO) in the reduced state [*Methylovorus* sp. MP688]	1789
**12**	5ZU2	R554A variant of formate oxidase [*Aspergillus oryzae* RIB40]	1757
**13**	5ZU3	R554K variant of formate oxidase [*Aspergillus oryzae* RIB40]	1751
**14**	4UDR	H467A variant of 5-hydroxymethylfurfural oxidase (HMFO) [*Methylovorus* sp. MP688]	1766
**15**	3Q9T	Formate oxidase [*Aspergillus oryzae* RIB40]	1774
**16**	5HSA	Alcohol Oxidase AOX1 from *Pichia pastoris* [*Komagataella phaffii* CBS 7435]	1764
**17**	6H3G	Chain A, Alcohol oxidase [*Phanerodontia chrysosporium*]	1779
**18**	4H7U	Pyranose dehydrogenase from *Agaricus meleagris*, wildtype [*Leucoagaricus meleagris*]	1951
**19**	6ZE7	Chain B, FAD-dependent oxidoreductase [*Thermochaetoides thermophila* DSM 1495]	1686
**20**	6ZE4	Chain B, FAD-dependent oxidoreductase [*Thermochaetoides thermophila* DSM 1495]	1689

Table S 3. Sum of combined scores from the docking experiments for the best PDB-candidates and the best BLAST candidates.

Enzyme identifier	Enzyme name	Source organism	Expression system	Sum of combined scores (kcal/mol·Å)
KAF5834678.1[a] (*Ds*FAP)	GMC oxidoreductase-domain-containing protein	*Dunaliella salina*	-	31.14
KAG2498954.1[a] (*Ed*FAP)	GMC_OxRdtase_N domain-containing protein	*Edaphochlamys debaryana*	-	31.07
XP_002948047.1[a] (*Vc*FAP)	GMC_OxRdtase_N domain-containing protein	*Volvox carteri f. nagariensis*	-	31.57
BDA51473.1[a] (*CO*FAP)	Oxygen-dependent choline dehydrogenase	*Coccomyxa sp. Obi*	-	32.56
KAI8103262.1[a] (*Pi*FAP)	Hypothetical protein M9435_004601	*Picochlorum sp. BPE23*	-	31.73
7F1Y[b]	L-lactate oxidase	*Aerococcus viridans*	*Escherichia coli*[13-14]	41.73
3JS8[b]	Solvent-stable cholesterol oxidase	*Chromobacterium sp. DS-1*	*Escherichia coli*[15]	40.79
1GOS[b,c]	Monoamine oxidase B	*Homo sapiens*	*Komagataella pastoris,*[16] *Escherichia coli*[17]	43.49
2I0K[b]	Cholesterol Oxidase (H121A variant)	*Brevibacterium sterolicum*	not established	36.49
2E82[b]	D-amino-acid oxidase	*Homo sapiens*	*Escherichia coli*[18]	50.22
1DDO[b]	D-amino-acid oxidase from pig kidney	*Sus scrofa*	*Escherichia coli*[19]	51.32
2C73[b,c]	Human Monoamine Oxidase B (Y435)	*Homo sapiens*	*Komagataella pastoris*[20]	43.11

[a]Identifiers refer to GenBank Protein IDs
[b]Identifiers refer to PDB IDs
[c]This candidate was not evaluated *in vitro* as the only reported working expression system was *Komagataella pastoris*

Table S 4. Specific activities of PDB candidate enzyme preparations as determined by colorimetric assays.

Entry	Enzyme PDB ID	Specific activity of cell lysate ($U\cdot mg^{-1}$)[a]	Assay reference
1	1DDO[f]	0.017 ± 0.001[b]	[6]
2	2E82[f]	0.017 ± 0.001[b]	[6]
3	7F1Y[g]	0.013 ± 0.001[c]	[7]
4	2I0K[e]	0.021 ± 0.001[d]	Assay Procedure for Cholesterol Oxidase (Merck KGaA, Darmstadt)
5	3JS8[e]	0.419 ± 0.005[d]	

[a]1 U = 1 $\mu mol\cdot min^{-1}$, the mass refers to lyophilized cell lysate dry weight.

[b]The oxidation of D-alanine (4.2 mM final concentration) to pyruvic acid, coupled to the reaction with 2,4-dinitrophenylhydrazine in presence of the corresponding cell lysate (0.1 mg dry weight) was recorded at 445 nm. The average rate after 10 min of reaction at 25 °C was taken as a measure of activity.

[c]The oxidation of DL-lactic acid (1.04 mM final concentration) to pyruvic acid, in presence of cell lysate (1 mg dry weight), and a subsequent horseradish-peroxidase-catalyzed reaction of 4-aminoantipyrine and 3,5-dichloro-2-hydroxybenzenesulfonic acid with the generated hydrogen peroxide afforded a colored product, the formation of which was monitored at 515 nm for 3 min. Activity was calculated taking into account that one equivalent of hydrogen peroxide affords two equivalents of colored product.

[d]The oxidation of cholesterol (0.89 mM final concentration) into cholest-4-en-3-one, in presence of cell lysate (1 mg dry weight of 2I0K or 0.1 mg dry weight of 3JS8), and a subsequent horseradish-peroxidase-catalyzed reaction of 4-aminoantipyrine and phenol with the generated hydrogen peroxide afforded a colored product, the formation of which was monitored at 500 nm, at 37 °C, for 3 min. Activity was calculated taking into account that one equivalent of hydrogen peroxide affords two equivalents of colored product.

[e]Experimental procedure is described in the experimental section.

[f]The enzymes were expressed according to literature procedures[7, 18, 21] and sonication was performed as described in the experimental section

[g]The enzyme expression and cell disruption were performed according to a literature procedure.[7]

Table S 5. Estimation of the enzyme amount in cell lysates by SDS PAGE densitometry.

Enzyme	Fraction of total protein in cell lysate (%, w/w)[d]	Target enzyme abundance (%)[e]	Amount of target enzyme in cell lysate (% w/w)[f]
KAG2498954.1 (*Ed*FAP)[a]	12.1	5.6	0.7
KAF5834678.1 (*Ds*FAP)[a]	17.1	ND	ND
KAI8103262.1 (*Pi*FAP)[a]	11.1	5.6	0.6
BDA51473.1 (*CO*FAP)[a]	11.9	8.1	1.0
XP_002948047.1 (*Vc*FAP)[a]	9.6	6.8	0.7
1DDO[b]	15.2	6.5	1.0
2E82[b]	21.8	4.7	1.0
7F1Y[b]	13.8	ND	ND
3JS8[b]	14.8	14.8	2.2
2I0K[b]	20.0	21.1	4.2
BDA51473.1 (*CO*FAP)[a, c]	25.6	8.7	2.2

[a]Enzyme identifiers correspond to GenBank ID numbers

[b]Enzyme identifiers correspond to PDB ID numbers

[c]The enzyme preparation used in characterization of *CO*FAP

[d]Ratio of total protein mass, determined by Bradford assay, and the mass of lyophilized cell lysate

[e]Fraction of target enzyme in the total protein amount, determined by SDS-PAGE gel imaging

[f]Fraction of target enzyme mass in the total mass of lyophilized cell lysate

ND - not determined

4 Supplementary figures

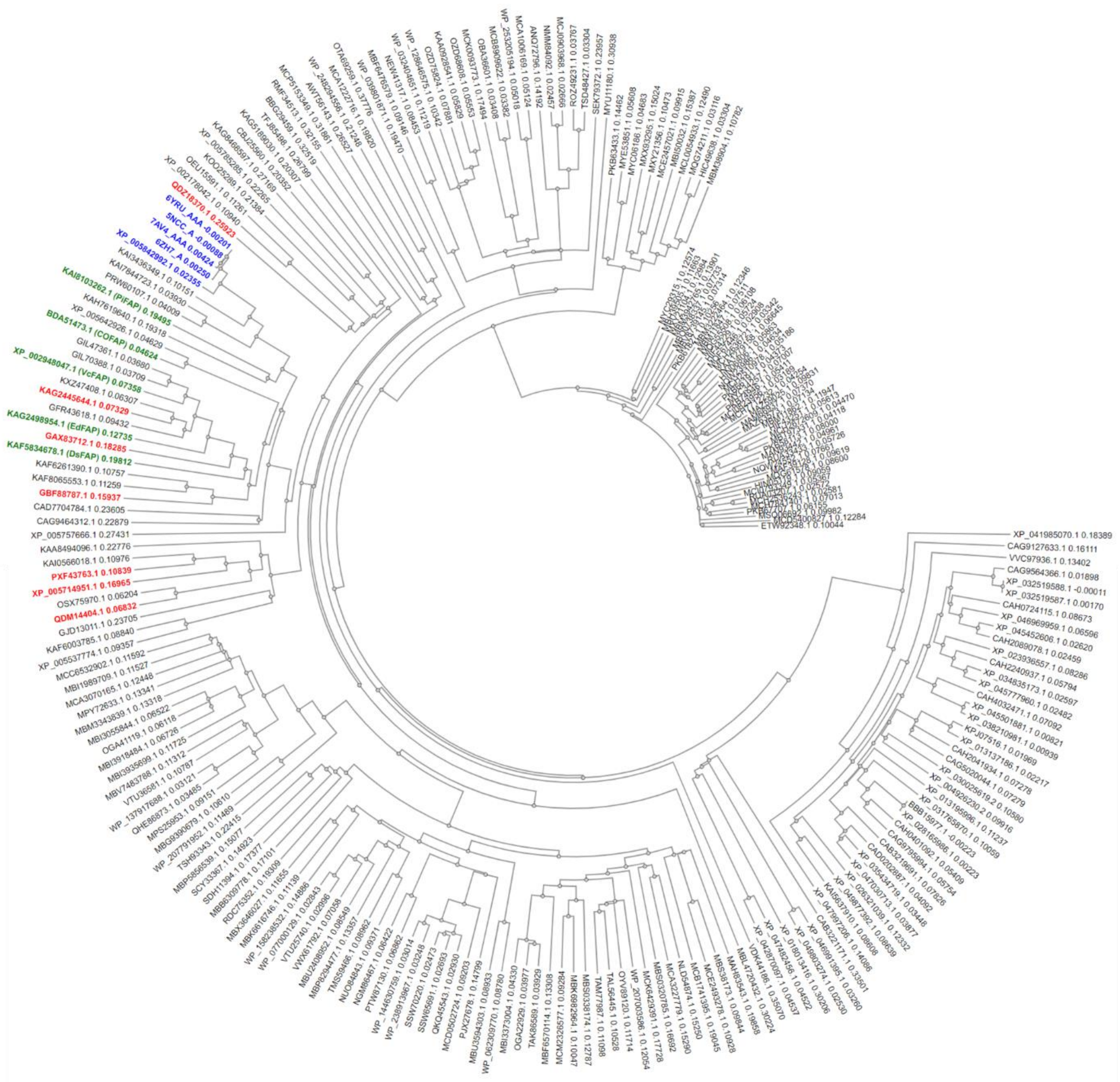

Figure S 1. Phylogenetic tree of the sequences from the BLAST search that were submitted to homology model creation and cavity procreation. The alignment was performed using Clustal Omega and the visualized using the tools from EMBL-EBI.[22-23] A cropped, magnified image of the relevant region is displayed in the next Figure. Blue: *Cv*FAP (the query sequence) and other entries of *Cv*FAP found in the BLAST search. Red and Green: Hits of the cavity matching that were submitted to the docking. Green: Hits of the docking that were evaluated *in vitro*.

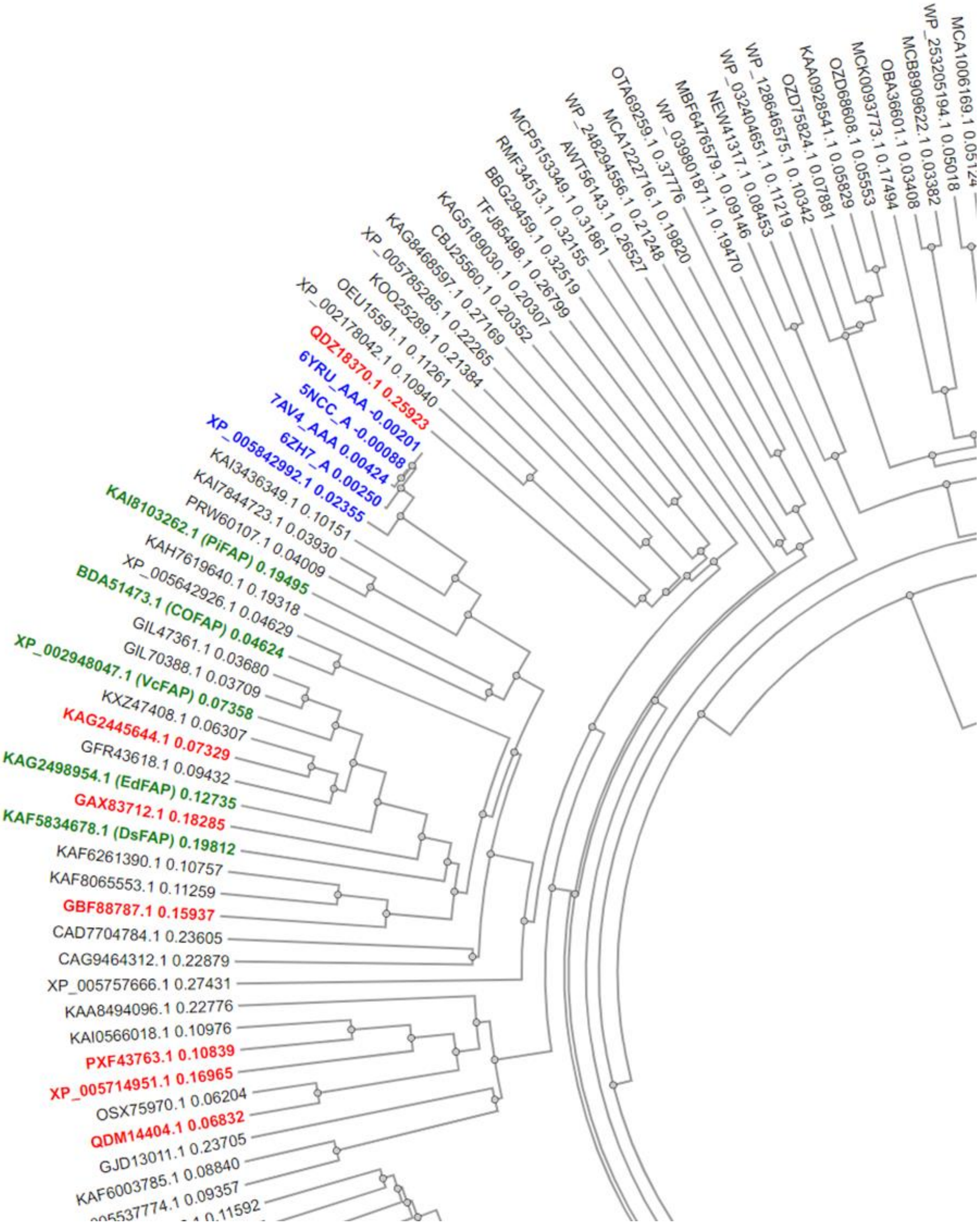

Figure S 2. Croped section of the phylogenetic tree of the previous figure. Blue: CvFAP (the query sequence) and other entries of CvFAP found in the BLAST search. Red and Green: Hits of the cavity matching that were submitted to the docking. Green: Hits of the docking that were evaluated *in vitro*.

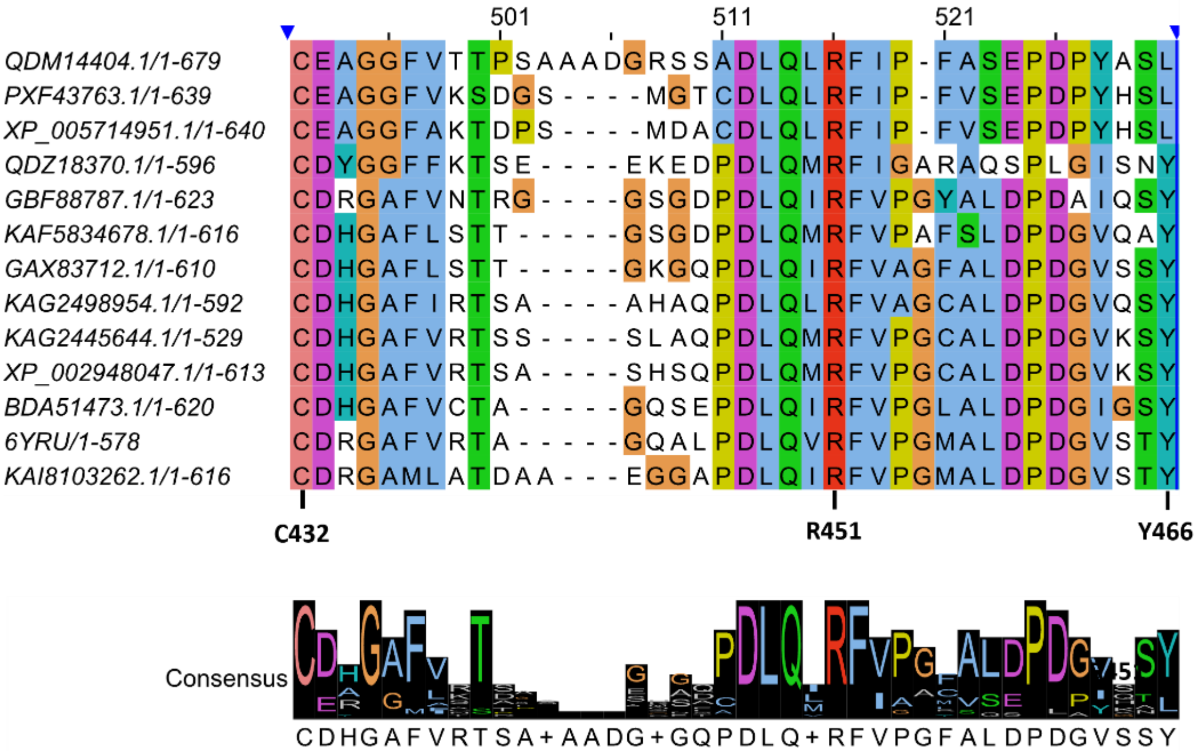

Figure S 3. Multiple sequence alignment of the BLAST search candidate sequences displaying conservation of residues C432, R451 and Y466. The alignment was performed using Clustal Omega[22-23] and the visualization in Jalview.[24]

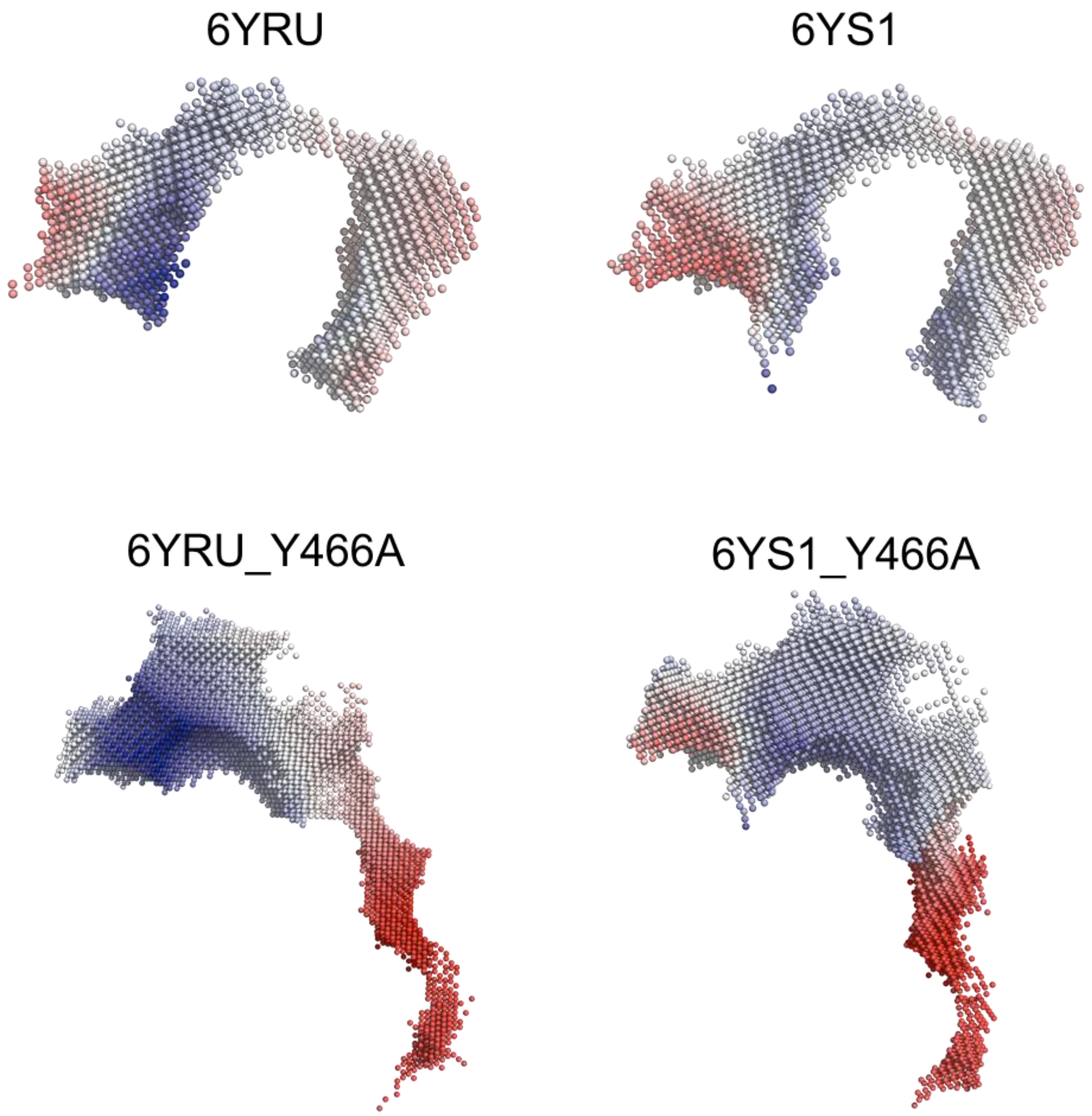

Figure S 4. Template cavities with coloring based on the electrostatic point cloud. All cavities are in the same orientation. The differences in electrostatic potential between the cavities is visible especially on the left-hand side. The effect of the mutations on the cavity's shape and volume is most pronounced on the bottom side. Specifically, when Tyr466, which creates the U-shape in the wild-type (WT) cavities, is mutated to the significantly smaller Ala, more "free space" appears in the cavity, and the cavity point cloud expands. Colored with spectrum b, blue_white_red, minimum=-1, maximum=0.5.

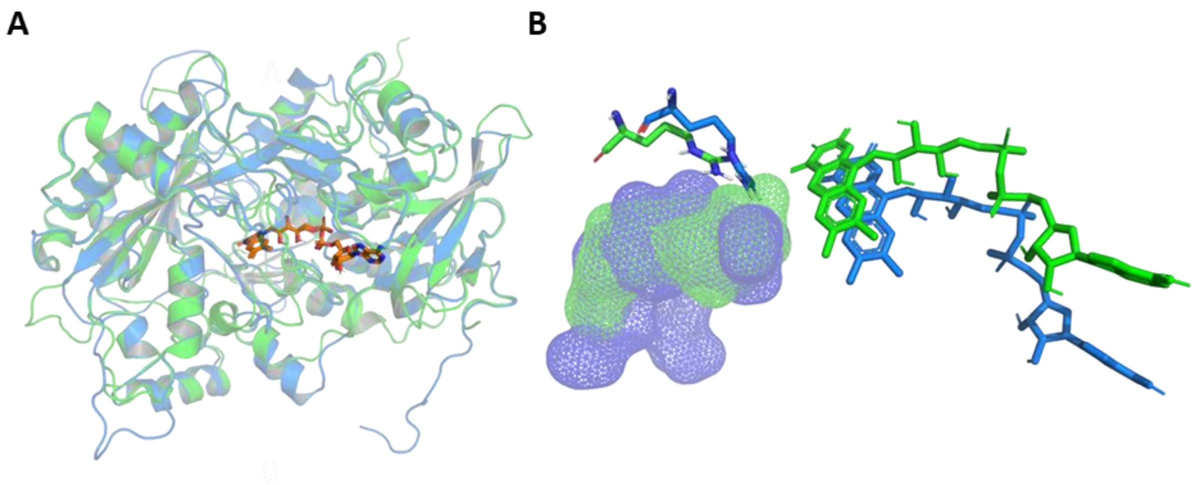

Figure S 5. Matching of the *Cv*FAP cavity 6YRU_C8_min2 (UUID: F2355085-48DA-48A0-8923-D4EC34885F78) and that of patulin synthase (UUID: 1894430F-29D7-4D9C-BB53-0CCB2968CC26). A – The structures of *Cv*FAP (green) and patulin synthase (blue) in the matching scene after alignment (*super* command, PyMOL). B – The matching scene with the omitted protein backbone. The overlapping cavities of *Cv*FAP (green) and patulin synthase (blue) are displayed as wireframes, the point clouds corresponding to cavity properties are not shown. The R451 residue of *Cv*FAP is likewise shown in green and R433 of patulin synthase in blue. The *Cv*FAP cavity occupies 52% of the volume of the patulin synthase cavity and 77% of the *Cv*FAP cavity were overlapped.

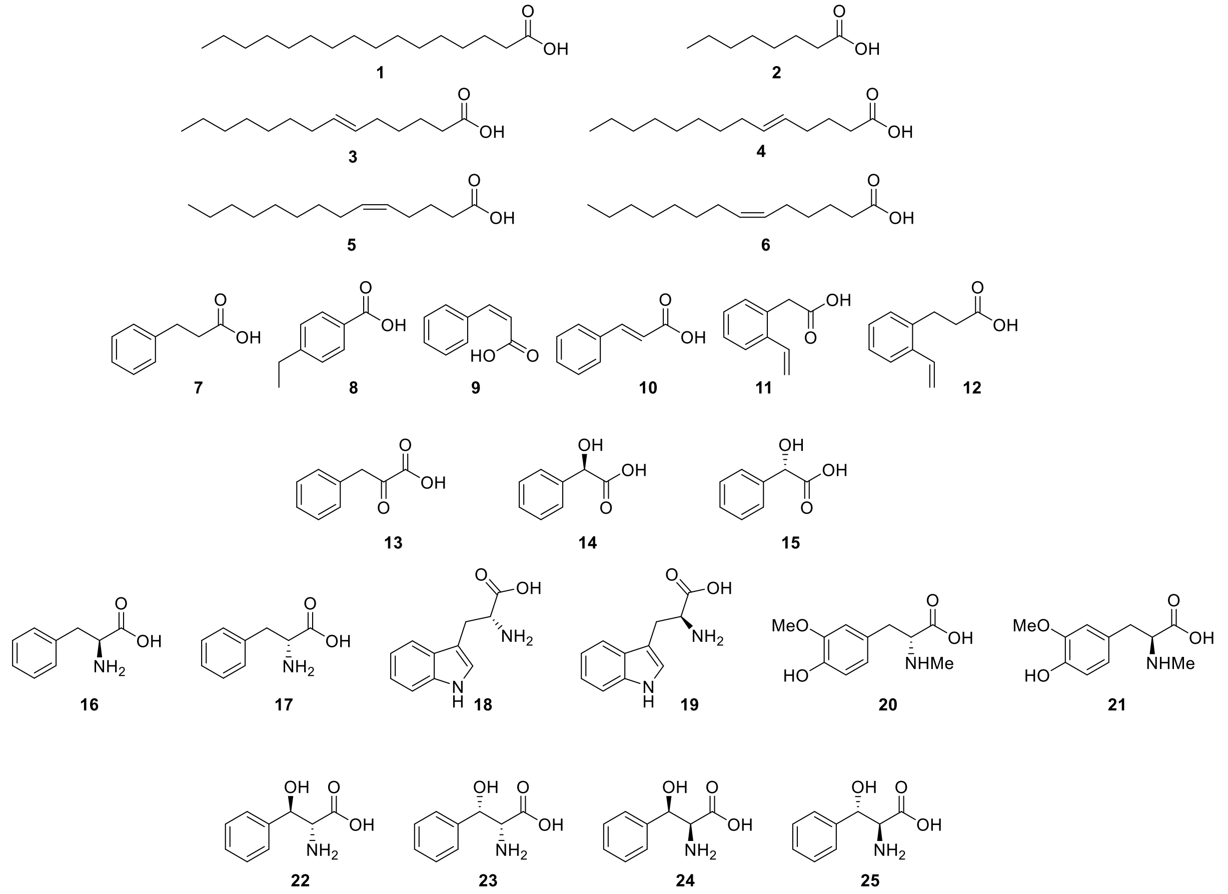

Figure S 6. Structural formulas of molecules used as ligands in the docking with cavity matching candidates.

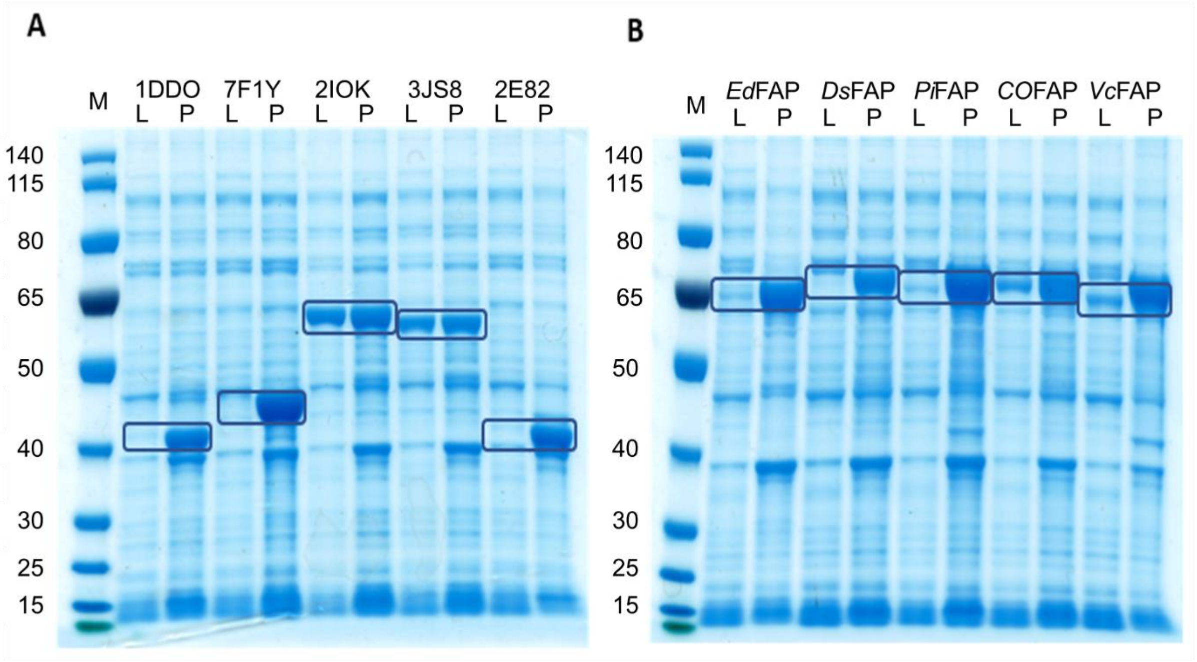

Figure S 7. SDS-PAGE analysis of cell lysates and pellets of expressed Catalophore™ search candidates. A – PDB candidates (the heading symbols represent PDB IDs). B – Hypothetical protein candidates. The heading symbols are the abbreviations from Table 1: *Ds*FAP : KAF5834678.1; *Ed*FAP: KAG2498954.1; *Vc*FAP: XP_002948047.1; *CO*FAP: BDA51473.1; *Pi*FAP: KAI8103262.1; L – cell lysate, P – cell pellet. Expression conditions: Main cultures (LB, 600 mL, 50 µM kanamycin) were inoculated with precultures grown overnight (1% v/v) and shaken at 37 °C, 120 rpm until OD_{600}= 0.6-0.8. Induction was performed in presence of IPTG (1 mM) at 20 °C, 120 rpm, for 20 h, in the dark. The bands corresponding to target enzymes are marked within rectangles.

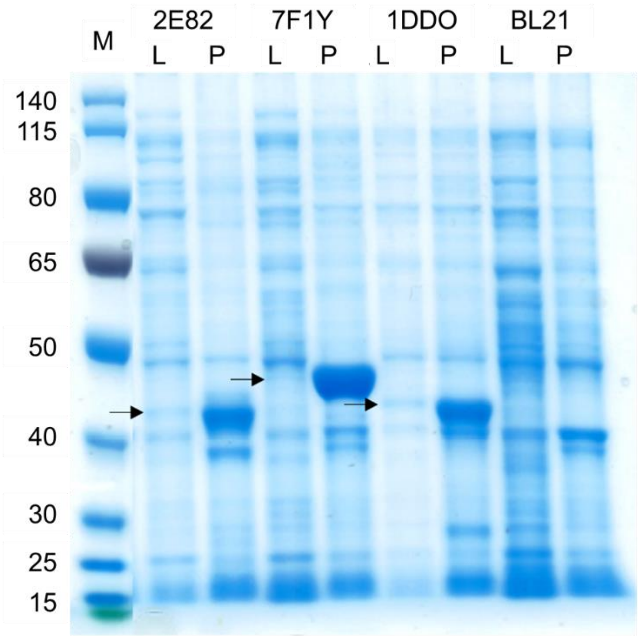

Figure S 8. SDS-PAGE analysis of soluble expression of *Homo sapiens* D-amino acid oxidase (PDB ID: 2E82), *Sus scrofa* D-amino acid oxidase (PDB ID: 1DDO), and *Aerococcus viridans* L-lactate oxidase (PDB ID: 7F1Y) under expression procedures, based on literature reports.[25-27] The heading symbols are: M – marker (PageRuler™ Prestained Protein Ladder), L – lysate, P – pellet. BL21 are empty *E. coli* BL21 cells for comparison. Expression conditions: 2E82: TB medium, supplemented with glucose (0.5% w/v), induction at OD_{600} = 0.6 with IPTG (0.1 mM) at 37°C, 120 rpm, for 24 h, in the dark. 7F1Y: TB medium, induction at OD_{600} = 1 with IPTG (0.5 mM) at 30°C, 120 rpm, for 20 h, in the dark. 1DDO: TB medium, induction at OD_{600} = 1 with IPTG (1 mM) at 37°C, 120 rpm, for 24 h, in the dark.

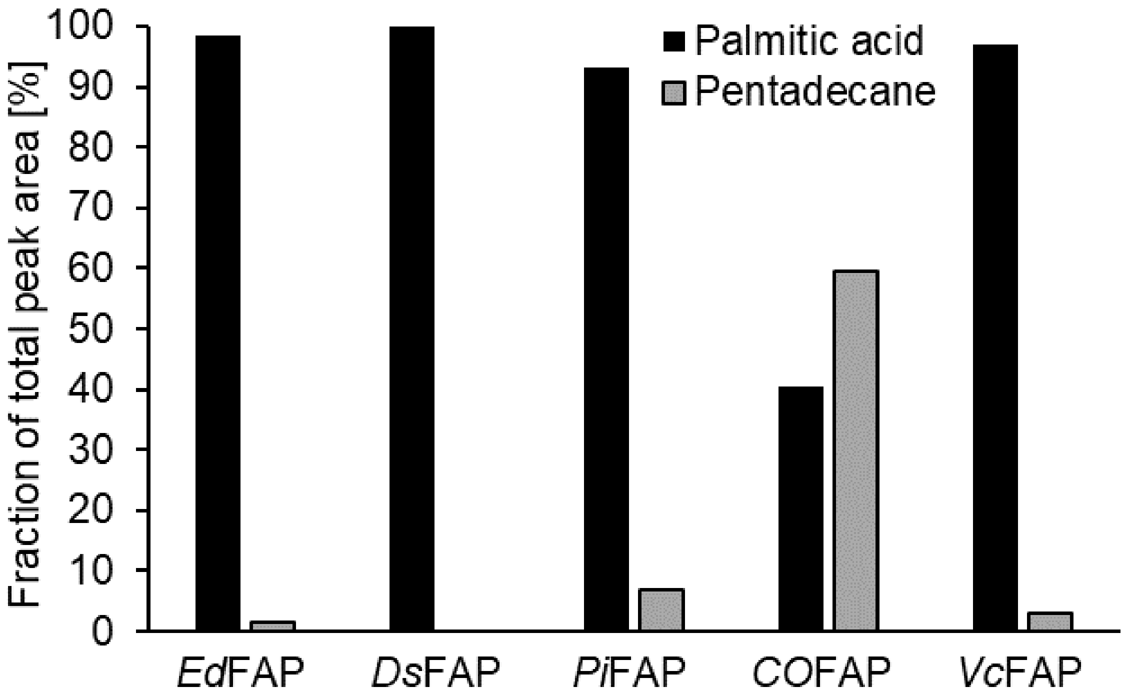

Figure S 9. Screening of activity of BLAST search candidates in the photodecarboxylation of palmitic acid. Reaction conditions: palmitic acid (10 mM), Tris·HCl buffer (100 mM, pH 8.5), lyophilized cell lysate (10 mg dry weight, for amounts of individual enzymes, see **Table S5**), DMSO (10% v/v), total volume 1 mL, 455 nm irradiation (36 µmol$L^{-1}s^{-1}$), 500 rpm, 25 °C, 20 h. The represented data corresponds to fractions of the sum peak area of substrate and product as obtained by GC-MS analysis. All data points represent mean values of duplicate determinations.

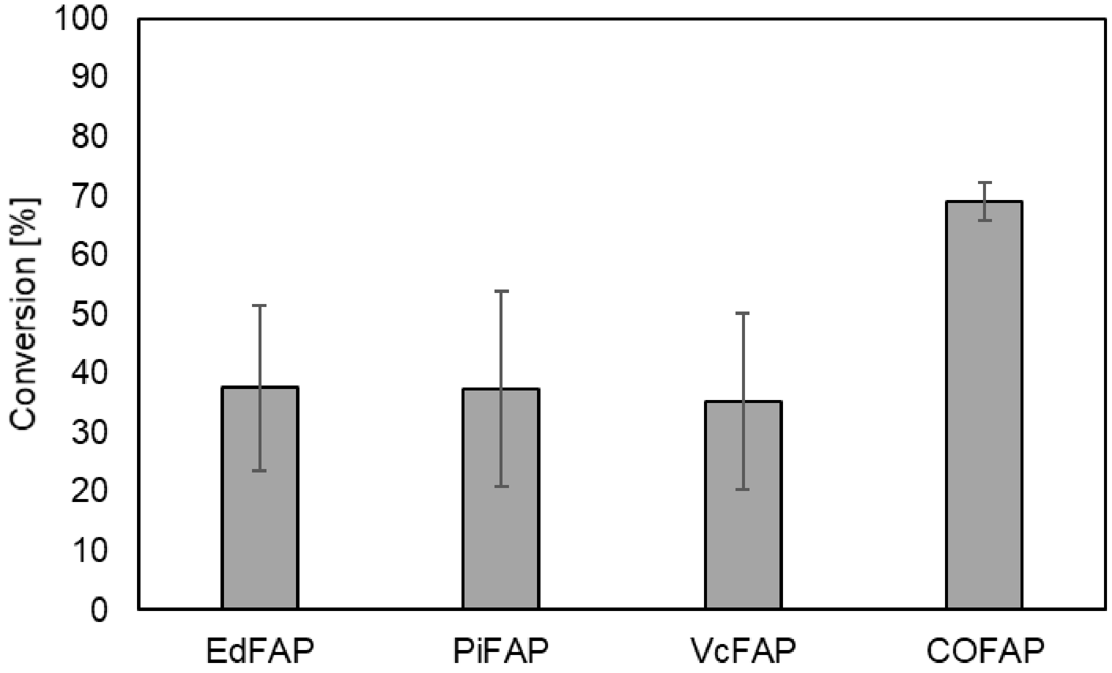

Figure S 10. Photodecarboxylation of stearic acid (C18:0) by the fatty-acid photodecarboxylases from *Edaphochlamys debaryana* (*Ed*FAP; KAG2498954.1), *Picochlorum* sp. BPE23 (*Pi*FAP; KAI8103262.1), *Volvox carteri f. nagariensis* (*Vc*FAP; XP_002948047.1) and *Coccomyxa* sp. Obi (*CO*FAP; BDA51473.1). Reaction conditions: fatty acid (10 mM), DMSO (10% v/v), Tris·HCl buffer (100 mM, pH 8.5, 900 µL), enzyme (10 mg·mL^{-1} lyophilized cell lysate; for individual enzyme amounts, see **Table S5**), total volume 1 mL. Illumination was performed at 455 nm (36 µmol·L^{-1}·s^{-1}), 25 °C, 500 rpm, for 20 h. All data points represent mean values of triplicate determinations. Data for COFAP is from **Figure 5.**

4.1 Colorimetric activity assay curves

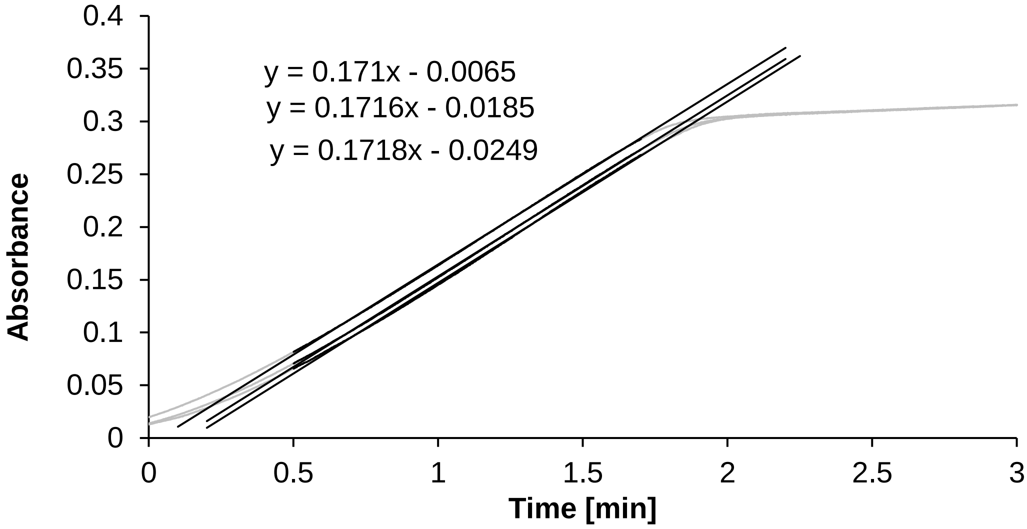

Figure S 11. Spectrophotometric kinetic measurement of the activity of L-lactate oxidase from *Aerococcus viridans*. The expression protocol was described in the literature. The cell lysate was obtained using the sonication method from a literature report.[7] The three curves and their linear parts correspond to data obtained from triplicate determinations.

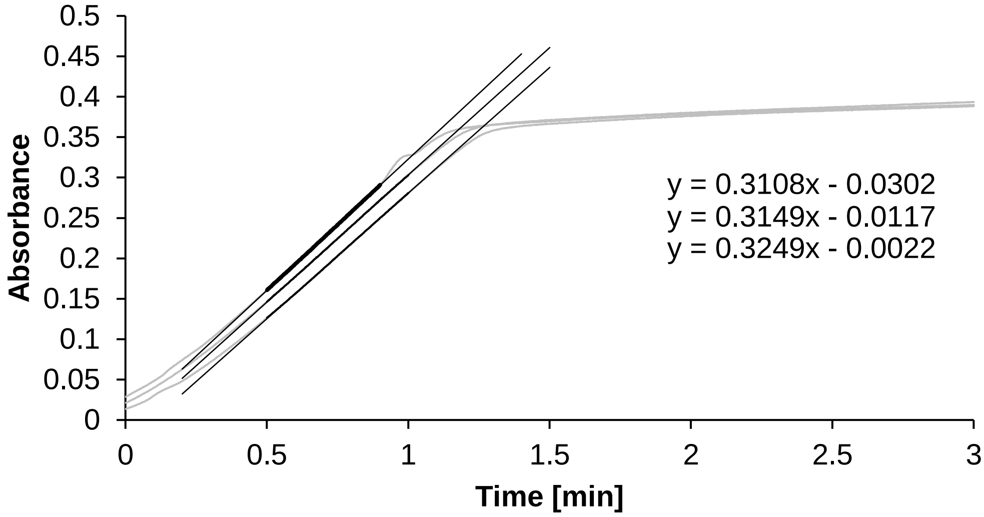

Figure S 12. Spectrophotometric kinetic measurement of the activity of L-lactate oxidase from *Aerococcus viridans*. The cell lysate was obtained using the sonication method described previously (experimental section). The three curves and their linear parts correspond to data obtained from triplicate determinations.

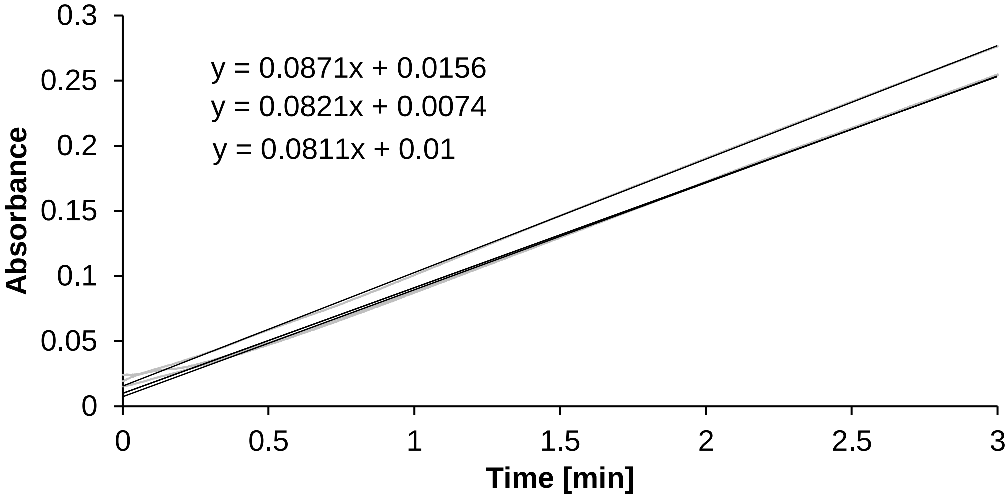

Figure S 13. Spectrophotometric kinetic measurement of the activity of L-lactate oxidase from *Aerococcus viridans*. The cell lysate was obtained from the cells cultivated under conditions described in the experimental section. The three curves correspond to data obtained from triplicate determinations.

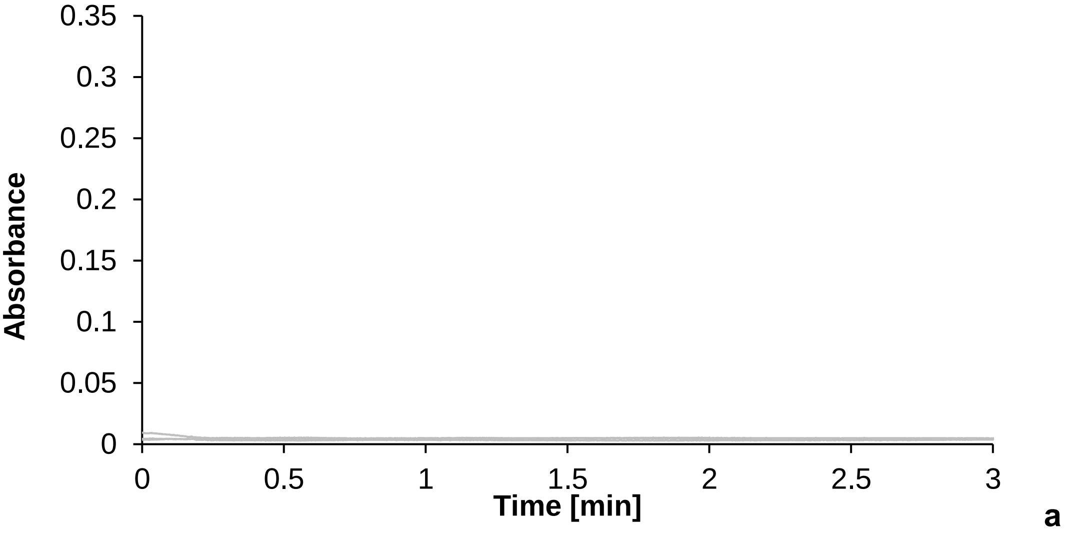

Figure S 14. Spectrophotometric kinetic measurement of the activity of *E. coli* BL21(DE3) harboring an empty vector in the L-lactate oxidase assay. The three curves correspond to data obtained from triplicate determinations.

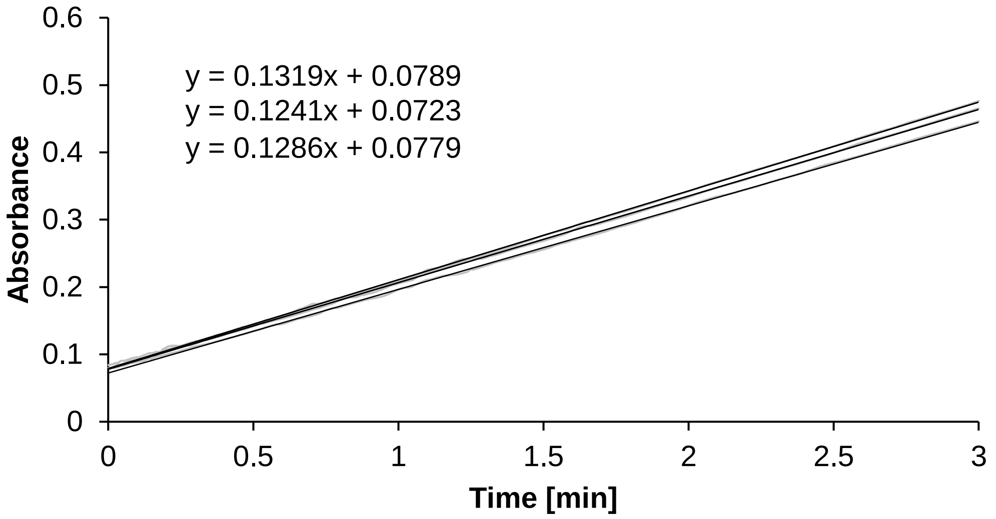

Figure S 15. Spectrophotometric kinetic measurement of the activity of cholesterol oxidase (H121A variant) from *Brevibacterium sterolicum.* The three curves correspond to data obtained from triplicate determinations.

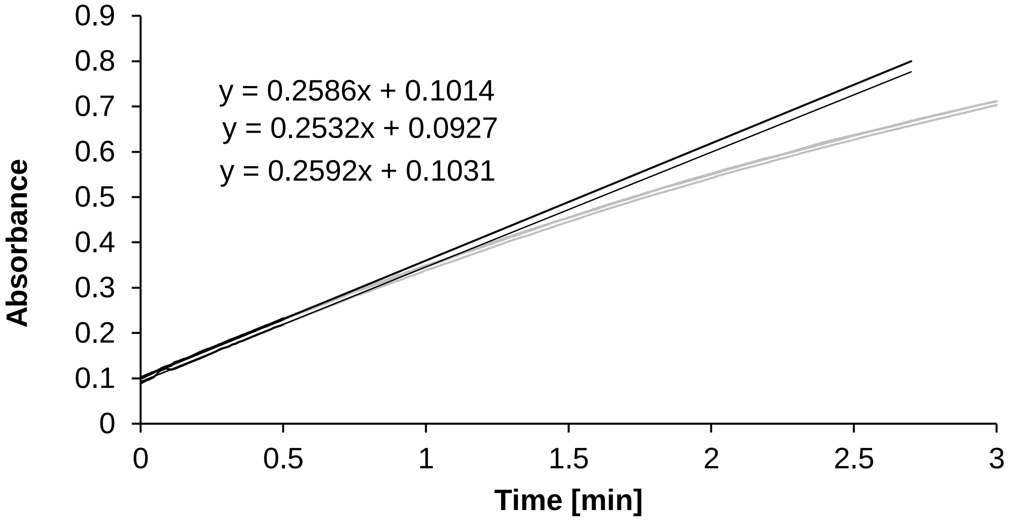

Figure S 16. Spectrophotometric kinetic measurement of the activity of solvent-stable cholesterol oxidase from *Chromobacterium* sp. DS-1*.* The three curves correspond to data obtained from triplicate determinations.

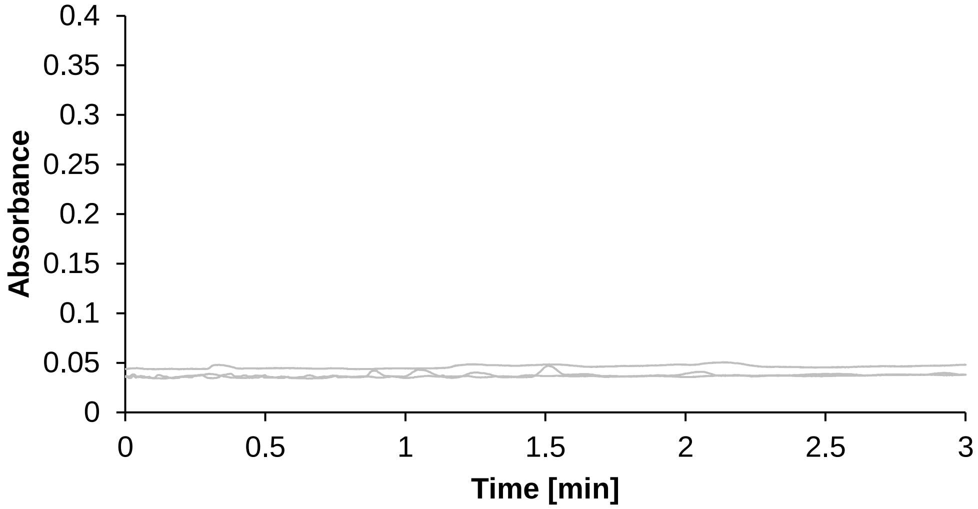

Figure S 17. Spectrophotometric kinetic measurement of the activity of *E. coli* BL21(DE3) harboring an empty vector in the cholesterol oxidase assay. The three curves correspond to data obtained from triplicate determinations.

4.2 Calibration curves

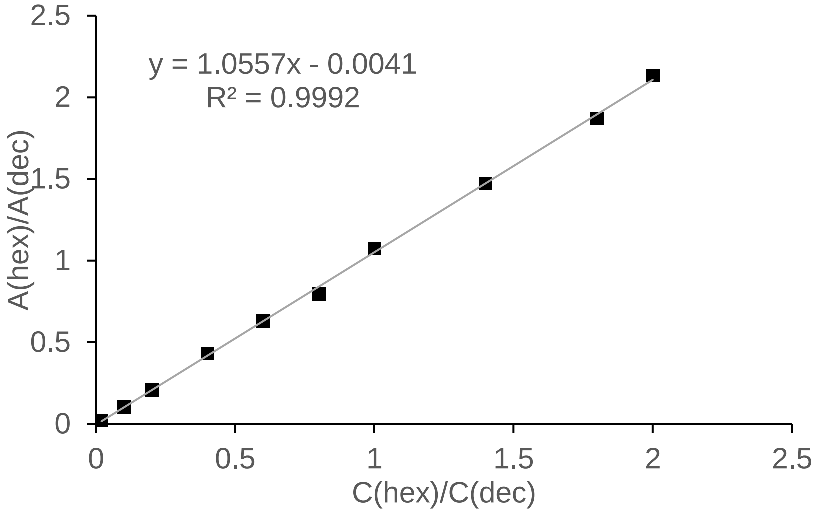

Figure S 18. Calibration curve for hexanoic acid.

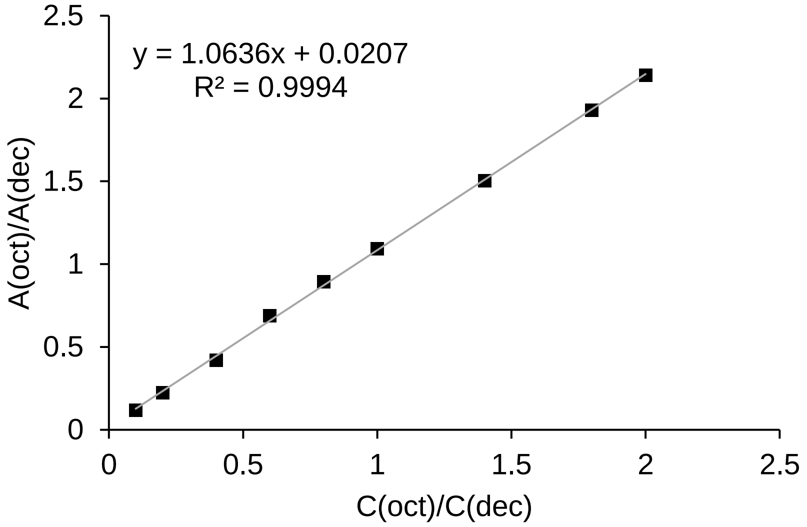

Figure S 19. Calibration curve for octanoic acid.

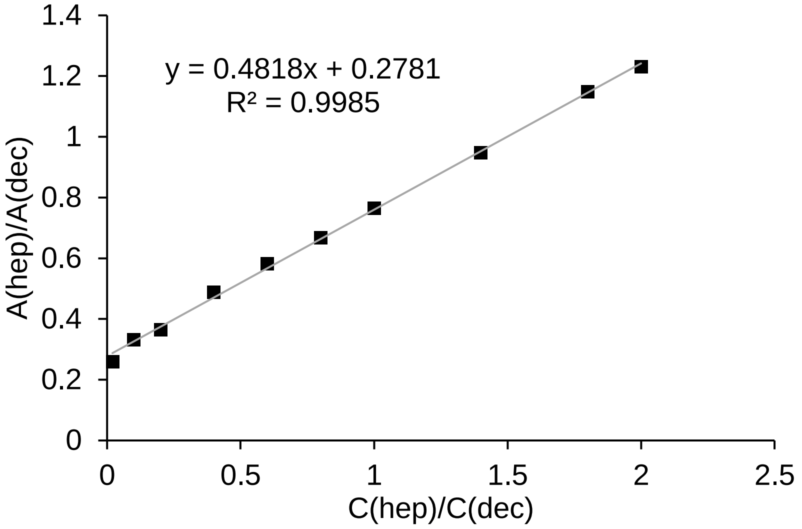

Figure S 20. Calibration curve for *n*-heptane.

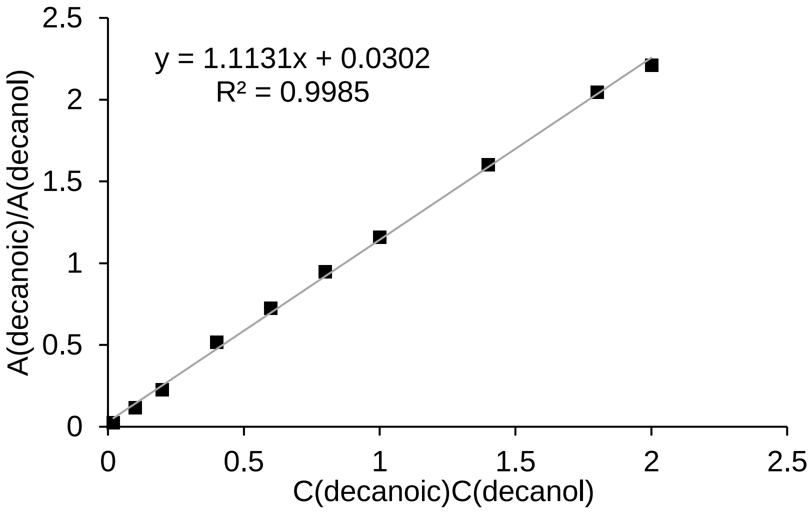

Figure S 21. Calibration curve for decanoic acid.

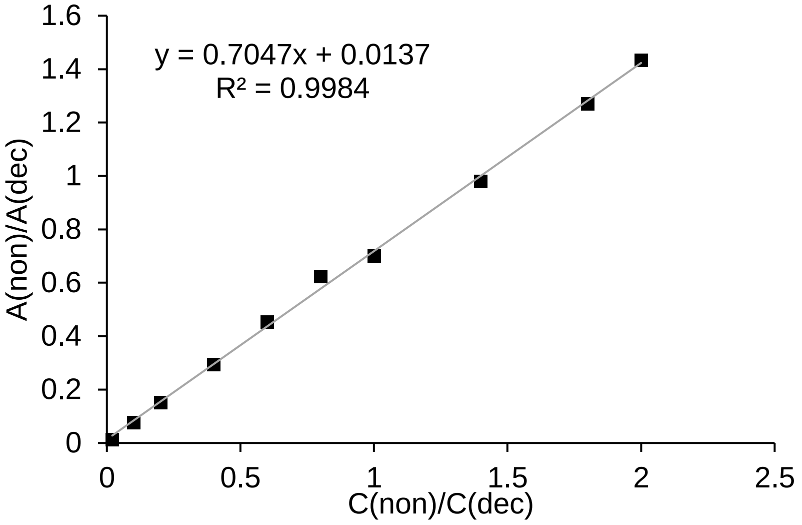

Figure S 22. Calibration curve for *n*-nonane.

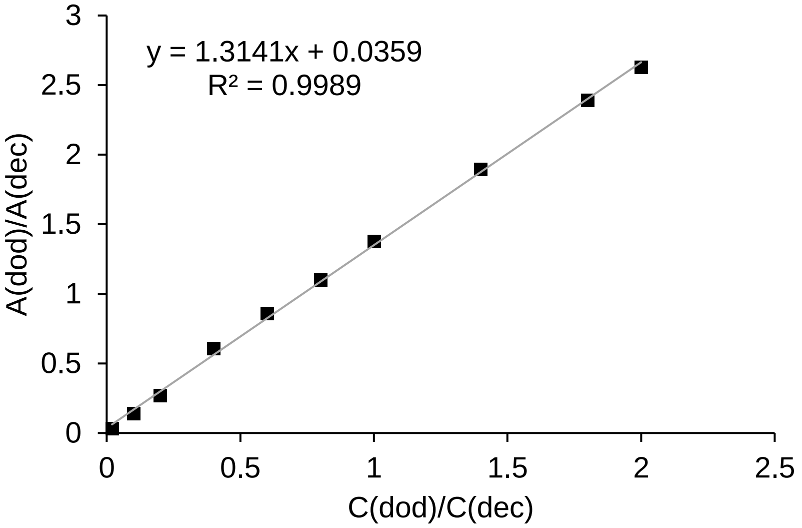

Figure S 23. Calibration curve for dodecanoic acid.

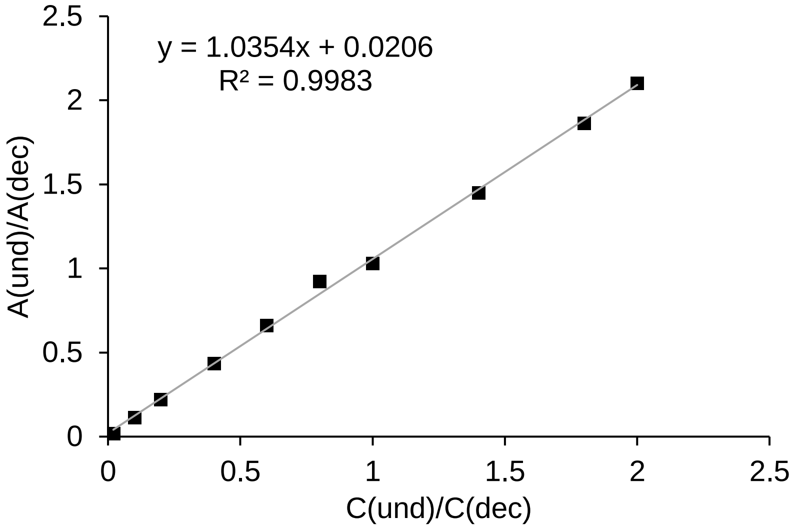

Figure S 24. Calibration curve for *n*-undecane.

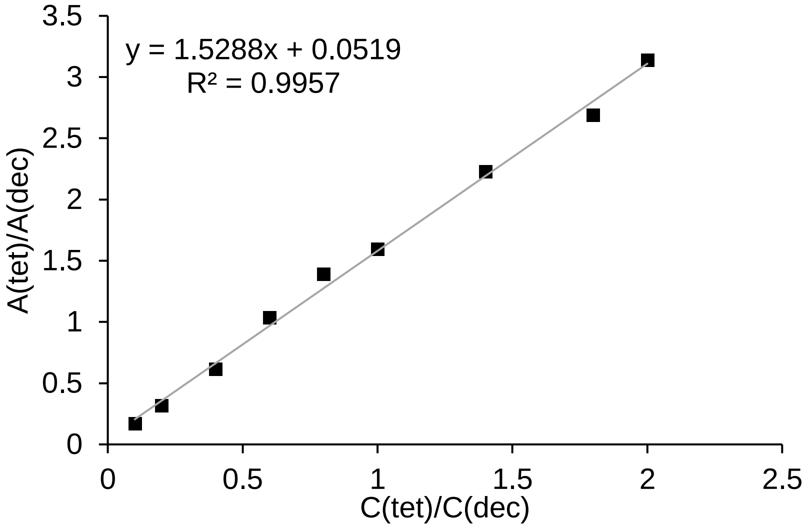

Figure S 25. Calibration curve for tetradecanoic acid.

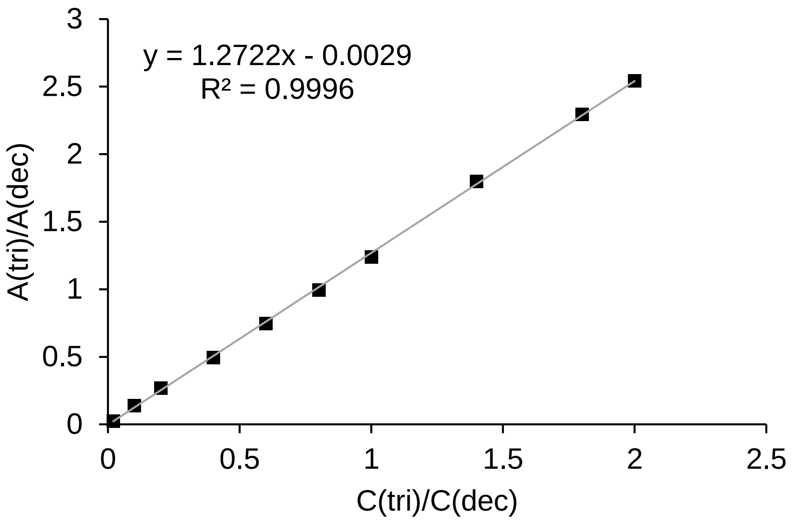

Figure S 26. Calibration curve for *n*-tridecane.

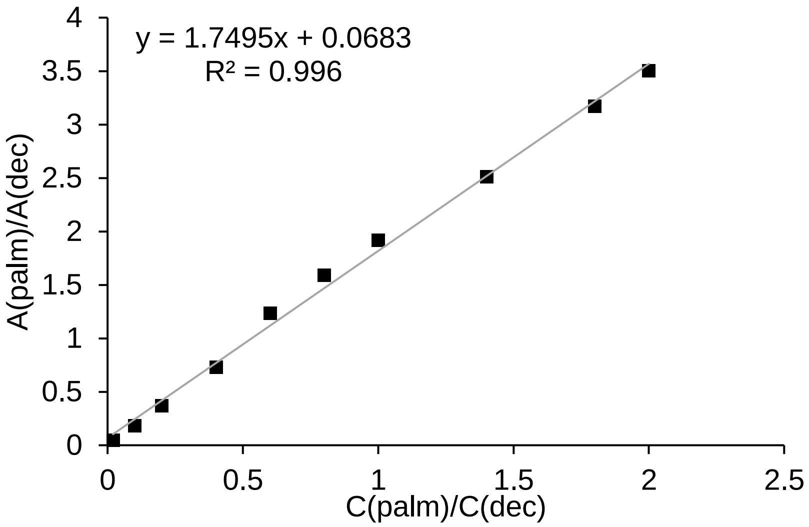

Figure S 27. Calibration curve for hexadecenoic (palmitic) acid.

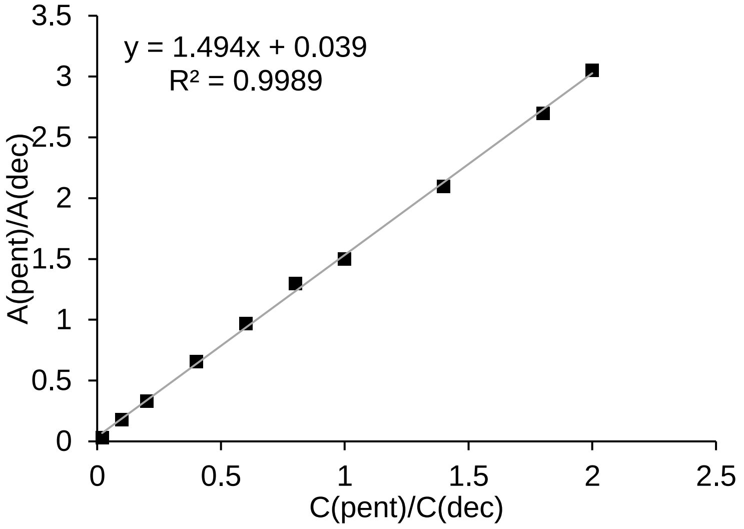

Figure S 28. Calibration curve for *n*-pentadecane.

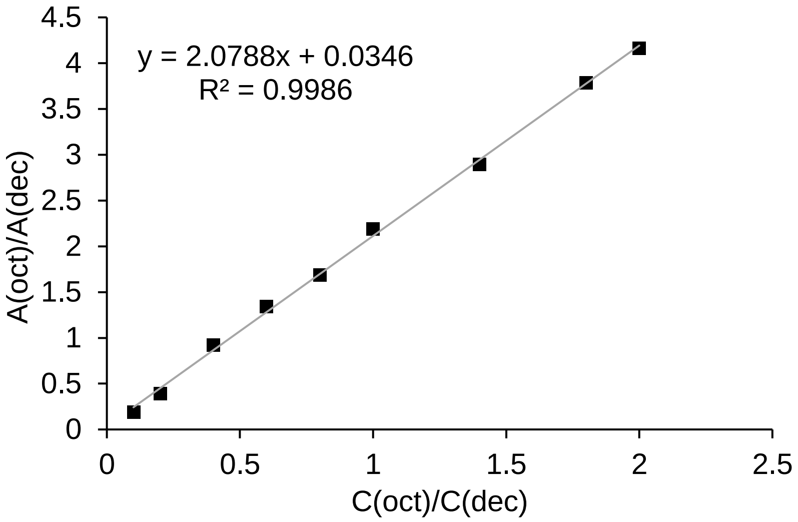

Figure S 29. Calibration curve for octadecanoic (stearic) acid.

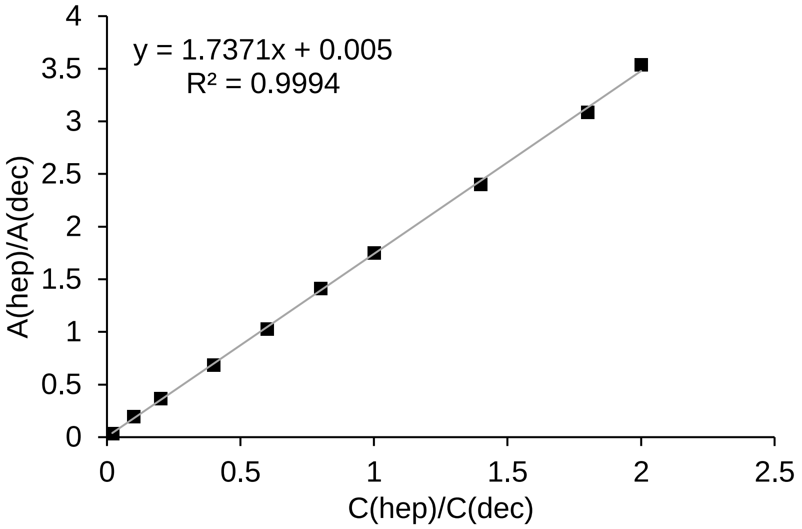

Figure S 30. Calibration curve for *n*-heptadecane.

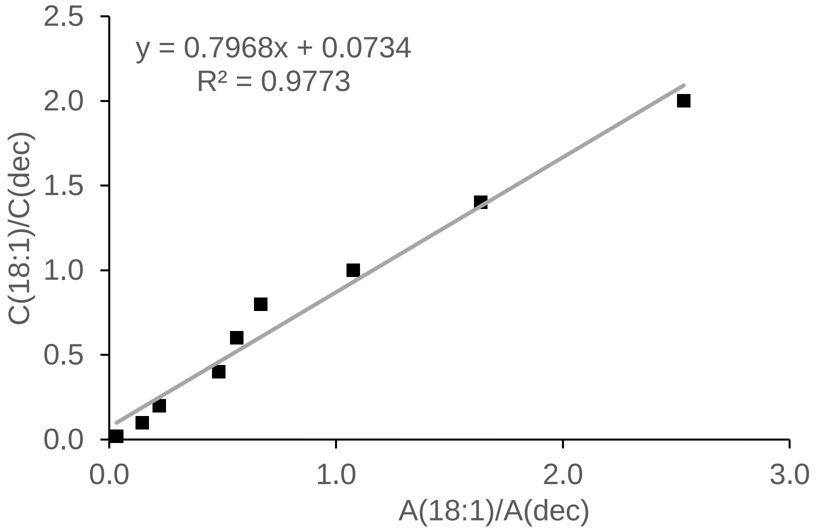

Figure S 31. Calibration curve for oleic acid (C18:1).

4.3 Chromatograms

DL-tryptophan

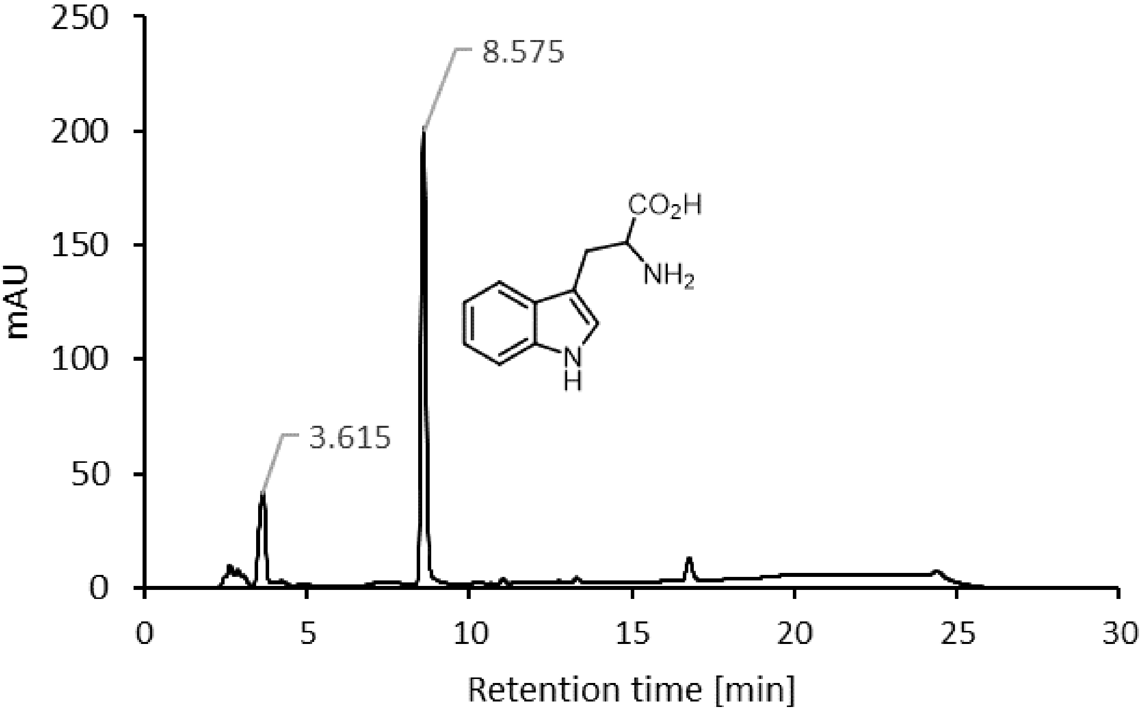

Figure S 32. Representative HPLC chromatogram of a reaction mixture containing DL-tryptophan as the starting material (UV-Vis detector, 254 nm). The reaction conditions correspond to those described in the experimental section. The peak at the retention time of 8.575 min corresponds to DL-tryptophan (base peak [M+H$^+$] = 205.2 m/z, ESI-MS). The peak at retention time of 3.615 min corresponds to a base peak of 132.2 m/z (ESI-MS).

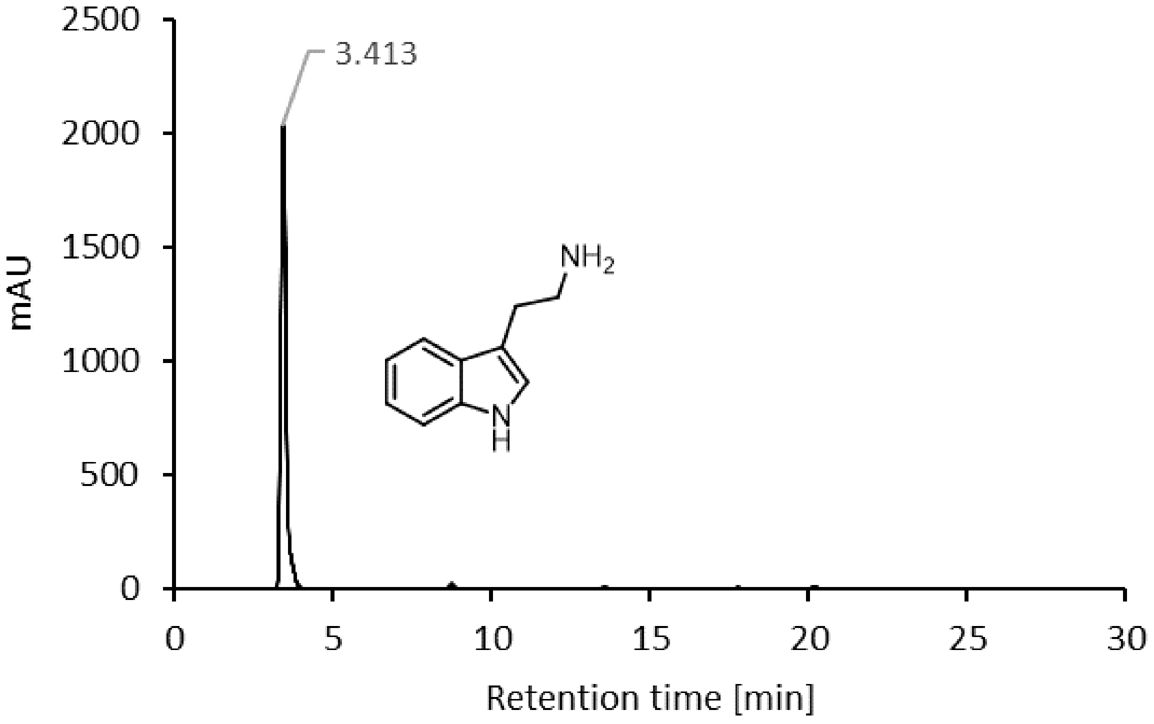

Figure S 33. Reference HPLC chromatogram (UV-Vis detector, 254 nm) of the DL-tryptophan decarboxylation product (tryptamine) standard (base peak [M+H$^+$] = 161.2 m/z, ESI-MS).

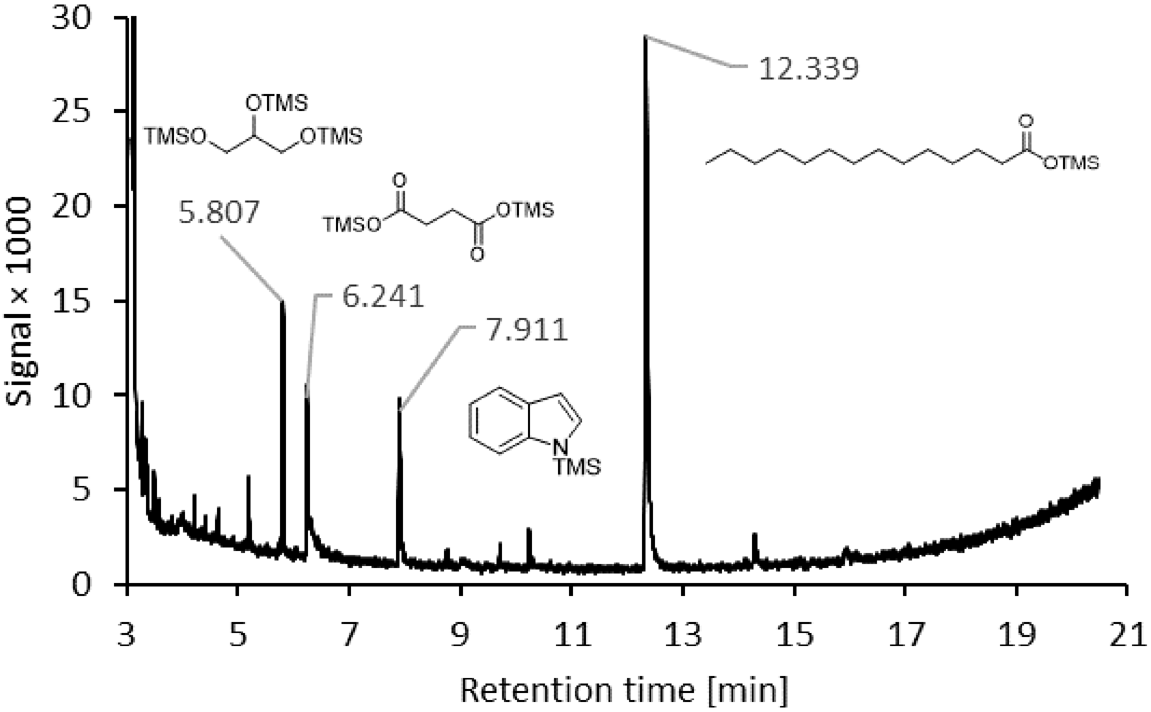

Figure S 34. Representative GC-MS chromatogram of a reaction mixture containing DL-tryptophan as the starting material. The reaction conditions correspond to those described in the experimental section. Displayed structures represent NIST database suggestions based on EI-MS fragmentation patterns.

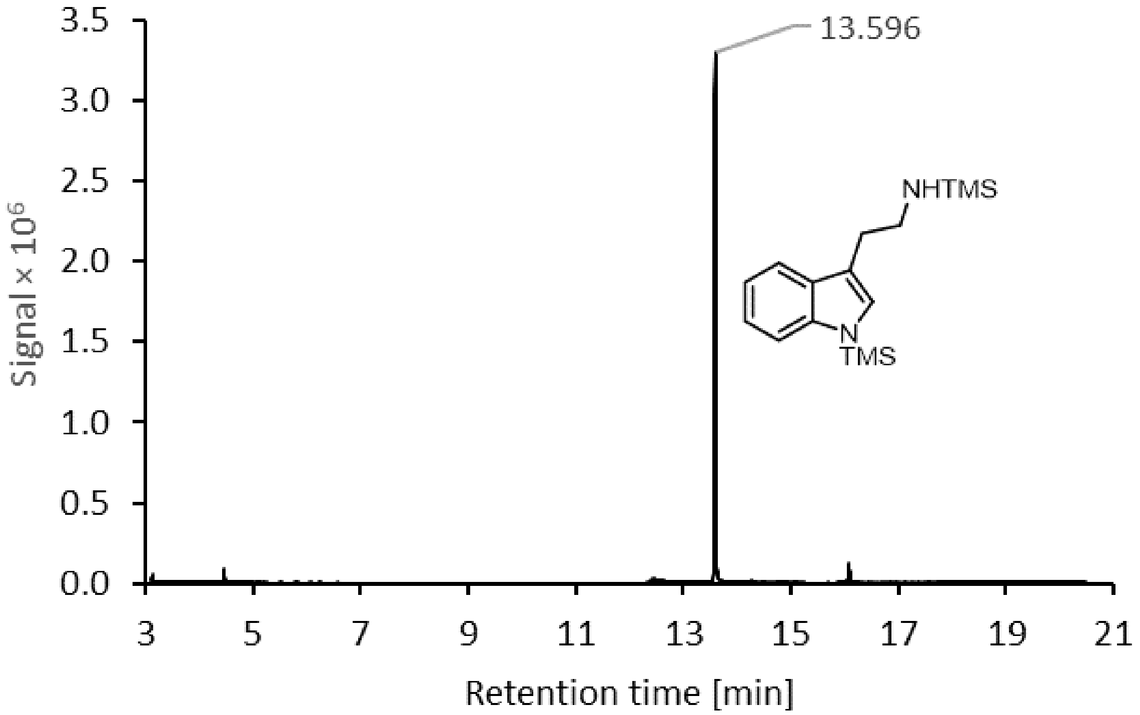

Figure S 35. Reference GC-MS chromatogram of the DL-tryptophan decarboxylation product (tryptamine) standard.

DL-phenylalanine

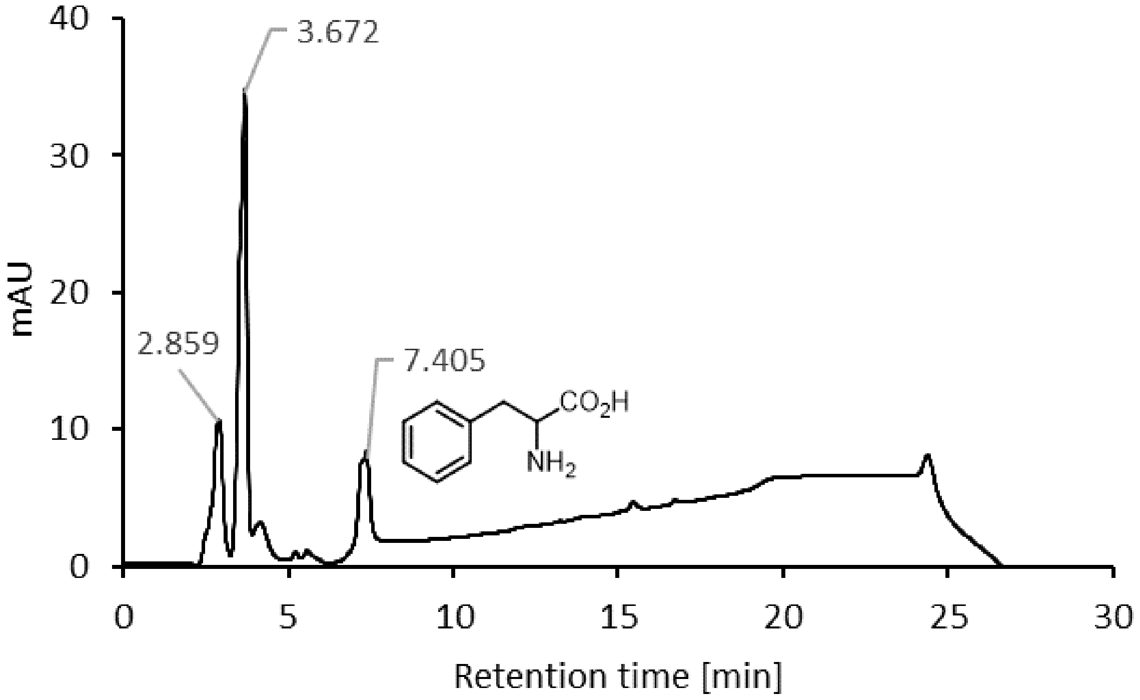

Figure S 36. Representative HPLC-MS chromatogram of a reaction mixture containing DL-phenylalanine as the starting material (UV-Vis detector, 254 nm). The reaction conditions correspond to those described in the experimental section. The peak at the retention time of 7.405 min corresponds to DL-phenylalanine (base peak [$M+H^+$] = 166.2 m/z, ESI-MS). The peak at retention time of 3.672 min corresponds to a base peak of 132.2 m/z (ESI-MS). The peak at retention time of 2.859 min corresponds to a base peak of 197.1 m/z (ESI-MS).

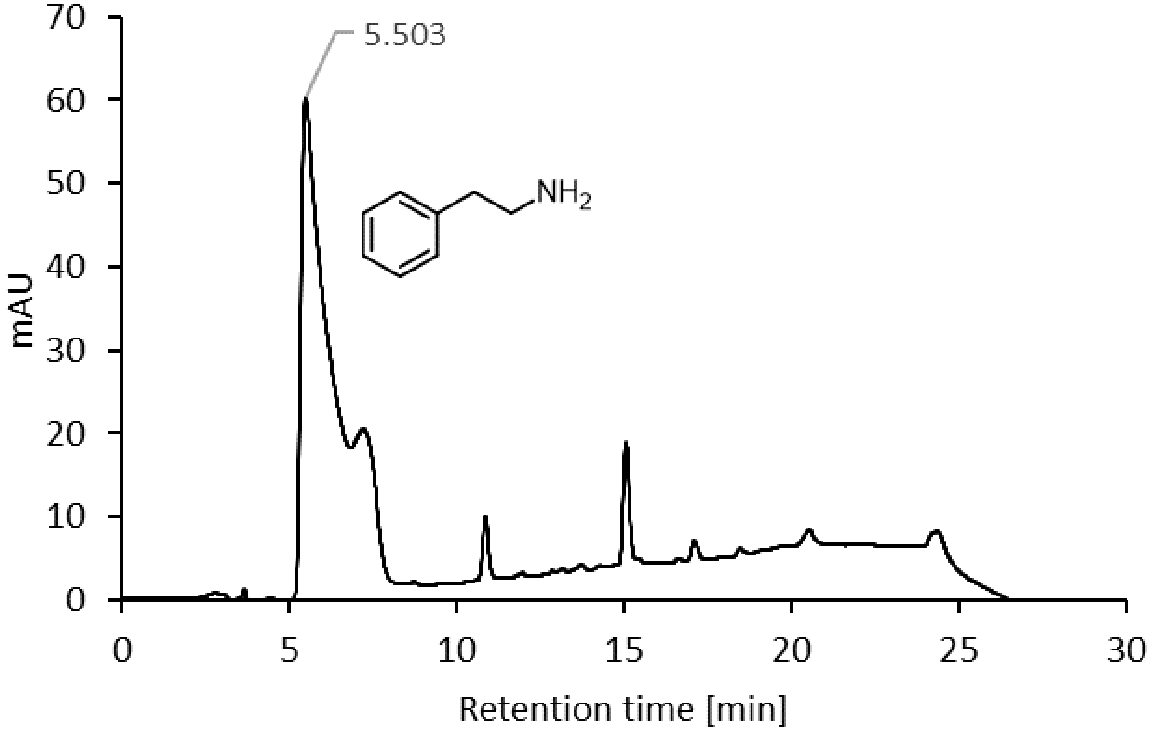

Figure S 37. Reference HPLC-MS chromatogram (UV-Vis detector, 254 nm) of the DL-phenylalanine decarboxylation product (phenethylamine) standard (base peak [M+H$^+$] = 122.2 m/z, ESI-MS).

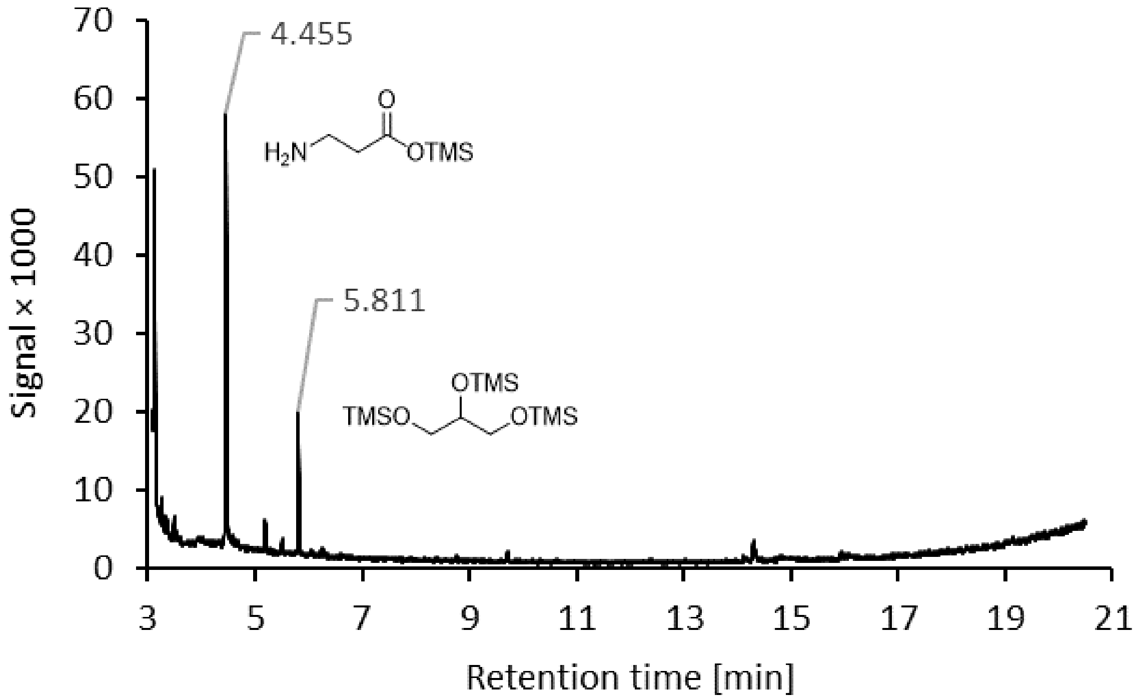

Figure S 38. Representative GC-MS chromatogram of a reaction mixture containing DL-phenylalanine as the starting material. The reaction conditions correspond to those described in the experimental section. Displayed structures represent NIST database suggestions based on EI-MS fragmentation patterns.

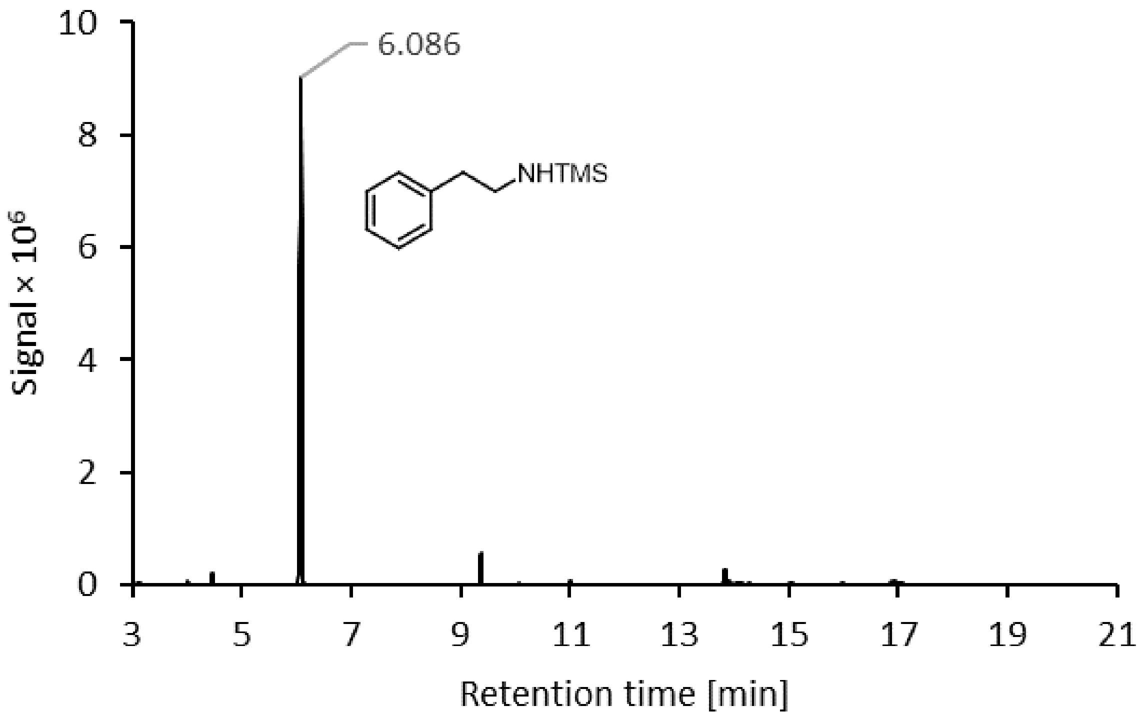

Figure S 39. Reference GC-MS chromatogram of the DL-phenylalanine decarboxylation product (phenethylamine) standard.

DL-3-phenylserine

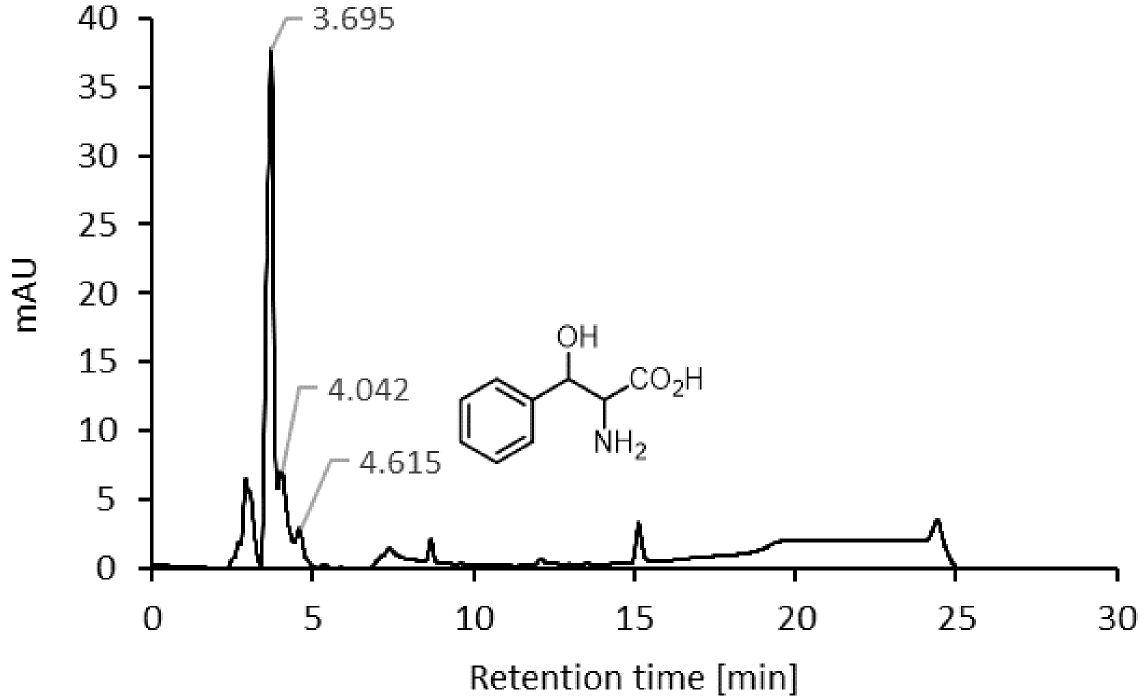

Figure S 40. Representative HPLC-MS chromatogram of a reaction mixture containing DL-3-phenylserine as the starting material (UV-Vis detector, 254 nm). The reaction conditions correspond to those described in the experimental section. The peaks at 4.042 min and 4.615 min correspond to the starting material (base peak [M+H$^+$] = 182.2 m/z, ESI-MS). The peak at 3.695 min of residence time corresponds to a base peak of 132.2 m/z.

3-phenylpropanoic acid

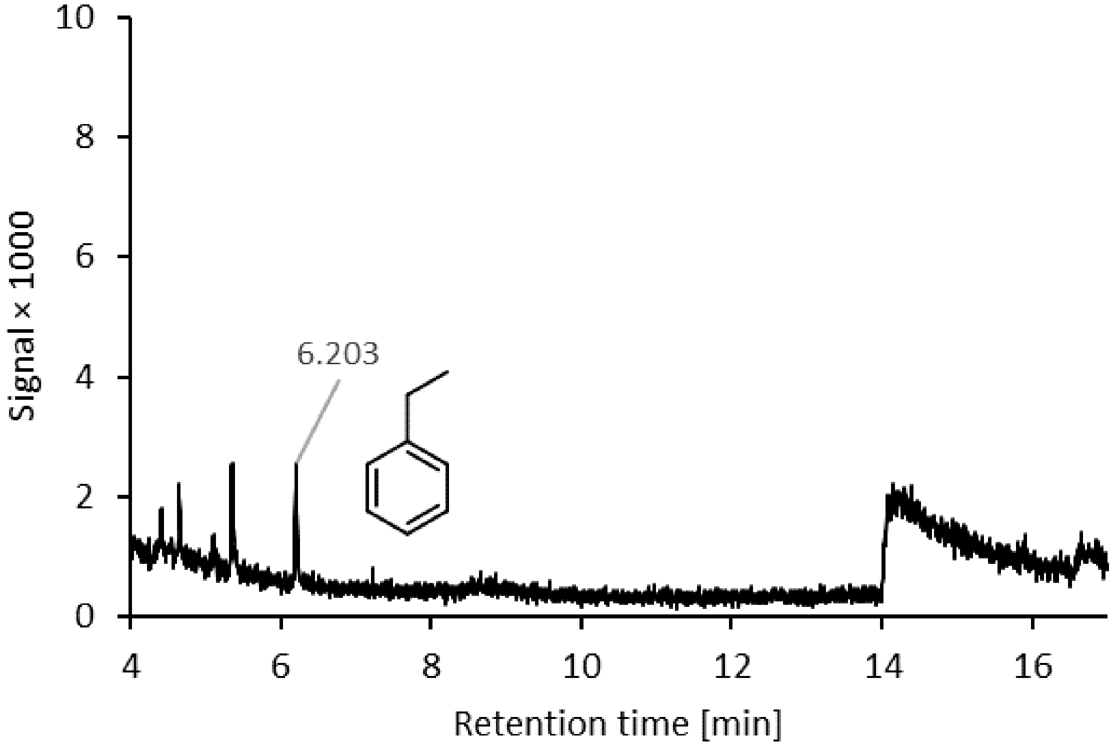

Figure S 41. GC-MS chromatogram of a reaction mixture containing ethylbenzene as the product of photodecarboxylation of 3-phenylpropanoic acid. The reaction conditions correspond to those described in the experimental section. Photodecarboxylase from *Coccomyxa* sp. Obi was used as the biocatalyst. The displayed structure represents the NIST database suggestion based on EI-MS fragmentation patterns.

Palmitic acid

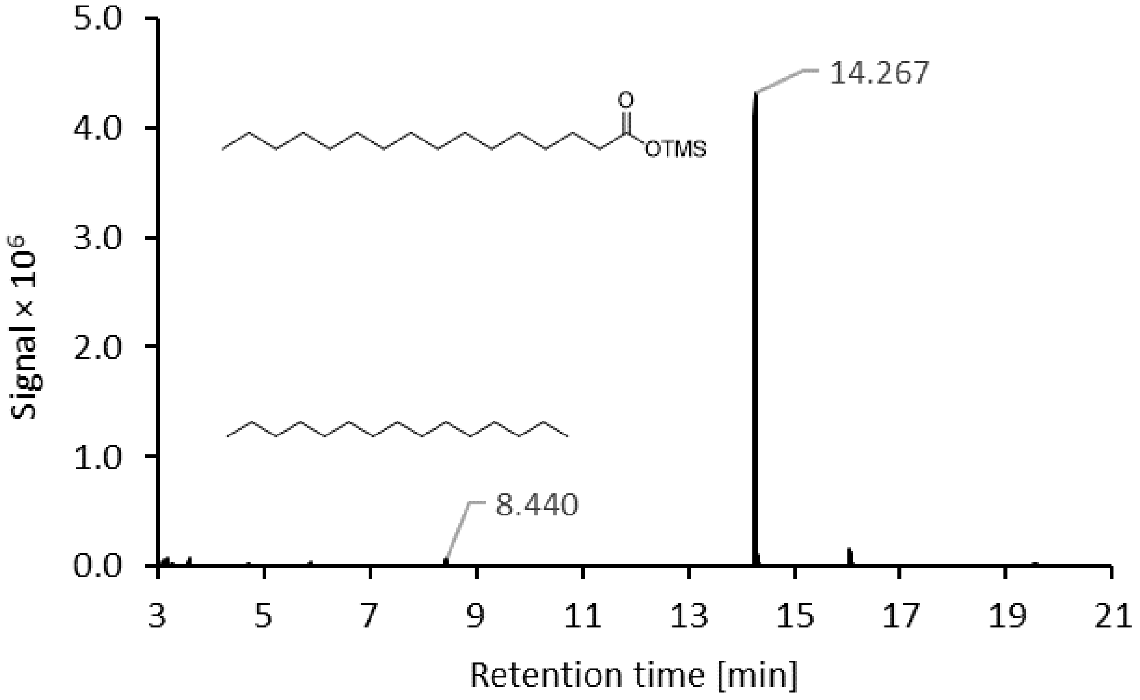

Figure S 42. GC-MS chromatogram of the reaction mixture after photodecarboxylation of palmitic acid catalyzed by GMC oxidoreductase from *Edaphochlamys debaryana* (GenBank ID: KAG2498954.1).

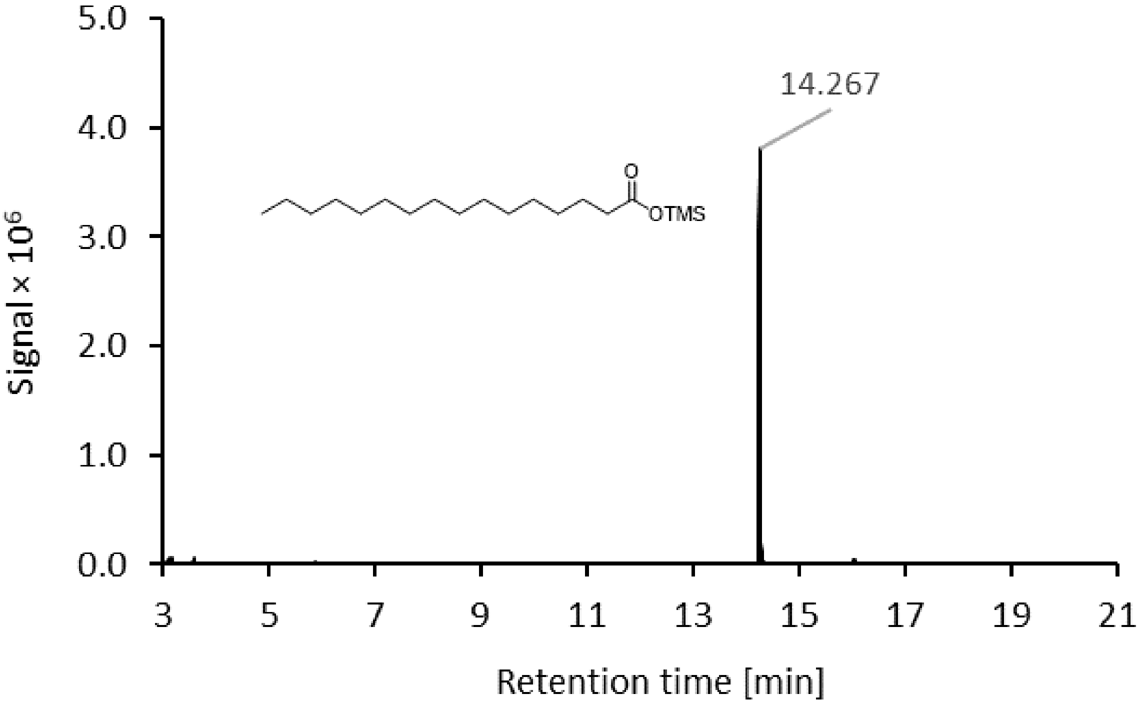

Figure S 43. GC-MS chromatogram of the photodecarboxylation reaction mixture starting from palmitic acid, in the presence of GMC oxidoreductase from *Dunaliella salina* (GenBank ID: KAF5834678.1).

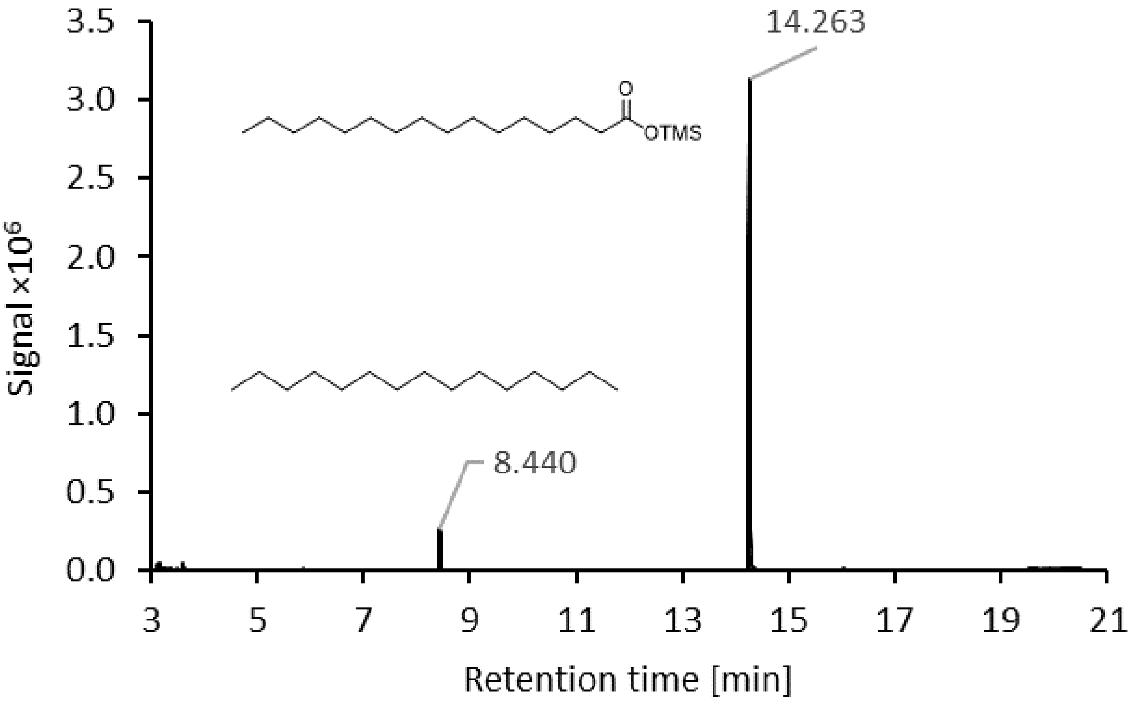

Figure S 44. GC-MS chromatogram of the reaction mixture after photodecarboxylation of palmitic acid catalyzed by a hypothetical protein from *Picochlorum* sp. BPE23 (GenBank ID: KAI8103262.1).

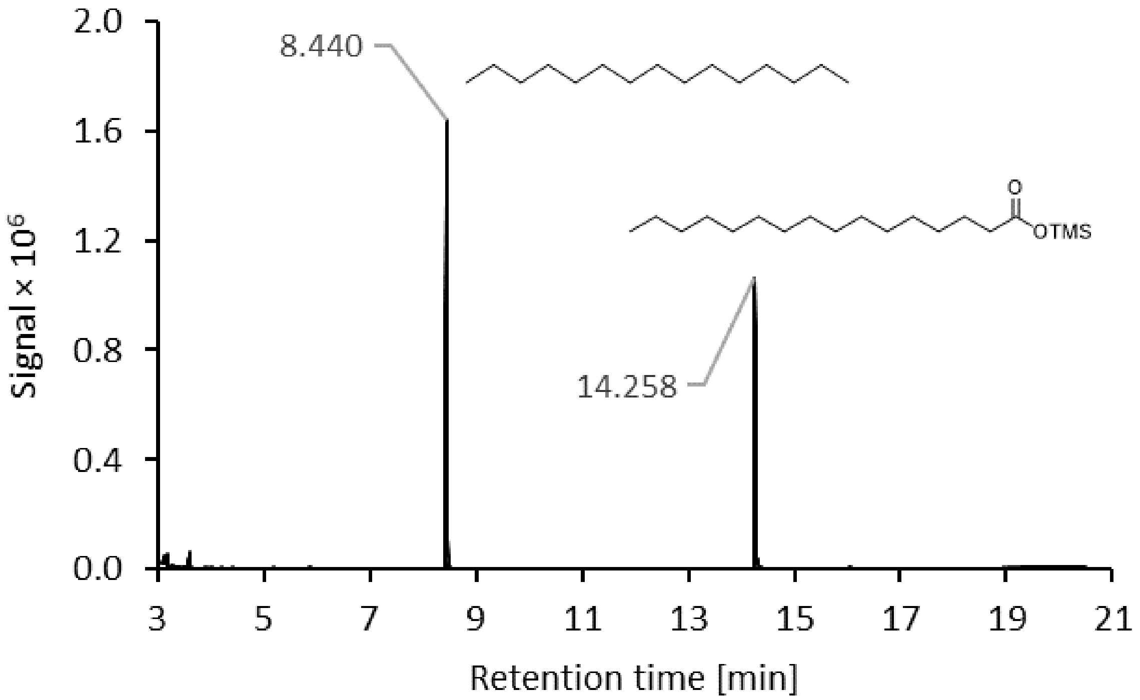

Figure S 45. GC-MS chromatogram of the reaction mixture after photodecarboxylation of palmitic acid catalyzed by GMC oxidoreductase from *Coccomyxa* sp. Obi (GenBank ID: BDA51473.1).

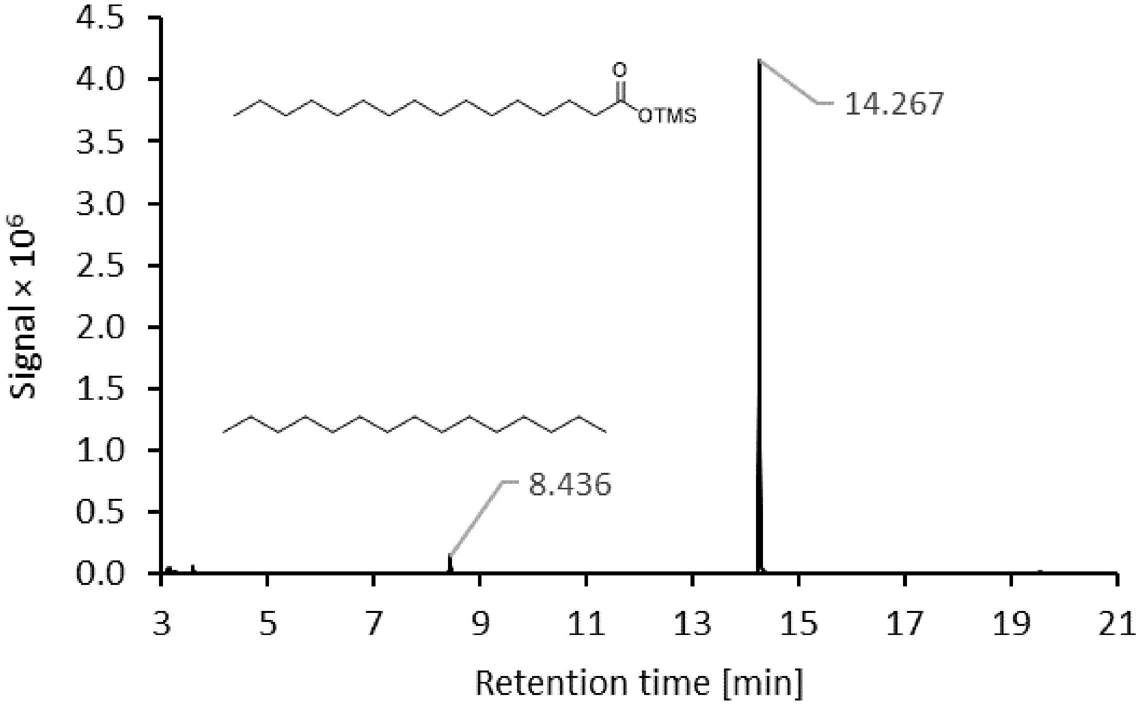

Figure S 46. GC-MS chromatogram of the reaction mixture after photodecarboxylation of palmitic acid catalyzed by GMC oxidoreductase from *Volvox carteri* (GenBank ID: XP_002948047.1).

4.4 Mass spectra

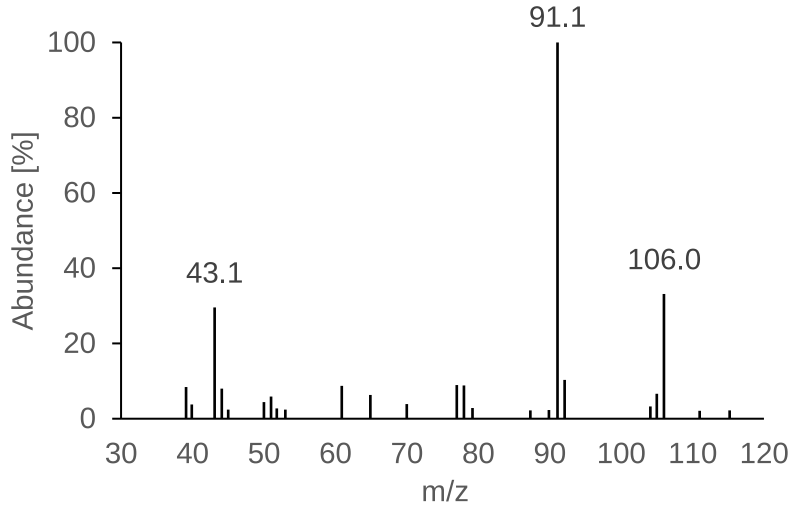

Figure S 47. EI-MS spectrum of ethylbenzene

5 Calibration of fatty acids and alkanes

The standard series samples for all fatty acids were prepared as shown below.

Sample	Fatty acid (100 mM) in DMSO (µL)	DMSO (µL)	Tris·HCl (100 mM, pH 8.5) (µL)	Final concentration (mM)
1	1	99	900	0.1
2	5	95	900	0.5
3	10	90	900	1
4	20	80	900	2
5	30	70	900	3
6	40	60	900	4
7	50	50	900	5
8	70	30	900	7
9	90	10	900	9
10	100	0	900	10

The workup and analysis of fatty acid samples was performed as described previously.
The standard series samples for all alkanes were prepared in *n*-decanol (5 mM in EtOAc) as shown below.

Sample	Alkane (20 mM) in *n*-decanol (5 mM in EtOAc) (µL)	*n*-decanol (5 mM in EtOAc) (µL)	Final concentration (mM)
1	5	495	0.2
2	25	475	1
3	50	450	2
4	100	400	4
5	150	350	6
6	200	300	8
7	250	250	10
8	350	150	14
9	450	50	18
10	500	0	20

The concentration of the analyte in the organic phase was doubled compared to the hypothetical concentration that would be found in the reaction mixture had the analyte been soluble in buffer. This accounts for the volume difference between the reaction mixture (1000

µL) and the organic phase in the first extraction step (500 µL). The organic phases were prepared in 2 mL microcentrifuge tubes and Tris·HCl buffer (100 mM, pH 8.5, 900 µL), DMSO (100 µL) and $HCl_{(aq)}$ (6 M, 50 µL) were added to mimic exact conditions during the workup of real samples. Further steps were identical to those described previously.

The standard series samples for hexanoic acid were prepared as shown below.

**Sample**	**Hexanoic acid (10 mM) in Tris·HCl [µL]**	**Tris·HCl (100 mM, pH 8.5) [µL]**	**Final concentration [mM]**
1	10	990	0.1
2	50	950	0.5
3	100	900	1
4	200	800	2
5	300	700	3
6	400	600	4
7	500	500	5
8	700	300	7
9	900	100	9
10	1000	0	10

The workup and analysis of hexanoic acid samples were performed as described previously.

6 References

[1] D. Sorigue, K. Hadjidemetriou, S. Blangy, G. Gotthard, A. Bonvalet, N. Coquelle, P. Samire, A. Aleksandrov, L. Antonucci, A. Benachir, S. Boutet, M. Byrdin, M. Cammarata, S. Carbajo, S. Cuine, R. B. Doak, L. Foucar, A. Gorel, M. Grunbein, E. Hartmann, R. Hienerwadel, M. Hilpert, M. Kloos, T. J. Lane, B. Legeret, P. Legrand, Y. Li-Beisson, S. L. Y. Moulin, D. Nurizzo, G. Peltier, G. Schiro, R. L. Shoeman, M. Sliwa, X. Solinas, B. Zhuang, T. R. M. Barends, J. P. Colletier, M. Joffre, A. Royant, C. Berthomieu, M. Weik, T. Domratcheva, K. Brettel, M. H. Vos, I. Schlichting, P. Arnoux, P. Muller, F. Beisson, *Science* **2021**, *372*, eabd5687.

[2] M. Hetmann, C. Langner, V. Durmaz, M. Cespugli, K. Kochl, A. Krassnigg, K. Blaschitz, S. Groiss, M. Loibner, D. Ruau, K. Zatloukal, K. Gruber, G. Steinkellner, C. C. Gruber, *Sci. Rep.* **2023**, *13*, 11783.

[3] M. Hendlich, F. Rippmann, G. Barnickel, *J Mol Graph Model* **1997**, *15*, 359-363, 389.

[4] R. Huey, G. M. Morris, A. J. Olson, D. S. Goodsell, *J. Comput. Chem.* **2007**, *28*, 1145-1152.

[5] K. S. Arun, T. S. Huang, S. D. Blostein, *IEEE Trans. Pattern. Anal. Mach. Intell.* **1987**, *9*, 698-700.

[6] E. Rosini, L. Caldinelli, L. Piubelli, *Front. Mol. Biosci.* **2018**, *4*, 102.

[7] S. Gandomkar, A. Dennig, A. Dordic, L. Hammerer, M. Pickl, T. Haas, M. Hall, K. Faber, *Angew. Chem. Int. Ed.* **2018**, *57*, 427-430.

[8] C. K. Winkler, W. Kroutil, Open Source Photoreactor for Parallel Evaluation of Small-Scale Reactions, can be found under https://github.com/stoeffel85/photoreactor, (accessed: 01.04.2021).

[9] C. K. Winkler, S. Simić, V. Jurkaš, S. Bierbaumer, L. Schmermund, S. Poschenrieder, S. A. Berger, E. Kulterer, R. Kourist, W. Kroutil, *ChemPhotoChem* **2021**, *5*, 957-965.

[10] National Library of Medicine, Genbank, can be found under https://www.ncbi.nlm.nih.gov/genbank/, **2024** (accessed: 12.02.2024).

[11] The UniProt Consortium, *Nucleic Acids Res.* **2023**, *51*, D523-D531.

[12] H. M. Berman, J. Westbrook, Z. Feng, G. Gilliland, T. N. Bhat, H. Weissig, I. N. Shindyalov, P. E. Bourne, *Nucleic Acids Res.* **2000**, *28*, 235-242.

[13] T. Stoisser, D. Rainer, S. Leitgeb, D. K. Wilson, B. Nidetzky, *The FEBS Journal* **2015**, *282*, 562-578.

[14] B. Unterweger, T. Stoisser, S. Leitgeb, R. Birner-Grünberger, B. Nidetzky, *Bioconjugate Chemistry* **2012**, *23*, 1406-1414.

[15] M. Sagermann, A. Ohtaki, K. Newton, N. Doukyu, *Journal of structural biology* **2010**, *170*, 32-40.

[16] P. Newton-Vinson, F. Hubalek, D. E. Edmondson, *Protein expression and purification* **2000**, *20*, 334-345.

[17] G. Lu, T. Unge, J. B. Owera-Atepo, J. C. Shih, J. Ekblom, L. Oreland, *Protein Expression and Purification* **1996**, *7*, 315-322.

[18] T. Kawazoe, H. Tsuge, M. S. Pilone, K. Fukui, *Protein Sci.* **2006**, *15*, 2708-2717.

[19] K. Yasukawa, S. Nakano, Y. Asano, *Angewandte Chemie* **2014**, *126*, 4517-4520.

[20] M. Li, C. Binda, A. Mattevi, D. E. Edmondson, *Biochemistry* **2006**, *45*, 4775-4784.

[21] K. Yasukawa, S. Nakano, Y. Asano, *Angew. Chem. Int. Ed.* **2014**, *53*, 4428-4431.

[22] F. Sievers, A. Wilm, D. Dineen, T. J. Gibson, K. Karplus, W. Li, R. Lopez, H. McWilliam, M. Remmert, J. Söding, *Mol. Syst. Biol.* **2011**, *7*, 539.

[23] M. Goujon, H. McWilliam, W. Li, F. Valentin, S. Squizzato, J. Paern, R. Lopez, *Nucleic Acids Res.* **2010**, *38*, W695-W699.

[24] A. M. Waterhouse, J. B. Procter, D. M. Martin, M. Clamp, G. J. Barton, *Bioinformatics* **2009**, *25*, 1189-1191.

[25] S. Gandomkar, A. Dennig, A. Dordic, L. Hammerer, M. Pickl, T. Haas, M. Hall, K. Faber, *Angew. Chem. Int. Ed.* **2018**, *57*, 427-430.

[26] T. Kawazoe, H. Tsuge, M. S. Pilone, K. Fukui, *Protein Sci.* **2006**, *15*, 2708-2717.

[27] K. Yasukawa, S. Nakano, Y. Asano, *Angew. Chem. Int. Ed.* **2014**, *53*, 4428-4431.
